# Genome-wide Association Study for Yield and Yield-Related Traits in Diverse Blackgram Panel (*Vigna mungo* L. Hepper) Reveals Novel Putative Alleles for Future Breeding Programs

**DOI:** 10.3389/fgene.2022.849016

**Published:** 2022-07-11

**Authors:** Lovejit Singh, Guriqbal Singh Dhillon, Sarabjit Kaur, Sandeep Kaur Dhaliwal, Amandeep Kaur, Palvi Malik, Ashok Kumar, Ranjit Kaur Gill, Satinder Kaur

**Affiliations:** ^1^ Department of Plant Breeding and Genetics, Punjab Agricultural University, Ludhiana, India; ^2^ School of Agricultural Biotechnology, Punjab Agricultural University, Ludhiana, India; ^3^ Regional Research Station, Punjab Agricultural University, Gurdaspur, India

**Keywords:** *Vigna mungo*, heritability, yield, GWAS, MTA, QTL, allelic effects

## Abstract

Blackgram (*Vigna mungo* L. Hepper) is an important tropical and sub-tropical short-duration legume that is rich in dietary protein and micronutrients. Producing high-yielding blackgram varieties is hampered by insufficient genetic variability, absence of suitable ideotypes, low harvest index and susceptibility to biotic-abiotic stresses. Seed yield, a complex trait resulting from the expression and interaction of multiple genes, necessitates the evaluation of diverse germplasm for the identification of novel yield contributing traits. Henceforth, a panel of 100 blackgram genotypes was evaluated at two locations (Ludhiana and Gurdaspur) across two seasons (*Spring* 2019 and *Spring* 2020) for 14 different yield related traits. A wide range of variability, high broad-sense heritability and a high correlation of grain yield were observed for 12 out of 14 traits studied among all environments. Investigation of population structure in the panel using a set of 4,623 filtered SNPs led to identification of four sub-populations based on ad-hoc delta K and Cross entropy value. Using Farm CPU model and Mixed Linear Model algorithms, a total of 49 significant SNP associations representing 42 QTLs were identified. Allelic effects were found to be statistically significant at 37 out of 42 QTLs and 50 known candidate genes were identified in 24 of QTLs.

## Introduction

Blackgram (*Vigna mungo* L. Hepper), a diploid (2n = 2X = 22), short duration legume crop of family Leguminaceae, was domesticated in Northern South Asia from progenitor *Vigna mungo* var. *silvestris* (Lukoki et al., 1980). It is cultivated throughout Southeast Asia because of its multiple benefits to soil and human health. It is nutritionally important crop with about 25% protein—nearly three times that of cereals, 60% carbohydrates, 1.3% fat (Das et al., 2021) as well as important vitamins and minerals (Ghafoor et al., 2001), making it a balanced vegan diet when supplemented with cereals. The ability of its roots to fix atmospheric nitrogen (42 kg/ha/year) (Dey et al., 2020) contribute towards soil health while deep-roots prevents soil erosion by binding soil particles. Short duration of blackgram makes it suitable for intercropping with corn or millet or rotation with cereals like rice or wheat (Muthusamy and Pandiyan, 2018), adding another benefit for farmer.

India is the largest producer and consumer of blackgram as it is colossally grown in almost every agro-climatic zone (Raizada and Souframanien, 2021). However, the crop accounts for only 13% of the total area (56.02 lakh hectares) and 10% of total pulses production (30.60 lakh tons) in India (Muthusamy and Pandiyan, 2018) with productivity of 5.46 quintals per hectare (Singh et al., 2020). Moreover, around 2-3 million tons of pulses are imported annually to fulfill the domestic consumption requirement. Despite the economic and nutritional value of black Gram, the sluggish growth in production is due to lack of commercialized market setup, multiple biotic stresses (mosaic, seedling blight, leaf blight, leaf crinkle virus, leaf folder, Bihar hairy caterpillar, whitefly) and abiotic stresses (drought, salinity, waterlogging). Photo- and thermo-sensitivity of crop with indeterminate habit of flowering and fruiting leads to competition of assimilates between vegetative and reproductive sinks throughout the growth period causing low harvest index and poor grain yield (Somta et al., 2019; Sahu et al., 2020).

The expansion of the crop is constrained by lack of genetic and genomic resources along with limited diversity (Chaitieng et al., 2006; Gupta et al., 2008; Somta et al., 2019 A systematic program of identification, genetic and genomic characterization and utilization of diverse germplasm is required for successfully decoding the genetic architecture of agronomically important traits for blackgram improvement. Genome and transcriptome sequencing (Pootakham et al., 2021; Raizada and Souframanien, 2021), developing dense molecular linkage maps, and using high-throughput genotyping techniques can widen the horizons improvement of this crop. Genotyping–by- sequencing (GBS) is one of the cost-efficient genomic techniques which includes multiplex sequencing of a subset of the genome and generates numerous SNP markers for linkage mapping (Varshney et al., 2009; Elshire et al., 2011; Noble et al., 2018). Genome wide association studies (GWAS) coupled with GBS have been promising tool for estimating the genetic diversity in different crops of soybean (Hwang et al., 2014), chickpea (Plekhanova et al., 2017), cowpea (Xu et al., 2017), pigeonpea (Varshney et al., 2009), and mungbean (Sokolkova et al., 2020) providing insights to underlying genetic architecture of complex traits (Lorenz et al., 2010; Scherer and Christensen, 2016).

In the present study we performed the GWAS on diverse blackgram germplasm panel to assess its genetic diversity and population structure, and to identify MTA (Marker trait associations) involved in yield and yield related traits using GBS. This study provides a unique genomic resource for the genetic dissection of important traits aimed at blackgram improvement.

## Materials and Methods

### Plant Material and Field Trials

A panel consisting of 100 blackgram (*V. mungo*) genotypes was used for the present study. These included 54 genotypes procured from National Bureau of Plant Genetic Resources (NBPGR), New Delhi, India, while, the remaining genotypes were from germplasm collection of Punjab Agricultural University (PAU), Ludhiana, India (Supplementary Table S1). The genotypes were evaluated using a simple lattice design (10 × 10), in two replications at two locations (Ludhiana and Gurdaspur) across two seasons (*Spring* 2019 and *Spring* 2020). The seeds were sown in a 2 m long row with 10 cm plant to plant spacing and 30 cm row to row spacing. Ludhiana (30.9°N, 75.85°E) lies in a sub-tropical zone characterized by relatively high temperatures and low precipitation while Gurdaspur region (32.02°N, 75.24°E) is characterized by lower temperature and high humidity coupled with abundant rainfall. The weekly mean temperature, relative humidity and rainfall for Ludhiana and Gurdaspur have been given in Supplementary Figure S1.

### Phenotypic Evaluation and Statistical Analysis

Data was collected in three replicates from five randomly selected plants of each genotype in each replicate for plant height at 90% pod maturity (PHM), branches per plant (BpP), nodes per plant (NpP), internodal length (IL = PHM/NpP), clusters per plant (CpP), pods per plant (PpP), pod length (PL), seeds per pod (SpP), biological yield per plant (BYpP), yield per plant (YpP), harvest index (HI) and hundred seed weight (HSW). Days to 50% flowering (DtF) and days to 90% pod maturity (DtM) were recorded based on the entire plot. For phenotypic analysis, environments were designated as E1 (Ludhiana, year 2019), E2 (Ludhiana, year 2020), E3 (Ludhiana combined for years 2019 & 2020), E4 (Gurdaspur, year 2019), E5 (Gurdaspur, year 2020) and E6 (Gurdaspur combined for years 2019 & 2020). Due to the significant differences between two selected locations, combined analysis over two selected locations has not been done. Descriptive statistical analysis across all the environments was done using Meta-R v6.0 software (Alvarado et al., 2020). Statistical analysis for individual and multi-environment was performed using “lme4” (Bates et al., 2015) and “Residual Maximum Likelihood (REML)” (Laird and Ware, 1982) methods. The linear model for analyzing individual environments for simple lattice design was done using the formula:

Yijk=μ+Repi+Blockj(Repi)+Genk+εijk(across replicates, within environment)

Yijk1=μ+Year1+Repi(Year1)+Blockj(Year1Repi)+Genk+Genk x Year1+εijk1(across replicates, across environment)

where Y_ijk_ and Y_ijkl_ represent the trait of interest, μ is the overall mean effect, Rep_i_ is the effect of *i*th replicate, Block_j_ (Rep_i_) is the effect of *j*th incomplete block within the *i*th replicate, Gen_k_ is the effect of the *k*th genotype and ε_ijk_ is the error effect associated with the *i*th replication, *j*th incomplete block and *k*th genotype, assumed to be normally distributed with zero mean and variance σ^2^ε (Alvarado et al., 2020). Year_l_ and Gen_l_ x Year_i_ are the effects of the *l*th year and Genotype x Year (G x Y) interactions represented by effect on the *i*th genotype in the *l*th year in the linear model for integrated analysis for multi-environment (across the years). The resulting analysis produced the adjusted trait phenotypic values as BLUPs (Best linear unbiased predictions) within and across environments. The genotypes are considered random effects in the BLUPs model, minimizing/eliminating the effect of the environment from phenotypic effects. The broad-sense heritability of trait at individual environment and across environments was calculated as

$$H^{2}=\frac{\sigma_{g}^{2}}{\sigma_{g}^{2}\;+\;\sigma_{e}^{2}/n\;reps}\left(\text{Across replicates, within environment}\right)$$

$$H^{2}=\;\frac{\sigma_{g}^{2}}{\frac{\sigma_{g}^{2}+\;\frac{\sigma_{ge}^{2}}{\;n\;env}\;+\;\sigma_{e}^{2}}{\left(n\;reps\;\times \;n\;env\right)}}\left(\text{Across replicates, across environment}\right)$$

Where $\sigma_{g}^{2}\;$ and $\sigma_{e}^{2}$ are the genotype, and error variance components, respectively, $\sigma_{ge}^{2}$ is genotype by environment interaction variance, n env is the number of environments, and n reps is the number of replicates. The estimated broad-sense heritability provides valuable insight into the breeding program’s quality, with all effects considered as random effects (Alvarado et al., 2020). The LSD at 5% level of significance was calculated as

LSD=t(1−0.05, dfErr) X ASED

where t is the cumulative Student’s t-distribution, 0.05 is the selected α level (5%), dfErr is the degrees of freedom for error in the linear mixed model, and ASED is the average standard error of the differences of the means. The coefficient of variation (%) was calculated as:

$$CV=\frac{\sqrt{MSE}}{Grand\;Mean}\;X\;100$$

where MSE is the mean squared error, and Grand mean is the mean of the trait. BLUPs for recorded traits in all environments were plotted using the ggplot2 v3.3.2 package (Wickham, 2016) in R v4.0.3 (Core R Team 2019).

### DNA Isolation and Genotyping

Total genomic DNA was isolated from fresh leaves of single plant of each genotype using modified cetyl trimethyl ammonium bromide (CTAB) method (Saghai-Maroof et al., 1984). Genotyping-by-sequencing (GBS) of DNA samples was outsourced to Novogene Co. Ltd., China. The GBS library was prepared using double digest restriction enzyme DNA (ddRAD) and sequencing was done with Illumina HiSeq 2000. The raw FASTQ files (obtained from Illumina pipeline CASAVA v1.8.2) were processed for quality control and filtered using Trimmomaticv0.39 software (Bolger et al., 2014) Reads with no matching barcode or cut sites overhangs, having more than 10% unidentified bases (N), with adapter dimers, with lower quality reads, and with Q_phred_ score less than 34, were excluded from further analysis. High-quality paired end reads were aligned using Burrows-Wheeler Aligner (BWA) (Li et al., 2009) to *Vigna radiata* reference genome (ftp://ftp.ncbi.nlm.nih.gov/genomes/all/GCF/000/741/045/GCF_000741045.1_Vradiata ver6). A quality threshold score of 10 was applied to validate SNP loci (Wu et al., 2020). Sorted binary alignment map (BAM) files were converted to variant caller format (vcf) files using mpileup function of bcftools v1.10.2 in samtools v1.10 software package (Li et al., 2009) with minimum read depth ≥4. Haplotype map (hapmap) format files were generated from vcf files using Tassel v5.0 software (Bradbury et al., 2007). After SNP calling, raw hapmap file was filtered by removing indels, minor allele frequency (maf) > 0.05, genotype missing data less than 10% and heterozygosity less than 30% (Torkamaneh et al., 2020). The generated raw reads were submitted to the NCBI sequence read archives (SRA) with accession number PRJNA802066.

### Population Structure and Phylogeny Analysis

Bayesian-based approach in STRUCTURE v2.3.4 software (Pritchard et al., 2000) using a burn-in period of 10,000 and Markov chain Monte Carlo iterations of 100,000 for k ranging from 1 to 8 was done to investigate the population structure of germplasm. Evanno’s method (Evanno et al., 2005) and cross-entropy method (Chan and Kroese, 2012) were used to obtain an optimum number of sub-populations. Filtered SNPs were used to calculate genetic distance among genotypes and the phylogenetic tree was constructed using neighbour-joining tree in TASSEL v5.0 (Bradbury et al., 2007) and visualized in iTOL v5 (Letunic and Bork, 2016).

### Genome-Wide Association Analysis

Filtered SNPs and BLUPs were used to perform association analysis using the Mixed Linear Model (MLM) (Zhang et al., 2010) and FarmCPU algorithms (Liu X. et al., 2016) with GAPIT v3 (Lipka et al., 2012) in R v4.0.3 (Core R Team 2019). The FarmCPU method was used to control false positives and false negatives by iteratively using a fixed-effect model (FEM) and random effect model (REM), testing marker associations from FEM as covariates in REM. *p*-value of 0.001 or -log_10_ *p*-value of 3.00 was used as a threshold to determine the significance of association (Ikram et al., 2020). The marker-trait associations (MTAs) identified for the same trait within a region of 100bp was considered as part of one QTL. The phenotypic variation explained (PVE) by each significant SNP was calculated as the squared correlation between the phenotype and genotype of the associated SNP (Bhandari et al., 2020). MTAs were considered stable QTLs if they were identified across all the environments of the respective locations with -log_10_ *p*-value ≥ 3 and PVEW ≥10%. *t*-test based determination of significance based on phenotypic data in two allelic groups was estimated at *p*-value ≤ 0.05 (Xiong et al., 2019).

### Postulation of Candidate Genes and KEGG Pathway Analysis

Candidate genes were postulated using the functional gene annotations of *Vigna radiata* reference genome (www.ncbi.nlm.nih.gov/assembly/GCF_000741045.1). A window of 200-kb region, upstream and downstream of the associated SNPs was searched to identify already reported candidate genes related to different traits studied (Park et al., 2019). Selected candidate genes were subjected to Kyoto Encyclopedia of Genes and Genomes (KEGG) analysis using Omics box 2.0.36 combined pathway analysis plugin.

## Results

### Phenotypic Evaluation

**FIGURE 1:**
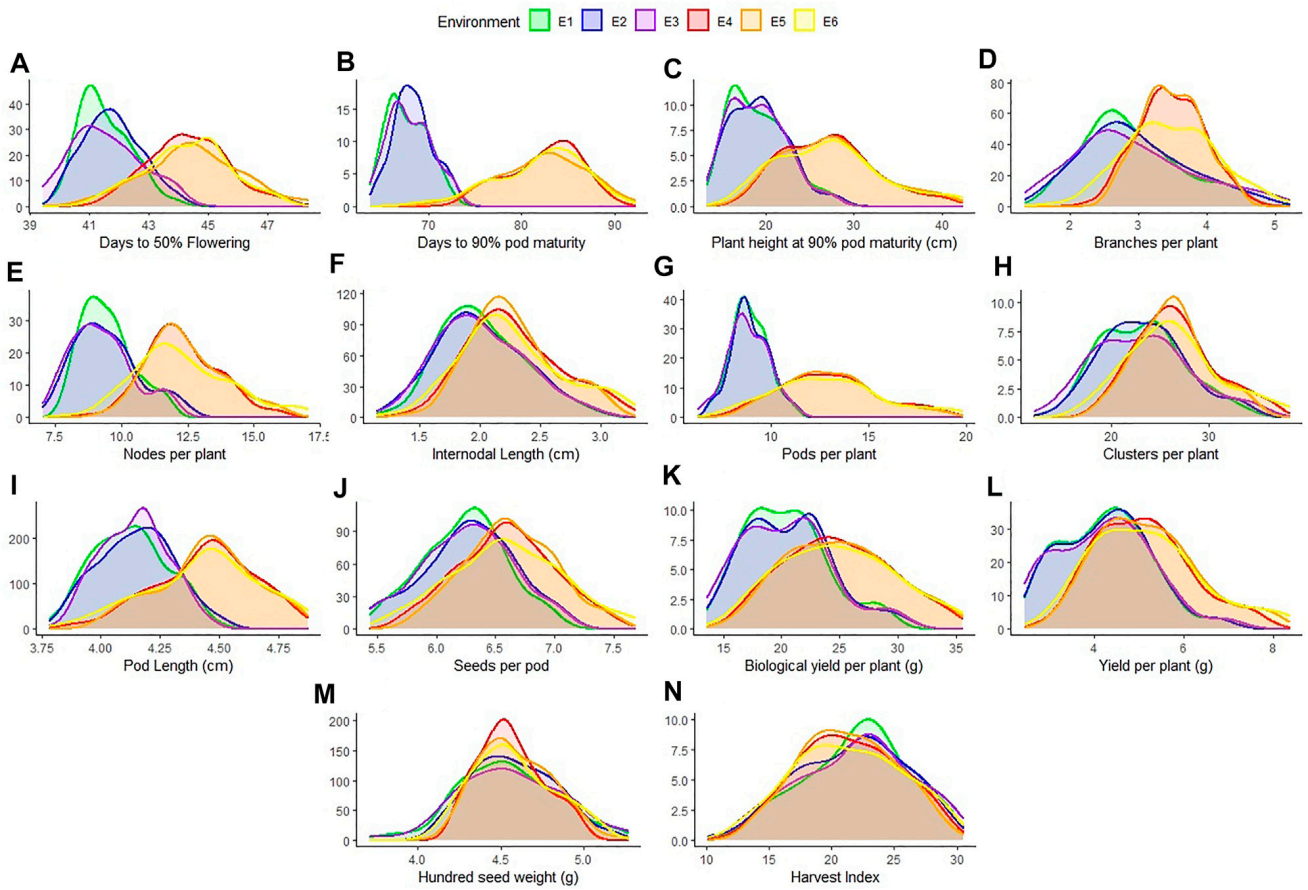
Distribution of 14 characters measured for 100 blackgram germplasm lines across all environments- Ludhiana 2019 (E1); Ludhiana 2020 (E2); Ludhiana combined across years (E3); Gurdaspur 2019 (E4); Gurdaspur 2020 (E5) and Gurdaspur combined across years (E6).

All the recorded traits showed high variability across the six given environments (Figure 1). All the traits followed normal distribution if considered in environments of each location however if compared across the two locations, the distribution of traits showed skewness. All the genotypes performed better in Gurdaspur than in Ludhiana. The negative skewness of trait data for the Gurdaspur location indicated an overall better performance of genotypes there, as compared to Ludhiana (Figure 1; Table 1). Since BLUPs accounted for the variation across the years for individual locations, the phenotypic evaluation is explained only for E3 and E6 to explain the overall variation at a particular location, for ease of understanding the variations (Table 1; Supplementary Table S2). DtM, and PL at Gurdaspur, and HI at Ludhiana were negatively skewed while all the other traits were either positively skewed or normally distributed (Figure 1). Genotypes sown in Gurdaspur showed delayed flowering (E6: 40.65–49.27 days) as compared to Ludhiana (E3: 39.42–47.08 days). The variation in DtM at Gurdaspur (E6: 68.88–90.73 days) were higher than at Ludhiana (E3:63.62–73.33 ± 2.3 days) with delayed maturation at Gurdaspur. The observed range of CpP and PpP was 5.59–17.08, and 12.07–40.43, respectively, at Ludhiana (E3) whereas 7.62–23.35, and 17–50.28, respectively, at Gurdaspur (E6), indicating better plant phenology at Gurdaspur. The range of PL was slightly more in Gurdaspur (E6: 03.85–05.16 cm) than Ludhiana (E3: 03.66–04.46 cm), eventually leading to higher SpP in Gurdaspur (E6: 05.63–07.68) than Ludhiana (E3: 05.45–07.08). Larger variability along with higher values were observed for BYpP at Gurdaspur (E6: 14.94–45.85 g) relative to Ludhiana (E3: 13.57–30.83 g). At Ludhiana (E3), YpP ranged from 2.45 to 8.44 g whereas it varied from 2.83 to 9.83 g at Gurdaspur (E6). HSW ranged from 3.71 to 5.26 g at Ludhiana (E3) and 4.12–5.33 g at Gurdaspur (E6). The five best performing genotypes representing best of all the traits at Ludhiana and Gurdaspur are presented in Supplementary Table S3.

**TABLE 1:** Phenotypic evaluation of 100 *V. mungo* genotypes for 14 traits recorded at two locations of Ludhiana and Gurdaspur as BLUPs of 2 years.

Trait	Env	Mean ± SD	Range	Geno.Sign	LSD	CV	H^2^
DtF	E3	41.81 ± 1.3	39.42–47.08	2E-28	01.97	03.61	0.85
	E6	44.57 ± 1.7	40.65–49.27	1E-26	01.81	02.64	0.91
DtM	E3	68.22 ± 2.3	63.62–73.33	2E-39	02.56	02.78	0.92
	E6	82.29 ± 4.7	68.88–90.73	1E-35	03.78	02.71	0.94
PHM	E3	20.93 ± 5.7	13.70–59.37	3E-82	02.85	09.77	0.99
	E6	29.90 ± 10.3	17.07–94.34	1E-73	05.66	12.60	0.98
BpP	E3	02.98 ± 0.9	01.33–05.05	2E-54	05.76	07.28	0.95
	E6	03.49 ± 0.7	01.82–05.86	2E-63	04.44	04.45	0.97
NpP	E3	09.84 ± 1.8	07.03–16.41	3E-41	01.59	12.00	0.92
	E6	12.74 ± 2.3	07.98–20.73	8E-43	02.14	11.57	0.93
IL	E3	02.18 ± 0.7	01.14–06.69	8E-71	00.37	12.19	0.98
	E6	02.35 ± 0.5	01.21–04.78	4E-47	00.45	13.37	0.94
CpP	E3	09.19 ± 1.7	05.59–17.08	2E-47	01.53	12.28	0.93
	E6	13.23 ± 3.2	07.62–23.35	6E-46	02.48	13.85	0.93
PpP	E3	23.89 ± 5.4	12.07–40.43	7E-46	04.80	14.45	0.93
	E6	27.43 ± 6.0	17.00–50.28	7E-46	04.93	13.32	0.92
PL	E3	04.19 ± 0.1	03.66–04.46	9E-16	00.48	05.02	0.81
	E6	04.53 ± 0.3	03.85–05.16	4E-55	00.32	04.94	0.95
SpP	E3	06.30 ± 0.4	05.45–07.08	3E-16	00.68	06.65	0.82
	E6	06.68 ± 0.5	05.63–07.68	6E-42	00.57	06.22	0.92
BYpP	E3	21.08 ± 4.0	13.57–30.83	4E-52	03.54	11.48	0.96
	E6	25.82 ± 5.3	14.94–45.85	2E-64	03.60	09.80	0.97
YpP	E3	04.45 ± 1.3	02.45–08.44	9E-62	00.72	11.45	0.96
	E6	05.28 ± 1.4	02.83–09.83	5E-57	00.82	11.35	0.96
HSW	E3	04.53 ± 0.3	03.71–05.26	5E-48	00.29	04.58	0.94
	E6	04.56 ± 0.3	04.12–05.33	4E-38	00.30	04.61	0.92
HI	E3	21.53 ± 4.6	09.49–30.55	2E-52	02.99	09.82	0.95
	E6	21.08 ± 4.3	11.92–29.62	4E-48	03.32	11.44	0.94

a SD -standard deviation, LSD- least significant difference, CV- coefficient of variation, H^2^—Broad sense heritability, Geno. Sign.-Genotype Significance.

b Ludhiana BLUPs, 2 years (E3); and Gurdaspur BLUPs, 2 years (E6).

c Days to 50% flowering (DtF); Days to 90% pod maturity (DtM); Plant height at 90% pod maturity (PHM); Branches per plant (BpP), Nodes per plant (NpP); Internodal length (IL), Clusters per plant (CpP); Pods per plant (PpP); Pod length (PL), Seeds per pod (SpP); Biological yield per plant (BYpP); Yield per plant (YpP); hundred seed weight (HSW) and Harvest index (HI).

Higher broad sense heritability (H^2^) for all traits under all environments suggested strong genetic control (Table 1). Highest H^2^ was observed for PHM (0.99 and 0.98), whereas lowest for DtF (0.61 and 0.91) both at Ludhiana and Gurdaspur. CpP (E3—0.93; E6—0.93), PpP (E3—0.93; E6—0.92), PL (E3—0.81; E6—0.95), SpP (E3—0.82; E6—0.92), BYpP (E3—0.96; E6—0.97), YpP (E3—0.96; E6—0.96), HSW (E3—0.94; E6—0.92) and HI (E3—0.95; E4 - 0.94) also recorded high H^2^.

### SNP Calling

**FIGURE 2:**
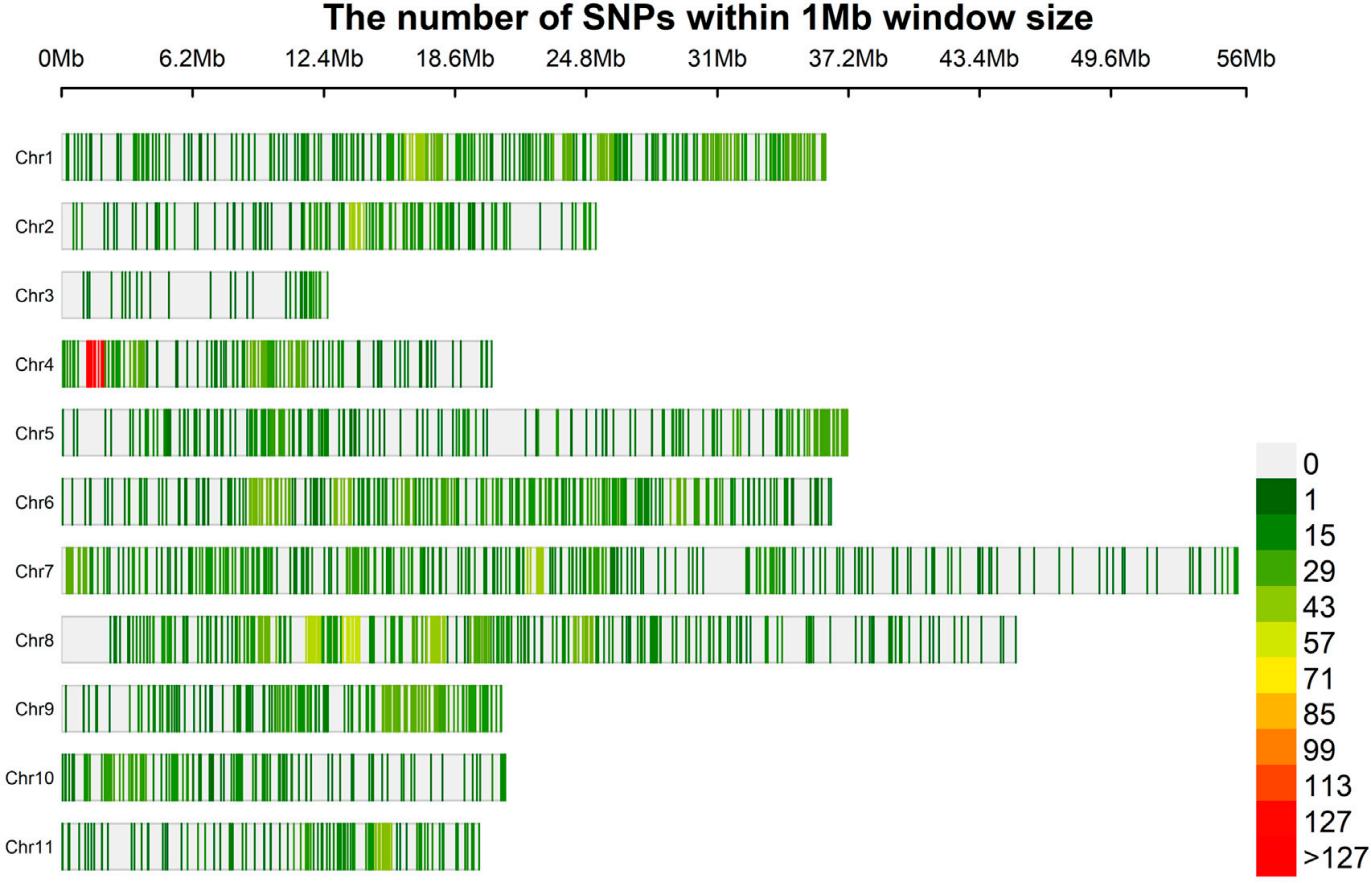
Physical map of 4,623 SNPs identified by GBS of 100 blackgram germplasm lines showing all the 11 chromosomes. Physical position is also shown in million of base pairs (Mb) based. SNP density is also provided in colours Dark Green (1) to Red (127) to reveal the distribution among chromosomes.

A total of 35, 49,948 raw physically mapped SNPs were obtained through GBS using the genome sequence of *Vigna radiata* as reference (Kang et al., 2014). Of these SNPs, 26, 39,464 were mapped onto 11 chromosomes while 9, 10,484 were mapped to non - chromosomal contigs. After filtering only 6,967 SNPs were retained of which 2,344 SNPs mapped to non-chromosomal contigs were removed and 4,623 on chromosomal regions used for further analysis (Supplementary Table S4, Figure 2). The highest density of SNPs was observed in Chr 4 (18.60 markers per Mb), whereas the lowest density was observed in Chr 3 (4.86) with an average density of 15.23 markers per Mb.

### Population Structure and Phylogenetic Analysis

**FIGURE 3:**
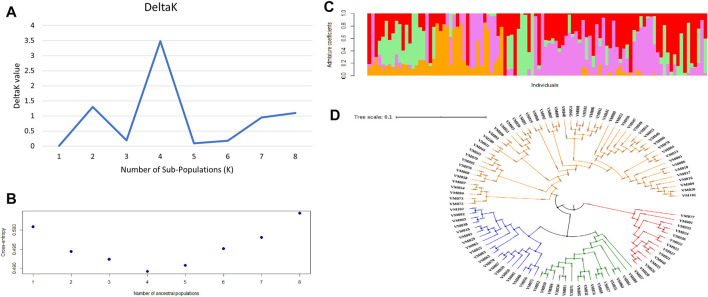
Population structure of 100 blackgram germplasm lines using 4623 SNP markers **(A)** determination of number of sub-populations by DeltaK method by Evanno et al., 2005 **(B)** determination of number of sub-populations using cross entropy value method by Chan and Kroese, 2012 **(C)** population structure at k = 4 **(D)** phylogenetic analysis of 100 blackgram germplasm lines depicting four sub-populations.

Four sub-populations (K = 4) were depicted by both the methods of second-order rate of change of the likelihood (Figure 3A) as well as cross-entropy value (Figure 3B). The graphical representation of four sub-populations against the admixture coefficient showed that more than 50% of the genotypes contributed to one sub population (Figure 3C). The allele frequency divergence was highest between sub-population two and four and the lowest between 3 and 4 (Supplementary Table S5). Average distances or expected heterozygosity of individuals within the same cluster were 0.3780 (sub-population 1), 0.2719 (sub-population 2), 0.0551 (sub-population 3) and 0.0250 (sub-population 4) (Supplementary Table S5). The mean Fst value of sub-populations 1, 2, 3 and four were 0.0203, 0.4537, 0.8040 and 0.9098, respectively (Supplementary Table S5). Multivariate analysis also classified the germplasm into four clusters affirming the results of structure analysis. The germplasm’s phylogenetic structure using an unweighted neighbour-joining tree showed the distribution of the different genotypes among the sub-populations (Figure 3D).

### Genome Wide Association Study

**FIGURE 4:**
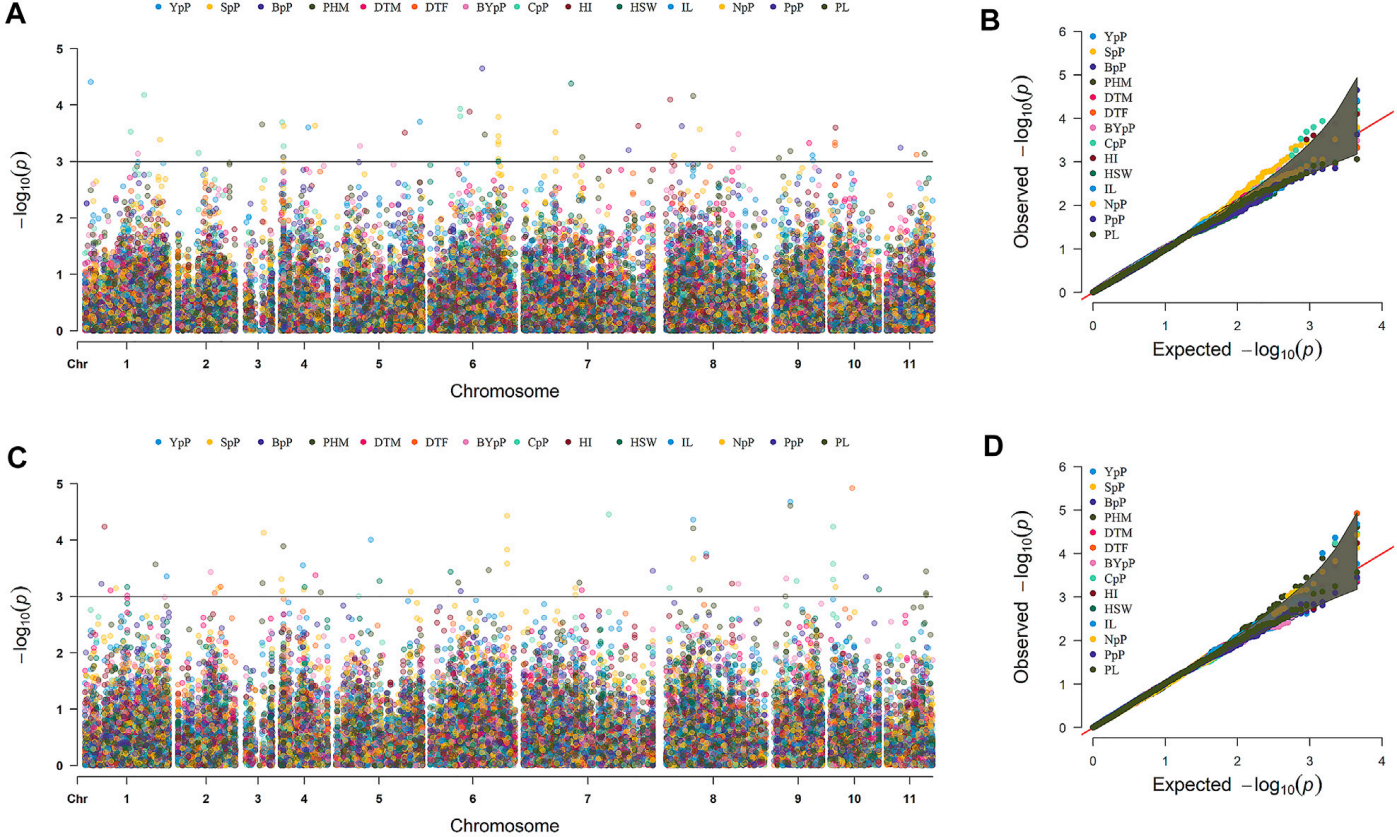
Marker trait associations for different traits detected by genome wide association study across two different locations Ludhiana **(A,B)** and Gurdaspur **(C,D)** as manhattan plots and QQ-plots using 4623 SNP markers. ^#^Days to 50% flowering (DtF); Days to 90% pod maturity (DtM); Plant height at 90% pod maturity (PHM); Nodes per plant (NpP); Internodal length (IL), Clusters per plant (CpP); Pods per plant (PpP); Seeds per pod (SpP); Biological yield per plant (BYpP); Yield per plant (YpP); hundred seed weight (HSW) and Harvest index (HI).

**FIGURE 5:**
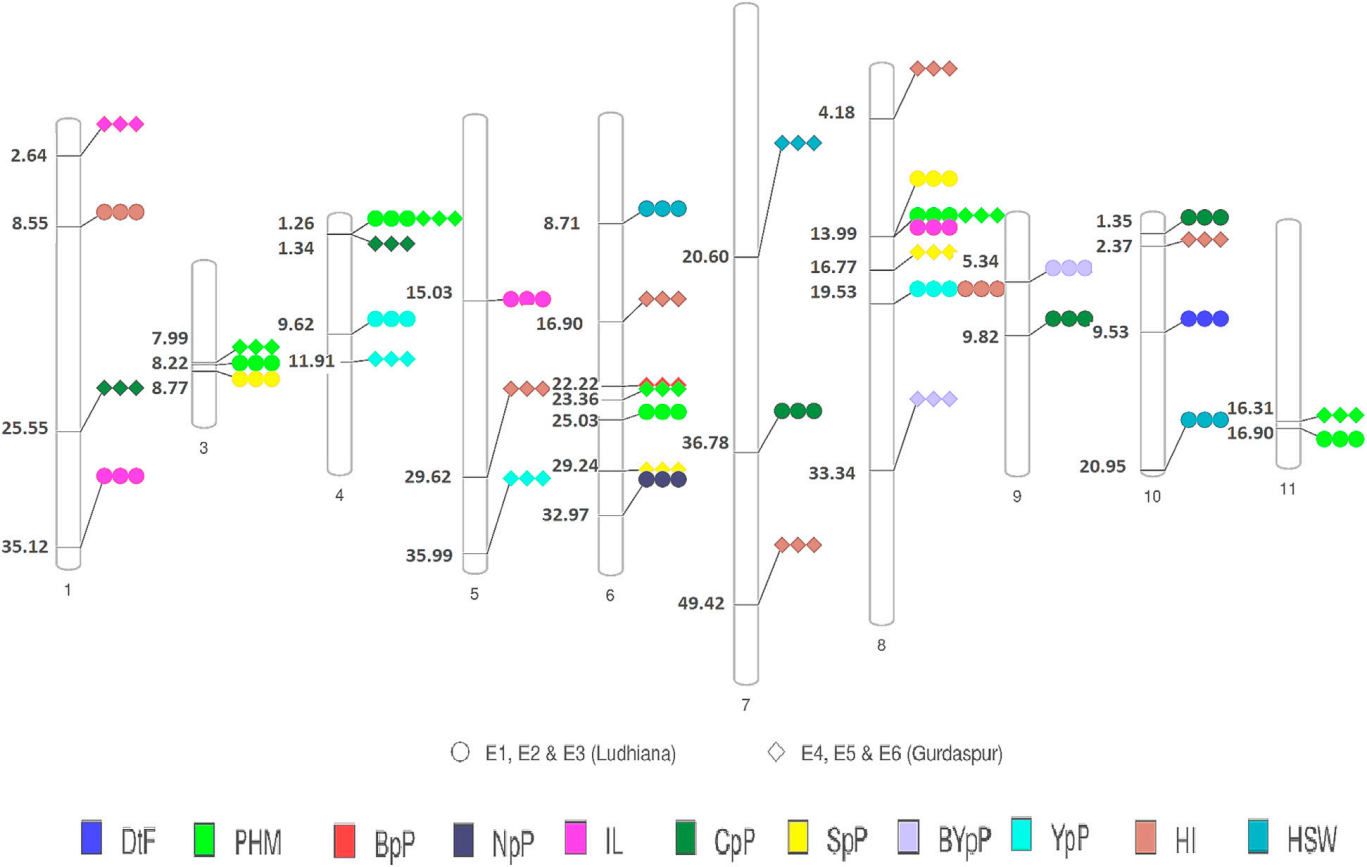
Physical map of QTLs of different traits detected by GWAS in the present study. Circles represent detection of across different environments at Ludhiana and diamonds represent detection of across different environments at Gurdaspur ^##^Days to 50% flowering (DtF); Days to 90% pod maturity (DtM); Plant height at 90% pod maturity (PHM); Nodes per plant (NpP); Internodal length (IL), Clusters per plant (CpP); Pods per plant (PpP); Seeds per pod (SpP); Biological yield per plant (BYpP); Yield per plant (YpP); hundred seed weight (HSW) and Harvest index (HI).

A total of 49 stable MTAs contributing to 42 QTLs were found to be significantly associated (-log_10_ *p*-value ≥ 3, PVE >10%) with 12 of the 14 traits studied, across the three environments of the each locations (Figure 4; Table 2; Supplementary Table S6). GWAS conducted using FarmCPU and MLM algorithms identified 31 and 27 QTLs, respectively of which 16 QTLs were common between two methods. However, only two MTAs *Q. PHM.4* & *Q. PHM.8* were significantly associated with PHM across both the locations. The negative log_10_ *p*-value of QTLs ranged from 3.009 to 5.112, whereas the PVE ranged from 10.06–24.26%. Among 42 QTLs identified in the study, four QTLs were associated with YpP, eight with PHM, one with BpP, two with NpP, four with IL, five with CpP, one with PL, four with SpP two with BYpP, seven with HI and three with HSW. However, no significant associations were obtained for DtM and PpP. SNPs S8.1.13991269 and S8.1.19533014, on chromosome 8, were found to be associated with two traits each i.e., PHM, IL (*Q. PHM.8* and *Q. IL.8*) and YpP, HI (*Q.YpP.8* and *Q. HI.8.2*), respectively (Figure 5).

**TABLE 2:** MTAs identified through Genome wide association study for 100 *Vigna mungo* germplasm lines in all the enviornments of two locations Ludhiana (L) or Gurdaspur (G) using FarmCPU and MLM algorithms.

MTA	Trait	Loc	Method	SNP	Chr	PosMb	-log_10_P	MAF	Effect	PVE
*Q.DtF.10*	DtF	L	FarmCPU	S10.1.9527186	10	9.5272	4.9208	0.07	1.68	17.947
	DtF	L	MLM	S10.1.9527186	10	9.5272	4.1457	0.07	1.5481	16.803
*Q.PHM.3.1*	PHM	G	FarmCPU	S3.1.7993147	3	7.9931	3.6566	0.115	-6.3321	16.506
*Q.PHM.3.2*	PHM	L	FarmCPU	S3.1.8219594	3	8.2196	3.2337	0.15	-3.0667	19.919
*Q.PHM.4*	PHM	L	FarmCPU	S4.1.1259800	4	1.2598	3.8912	0.065	7.009	17.084
	PHM	G	FarmCPU	S4.1.1259800	4	1.2598	3.0761	0.065	10.8299	16.46
	PHM	G	MLM	S4.1.1259800	4	1.2598	3.1226	0.065	11.6496	10.076
*Q.PHM.6.1*	PHM	G	FarmCPU	S6.1.23358875	6	23.3589	3.4745	0.105	8.5575	10.469
*Q.PHM.6.2*	PHM	L	FarmCPU	S6.1.25029431	6	25.0294	3.4664	0.205	-3.3382	14.698
*Q.PHM.8*	PHM	L	FarmCPU	S8.1.13991269	8	13.9913	4.2062	0.055	7.0402	17.697
	PHM	G	FarmCPU	S8.1.13991269	8	13.9913	4.1599	0.055	12.2652	11.234
	PHM	L	MLM	S8.1.13991269	8	13.9913	3.3945	0.055	6.216	10.728
	PHM	G	MLM	S8.1.13991269	8	13.9913	3.8356	0.055	11.9305	13.02
*Q.PHM.11.1*	PHM	G	FarmCPU	S11.1.16313748	11	16.3137	3.1353	0.19	5.1138	15.263
*Q.PHM.11.2*	PHM	L	FarmCPU	S11.1.16898133	11	16.8981	3.055	0.17	4.4754	15.99
	PHM	L	FarmCPU	S11.1.16898169	11	16.8982	3.0204	0.165	4.4335	16.003
	PHM	L	FarmCPU	S11.1.16898170	11	16.8982	3.0204	0.165	4.4335	16.003
	PHM	L	FarmCPU	S11.1.16898225	11	16.8982	3.4443	0.155	4.6729	15.565
*Q.BpP.6*	BpP	G	MLM	S6.1.22219189	6	22.2192	3.7615	0.19	-0.6003	14.341
*Q.NpP.4*	NpP	G	FarmCPU	S4.1.1343019	4	1.343	3.6303	0.065	2.2705	15.003
	NpP	G	MLM	S4.1.1343019	4	1.343	3.4822	0.065	2.2851	10.85
*Q.NpP.6*	NpP	L	FarmCPU	S6.1.32970231	6	32.9702	3.5832	0.095	1.7559	18.857
	NpP	L	FarmCPU	S6.1.32970252	6	32.9703	3.8288	0.055	2.267	19.048
	NpP	L	FarmCPU	S6.1.32970258	6	32.9703	4.4295	0.085	2.0396	22.292
	NpP	L	MLM	S6.1.32970258	6	32.9703	3.4366	0.085	1.8555	12.377
*Q.IL.1.1*	IL	G	FarmCPU	S1.1.2636313	1	2.6363	4.4078	0.115	0.4871	14.843
	IL	G	MLM	S1.1.2636313	1	2.6363	3.7944	0.115	0.4871	14.693
*Q.IL.1.2*	IL	L	FarmCPU	S1.1.35117546	1	35.1175	3.356	0.06	0.7035	14.089
*Q.IL.5*	IL	L	MLM	S5.1.15033908	5	15.0339	3.4791	0.08	0.8146	13.589
*Q.IL.8*	IL	L	FarmCPU	S8.1.13991269	8	13.9913	4.3615	0.055	0.8372	14.825
	IL	L	MLM	S8.1.13991269	8	13.9913	3.6602	0.055	0.8254	14.476
*Q.CpP.1*	CpP	G	FarmCPU	S1.1.25545554	1	25.5456	4.1785	0.1	-2.1507	24.26
*Q.CpP.4*	CpP	G	MLM	S4.1.774872	4	0.7749	3.5768	0.07	-3.1131	10.448
*Q.CpP.7*	CpP	L	FarmCPU	S7.1.36781633	7	36.7816	4.4572	0.06	1.9962	15.922
	CpP	L	MLM	S7.1.36781633	7	36.7816	3.8541	0.06	1.9962	14.712
*Q.CpP.9*	CpP	L	FarmCPU	S9.1.9823250	9	9.8233	3.2717	0.065	1.6571	14.218
*Q.CpP.10*	CpP	L	FarmCPU	S10.1.1346198	10	1.3462	3.5792	0.185	0.7477	12.487
	CpP	L	MLM	S10.1.1346198	10	1.3462	3.1982	0.185	0.7477	11.66
	CpP	L	FarmCPU	S10.1.1346205	10	1.3462	4.2381	0.06	1.9314	16.817
	CpP	L	MLM	S10.1.1346205	10	1.3462	3.6948	0.06	1.9314	13.959
	CpP	L	FarmCPU	S10.1.1346249	10	1.3462	3.3004	0.095	1.4055	12.019
*Q.PL.1*	PL	L	MLM	S1.1.30381382	1	30.3814	3.2522	0.075	-0.1458	11.747
*Q.SpP.3*	SpP	L	FarmCPU	S3.1.8767931	3	8.7679	4.1296	0.09	0.4397	15.931
	SpP	L	MLM	S3.1.8767931	3	8.7679	3.7625	0.09	0.4589	15.654
*Q.SpP.6*	SpP	G	MLM	S6.1.29235889	6	29.2359	3.501	0.13	-0.4234	14.263
*Q.SpP.8.1*	SpP	L	MLM	S8.1.13990816	8	13.9908	3.0808	0.165	-0.3413	12.201
*Q.SpP.8.2*	SpP	G	MLM	S8.1.16767849	8	16.7678	3.1283	0.145	0.3782	12.395
*Q.BYpP.8*	BYpP	G	FarmCPU	S8.1.33339056	8	33.3391	3.4848	0.15	3.7945	13.775
*Q.BYpP.9*	BYpP	L	FarmCPU	S9.1.5335251	9	5.3353	3.3148	0.175	-2.0795	13.007
*Q.YpP.4.1*	YpP	L	MLM	S4.1.9615705	4	9.6157	3.2364	0.07	1.3938	12.669
*Q.YpP.4.2*	YpP	G	FarmCPU	S4.1.11912711	4	11.9127	3.6025	0.095	1.1945	12.541
	YpP	G	MLM	S4.1.11912711	4	11.9127	3.4516	0.095	1.214	11.667
*Q.YpP.5*	YpP	G	FarmCPU	S5.1.35989113	5	35.9891	3.7002	0.08	-1.2605	11.018
	YpP	G	MLM	S5.1.35989113	5	35.9891	3.3001	0.08	-1.2365	11.032
*Q.YpP.8*	YpP	L	FarmCPU	S8.1.19533014	8	19.533	3.7596	0.17	-1.0298	10.751
	YpP	L	MLM	S8.1.19533014	8	19.533	3.3417	0.17	-1.0257	13.184
*Q.HI.1*	HI	L	FarmCPU	S1.1.8549788	1	8.5498	4.2388	0.095	-4.7838	11.425
	HI	L	MLM	S1.1.8549788	1	8.5498	3.8081	0.095	-4.8079	15.933
*Q.HI.5*	HI	G	FarmCPU	S5.1.29623709	5	29.6237	3.5094	0.145	-3.4666	11.234
*Q.HI.6*	HI	G	MLM	S6.1.16902154	6	16.9022	3.5694	0.08	4.5656	14.564
*Q.HI.7*	HI	G	FarmCPU	S7.1.49416375	7	49.4164	3.6277	0.16	-3.4401	18.045
*Q.HI.8.1*	HI	G	MLM	S8.1.4181215	8	4.1812	3.3758	0.095	4.5191	13.586
*Q.HI.8.2*	HI	L	FarmCPU	S8.1.19533014	8	19.533	3.7094	0.17	-3.5888	12.072
	HI	L	MLM	S8.1.19533014	8	19.533	3.2618	0.17	-3.5455	13.138
*Q.HI.10*	HI	G	MLM	S10.1.2365261	10	2.3653	3.1461	0.085	-4.2602	12.443
*Q.HSW.6*	HSW	L	FarmCPU	S6.1.8707985	6	8.708	3.4374	0.065	-0.3472	10.822
*Q.HSW.7*	HSW	G	FarmCPU	S7.1.20599131	7	20.5991	4.3788	0.08	0.2654	12.201
	HSW	G	MLM	S7.1.20599131	7	20.5991	3.5899	0.08	0.2455	12.339
*Q.HSW.10*	HSW	L	FarmCPU	S10.1.20952550	10	20.9526	3.1235	0.135	0.2536	11.731

a Marker trait associations (MTAs, Environment (Env), Chromosome (Chr), Position in million basepairs (PosMb), minor allele frequency (MAF), Phenotypic variation explained in percentage (PVE).

b Days to 50% flowering (DtF); Plant height at 90% pod maturity (PHM); Branches per plant (BpP), Nodes per plant (NpP); Internodal length (IL), Clusters per plant (CpP); Pod length (PL), Seeds per pod (SpP); Biological yield per plant (BYpP); Yield per plant (YpP); Harvest index (HI) and Hundred seed weight (HSW).

A few genomic regions harbored multiple significantly associated SNPs, as *Q. PHM.11.2* had four SNPs (-log_10_ *p*-value 3.0114–3.5826 and PVE 15.193–16.439%), *Q. NpP.6* (-log_10_ *p*-value 3.15–4.77, PVE 15.69–23.89) and *Q. CpP.10* both had three SNPs (-log_10_ *p*-value 3.11–4.66 and PVE 11.29–18.07%), whereas other QTLs were defined by a single SNP. Two QTLs for plant height at 90% pod maturity *Q. PHM.4* and *Q. PHM.8* showed consistent effect across all environments of both the locations. Among four QTLs identified for YpP, two (QTLs *Q. YpP.4.1* and *Q. YpP.8*) were detected at Ludhiana, whereas *Q. YpP.4.2* and *Q. YpP.5* were detected at Gurdaspur. Three QTLs for HSW were detected, two for Ludhiana and one for Gurdaspur (*Q.HSW.7*) which was detected by both the FarmCPU (-log_10_ *p*-value 4.38 and PVE 12.2%) and MLM (-log_10_ *p*-value 3.59 and PVE 12.34%) algorithms. Out of seven QTLs for HI, two were detected for Ludhiana (*Q. HI.1* and *Q. HI.8.2*) and five were detected for Gurdaspur (*Q. HI.5, Q. HI.6, Q. HI.7, Q. HI.8.1* and *Q. HI.10*). The phenotypic variation explained (PVE) by significant SNPs under FarmCPU method ranged from 10.751% (*Q.YpP.8*) to 22.292% (*Q.NpP.6*) at Ludhiana and 10.47% (*Q. PHM.6.1*) to 24.26% (*Q. CpP.1*) at Gurdaspur, whereas under MLM method, it ranged from 10.73% (*Q.SpP.8.1*) to 16.80% (*Q.PHM.8*) at Ludhiana and 10.08% (*Q.PHM.4*) to 14.69% (*Q.CpP.4*) at Gurdaspur.

### Allelic Effects

**FIGURE 6:**
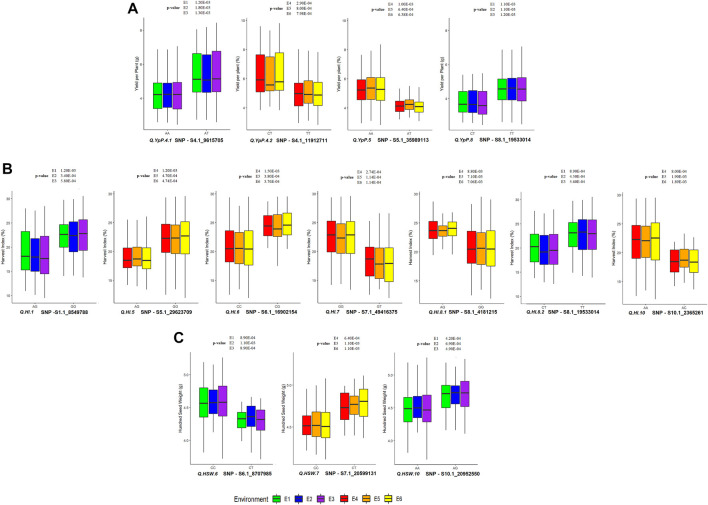
Allelic effect of QTLs associated with **(A)** YpP (Yield per Plant); **(B)** HI (Harvest Index) and **(C)** HSW (Hundred Seed Weight) at Ludhiana and Gurdaspur. *p*-value is provided from *t*-test for the respective environment.

Out of 49 SNPs/MTAs, 44 SNPs representing 37 QTLs showed significant difference, whereas five SNPs representing five QTLs were found to be non-significant (using the *t*-test statistic) for the respective traits (Supplementary Table S7, Supplementary Figures S2A–I. A total of 32, 32, 32, 28, 28 and 28 SNPs were statistically and significantly different for respective traits under E1, E2, E3, E4, E5 and E6, respectively. A total of 15 SNPs associated with 12 QTLs namely, *Q. IL.1.1*, *Q. IL.8*, *Q. NpP.6*, *Q. PHM.11.1*, *Q. PHM.11.2*, *Q. PHM.3.1*, *Q. PHM.3.2*, *Q. PHM.4*, *Q. PHM.6.1*, *Q. PHM.6.2*, *Q. PHM.8,* and *Q. SpP.8.2* were found to be significantly different in all of the six environments studied. The 28 SNP associations were significant in three of the six environments. The superior and inferior alleles along with allele count (%) and significantly different mean values observed at Ludhiana (E3) and Gurdaspur (E6) and are presented in Supplementary Table S8. The allele with SNP GG associated with *Q. SpP.8.2* was found to be superior at Ludhiana, whereas alternative allele was found to be superior at Gurdaspur. Allelic effects of YpP, HI and HSW are presented in Figure 6. The significant phenotypic differences produced by superior and inferior alleles for YpP were 1.3 g at Ludhiana (E3) by *Q. YpP.4.1*; 1.38 g at Gurdaspur (E6) by *Q. YpP.4.2*; 1.26 g at Gurdaspur (E6) by *Q. YpP.5* and 0.94 g at Ludhiana (E3) by *Q. YpP.8*; whereas significant phenotypic differences produced by superior and inferior alleles for HI were 3.91% at Ludhiana (E3) by *Q. HI.1*; 3.19% at Gurdaspur (E6) by *Q. HI.5*; 3.89% at Gurdaspur (E6) by *Q. HI.6*; 4.38% at Gurdaspur (E6) by *Q. HI.7.1*; 3.26% at Gurdaspur (E6) by *Q. HI.8.1*; 3.43% at Ludhiana (E3) by *Q. HI.8.2* and 3.66% at Gurdaspur (E6) by *Q. HI.10*. Likewise, significant phenotypic differences produced by superior and inferior alleles for HSW were 0.32 g at Ludhiana (E3) by *Q. HSW.6*, 0.25 g at Gurdaspur (E6) by *Q. HSW.7* and 0.24 g at Ludhiana (E3) by *Q. HSW.10*. A total of six genotypes (MASH218, IC274597, IC370938, IC557431, KUG673 and IC328783) were selected carrying superior alleles for all the traits under study and respective QTLs presented in Supplementary Table S9.

### Postulation of Candidate Genes

A total of 50 genes for 24 QTLs were identified with different functions for different traits, whereas no known gene was found for the remaining 18 QTLs (Table 3). Maximum genes with known function linked to the trait were identified for PHM 15) followed by CpP (9), YpP (7), SpP (5), IL (5), HI (5), HSW (3), and DtF (1), whereas no genes with known function for BpP, NpP, PL and BYpP were identified.

**TABLE 3:** List of candidate genes with their previously reported biological pathway function obtained in the putative QTL regions.

QTL	Trait	Gene ID	Dist (Kb)	Function
*Q.DtF.10*	DtF	*LOC106774489*	73.878	PHD finger-like domain-containing protein 5B
*Q.PHM.3.1*	PHM	*LOC106757287*	-100.237	E3 ubiquitin-protein ligase MARCH1
		*LOC106757069*	-65.973	bZIP transcription factor 53
		*LOC106757136*	-47.55	protein trichome birefringence-like 6
		*LOC106756978*	-43.026	histone-lysine N-methyltransferase EZ2-like
		*LOC111241394*	-33.113	DELLA protein RGL1-like
		*LOC106757804*	-2.328	DEAD-box ATP-dependent RNA helicase 24
		*LOC106757666*	50.731	probable WRKY transcription factor 23
		*LOC106756983*	98.775	gibberellin 2-beta-dioxygenase 2
		*LOC106756984*	118.512	transcription factor JAMYB-like
*Q.PHM.4*	PHM	*LOC106759452*	-178.201	tropinone reductase homolog
		*LOC106758588*	-69.349	tropinone reductase homolog At5g06060
*Q.PHM.6.2*	PHM	*LOC106764341*	-82.244	steroid 5-alpha-reductase DET2
*Q.PHM.11.1*	PHM	*LOC106777611*	-171.802	squamosa promoter-binding-like protein 14
		*LOC106777237*	-112.535	cytochrome P450 71D11
		*LOC106777539*	-82.108	pentatricopeptide repeat-containing protein At3g48810
*Q.IL.1.1*	IL	*LOC106766854*	-33.322	pectate lyase-like
		*LOC106765724*	-33.083	pectate lyase
*Q.IL.1.2*	IL	*LOC106762425*	-99.425	cytokinin hydroxylase
*Q.IL.5*	IL	*LOC106760883*	-189.1	purine permease 1
		*LOC106762422*	16.957	ethylene-responsive transcription factor RAP2-4
*Q.CpP.1*	CpP	*LOC106768944*	-108.532	SKP1-interacting partner 15
		*LOC106760064*	-32.774	receptor-like protein 12
		*LOC106760083*	68.491	polygalacturonase-like
*Q.CpP.7*	CpP	*LOC106765735*	-115.443	protein POLLEN DEFECTIVE IN GUIDANCE 1
		*LOC106766388*	42.507	LRR receptor-like serine/threonine-protein kinase RPK2
*Q.CpP.9*	CpP	*LOC111242573*	-79.009	eukaryotic translation initiation factor 3 subunit H-like
		*LOC106773784*	166.402	MLO-like protein 1
*Q.CpP.10*	CpP	*LOC106775061*	131.587	DDB1- and CUL4-associated factor 13
*Q.SpP.3*	SpP	*LOC106757271*	71.381	galactinol synthase 2
		*LOC106757661*	95.044	Golgi apparatus membrane protein-like protein ECHIDNA
		*LOC106756994*	100.043	alkaline/neutral invertase A, mitochondrial
*Q.SpP.6*	SpP	*LOC106765120*	64.962	dihydrofolate synthetase
*Q.SpP.8.2*	SpP	*LOC106770299*	147.684	ethylene-responsive transcription factor 1B-like
*Q.YpP.4.1*	YpP	*LOC106759105*	7.311	myb-related protein 305-like
*Q.YpP.5*	YpP	*LOC106761836*	-177.436	CLAVATA3/ESR (CLE)-related protein 5-like
		*LOC106760678*	-107.67	transcription factor PCF5
		*LOC106762074*	-50.026	sodium/calcium exchanger NCL
		*LOC106759995*	141.103	basic leucine zipper 34 isoform X1
*Q.YpP.8*	YpP	*LOC111242272*	116.438	alpha-mannosidase-like
		*LOC106771274*	129.392	putative 12-oxophytodienoate reductase 11
*Q.HI.1*	HI	*LOC106758323*	-174.544	UV-B-induced protein, chloroplastic isoform X1
*Q.HI.5*	HI	*LOC106760579*	-189.197	cytochrome P450 CYP72A219-like
*Q.HI.7*	HI	*LOC106769438*	105.344	protein root UVB sensitive 6
*Q.HI.8.2*	HI	*LOC111242272*	116.438	alpha-mannosidase-like
		*LOC106771274*	129.392	putative 12-oxophytodienoate reductase 11
*Q.HSW.6*	HSW	*LOC106764301*	-15.52	putative pentatricopeptide repeat-containing protein At1g12700, mitochondrial isoform X1
		*LOC106765194*	12.233	peroxidase 4
*Q.HSW.10*	HSW	*LOC106776199*	-175.528	bromodomain-containing protein 4B

a Distance of 5′ position of the gene from SNP, identified associated with the QTL, where–sign shows that the gene was located upstream of the SNP, and +sign shows that the gene was located downstream of the SNP.

b Days to 50% flowering (DtF); Plant height at 90% pod maturity (PHM); internodal length (IL), clusters per plant (CpP); seeds per pod (SpP); yield per plant (YpP); harvest index (HI) and hundred seed weight (HSW).

The gene *LOC106774489* was found 73 kb upstream of the *Q. DtF.10* coding PHD finger-like domain-containing enzyme 5B. Nine genes *LOC106757287* (-100.237 kb), *LOC106757069* (-65.973 kb), *LOC106757136* (-47.55 kb), *LOC106756978* (-43.026 kb), *LOC111241394* (-33.113 kb), *LOC106757804* (-2.328 kb), *LOC106757666* (50.731 kb), *LOC106756983* (98.775 kb), *LOC106756984* (118.512 kb) coding for already known enzyme E3 ubiquitin-enzyme ligase MARCH1, bZIP transcription factor 53, enzyme trichome birefringence-like 6, histone-lysine N-methyltransferase EZ2-like, DELLA enzyme RGL1-like, DEAD-box ATP-dependent RNA helicase 24, probable WRKY transcription factor 23, gibberellin 2-beta-dioxygenase two and transcription factor JAMYB-like for plant height were located near *Q. PHM.3.1*.

For internodal length, two genes *LOC106766854* (-33.322 kb) and *LOC106765724* (-33.083 kb), were found close to the *Q. IL.1.1* coding for pectate lyase-like and pectate lyase enzymes, respectively. Another gene *LOC106762425* (-99.425 kb-), with enzyme cytokinin hydroxylase, was found in proximity with *Q. IL.1.2*. The QTL *Q. IL.5* was near the genes *LOC106760883* (-189.1 kb) and *LOC106762422* (16.957 kb) coding for purine permease 1 and ethylene-responsive transcription factor RAP2-4 enzymes. For clusters per plant, three genes *LOC106768944*, *LOC106760064* and *LOC106760083* were in proximity of *Q. CpP.1* (-108.532kb, -32.774 and 68.491 kb) coding SKP1-interacting partner 15, receptor-like protein 12 and polygalacturonase-like enzyme, respectively.

For seeds per pod, three genes *LOC106757271* (71.381 kb), *LOC106757661* (95.044 kb) and *LOC106756994* (100.043 kb) were found close to the *Q. SpP.3* with enzyme galactinol synthase 2, Golgi apparatus membrane enzyme-like enzyme ECHIDNA and alkaline/neutral invertase A respectively. A gene *LOC106765120* (64.962 kb) with enzyme dihydrofolate synthetase, was found in the vicinity of *Q. SpP.6*. Another ethylene-responsive transcription factor 1B-like enzyme coding gene *LOC106770299* (147.684 kb) was found close to the *Q. SpP.8.2*. For yield per plant, a gene *LOC106759105* (7.311kb) with MYB-related protein 305-like enzyme was close to the QTL: *Q. YpP.4.1*. Four genes *LOC106761836* (-177.436 kb), *LOC106760678* (-107.67 kb), *LOC106762074* (-50.026 kb) and *LOC106759995* (141.103 kb) coding CLAVATA3/ESR (CLE)-related protein 5-like, transcription factor PCF5, sodium/calcium exchanger NCL and basic leucine zipper 34 isoform X1 enzymes were in vicinity of *Q. YpP.5*.

Two genes *LOC111242272* (116.438 kb) and *LOC106771274* (129.392 kb) with alpha-mannosidase-like and putative 12-oxophytodienoate reductase 11 enzymes was close to *Q. YpP.8*. For harvest index, gene *LOC106758323* (-174.544 kb) coding for UV-B-induced protein At3g17800, chloroplastic isoform X1 was related to *Q. HI.1*. The gene *LOC106760579* (-189.197 kb) was found in the proximity of *Q. HI.5* coding cytochrome P450 CYP72A219-like enzyme. The gene *LOC106769438* with protein root UVB sensitive six enzyme function was lying 105.344 kb of *Q. HI.7*. Two genes *LOC111242272* (116.438 kb) and *LOC106771274* (129.392 kb), with enzymes alpha-mannosidase-like and putative 12-oxophytodienoate reductase 11 respectively, were close to the *Q. HI.8.2*. Two genes *LOC106764301* (-15.52 kb) and *LOC106765194* (12.233 kb) with enzymes putative pentatricopeptide repeat-containing protein At1g12700 and peroxidase four, respectively, were close to the *Q. HSW.6*. The gene *LOC106776199* (-175.528 kb) encoding bromodomain-containing protein 4B enzyme was related to *Q. HSW.10*. The nodes per plant (NpP) had two QTLs, but no genes already known for nodes per plant around the vicinity of 200 kb of significant QTLs were found.

### KEGG Pathway Analysis

Some of the genomic regions significantly associated with the trait loci (Supplementary Table S10) such as Gibberellin 2-beta-dioxygenase (*LOC106756983*), cytochrome P450 CYP72A219 (*LOC106760579*) are involved in Diterpenoid biosynthesis (ko00904) pathway (Supplementary Figure S3). PHD finger-like domain-containing protein 5B (*LOC106774489*), and DEAD-box ATP-dependent RNA helicase 24 (*LOC106757804*) play role in Spliceosome (ko03040) process. Moreover, histone-lysine N-methyltransferase EZ2 found to be involved in Lysine degradation (Amino acid metabolism) ko0310. Besides that, pectate lyase-like (*LOC106766854*), pectate lyase (*LOC106765724*), and polygalacturonase-like (*LOC106760083*) have been found to play important role in Pentose and glucuronate interconversions (ko00040). LRR receptor-like serine/threonine-protein kinase RPK2 (*LOC106766388*), alkaline/neutral invertase A (*LOC106756994*) participates in Starch and sucrose metabolism (k000500). Galactinol synthase 2 (*LOC106757271*), alkaline/neutral invertase A (*LOC106756994*) have been shown to play role in Galactose metabolism (ko00052). Peroxidase 4 (*LOC106765194*) was found to play role in Phenylpropanoid biosynthesis (ko00940) (Supplementary Figure S4).

## Discussion

Blackgram is one of the most popular pulses in Southeast Asia, with India contributing 54% of world production (Singh et al., 2016). Inspite of having high nutritional value, short duration, and photo insensitivity the crop has not been exploited to its full potential. Multiple biotic and abiotic stresses and narrow genetic base of this crop is major hindrance in its expansion. Thus, a systematic approach is required to exploit untapped genetic diversity present within the country so that the germplasm could be exploited for improving breeding potential. . Present study is an effort to exploit the collection of diverse blackgram genotypes for important yield related component traits.

### Phenotypic Evaluation

Environment factors highly influence the complex traits; therefore, the material was replicated both in time and space. Previous studies have also reported a wide range of phenotypic variability for traits such as PHM, BpP, IL, CpP, PpP and HI (Panigrahi et al., 2014; Panda et al., 2017; Senthamizhselvi et al., 2019). High heritability was observed for all the traits indicating high genetic control and an effective phenotypic selection for these traits. Different studies have reported high broad-sense heritability for traits, i.e., DtF, DtM, PHM, CpP, PL and YpP (Panigrahi et al., 2014; Kumar et al., 2015); BYpP, HI and HSW (Rolaniya al., 2017; Kuralarasan et al., 2018). High heritability with high selection intensity helps breeders to shorten the breeding cycles of the program leading to faster and higher genetic gains. Many genotypes harbored combination of superior alleles for different yield related traits. For instance, the genotype IC274597 for CpP, PpP, SpP and BYpP; IC370938 for PpP, YpP, HSW and HI; KUG673 for DtF, DtM and HI; IC328783 for PHM, NpP and BYpP; These genotypes can be used in further breeding programs to improve desirable characters.

### Population Structure

Ad-hoc delta K and Cross entropy values suggested presence of four sub-populations in the blackgram germplasm which was further supported by phylogenetic analysis which in turn indicated significant diversity in the panel. High Fst values in 2, 3 and 4 sub-populations indicated them to be highly differentiated. A diverse germplasm panel can be a good source for a wide range of traits for breeding and research purpose (Govindaraj et al., 2015). Modelling of genetic structures as covariates helps in controlling the false positives while conducting GWAS. This is the first report on population structure analysis in blackgram; however, four sub-populations in mungbean germplasm have been reported earlier (Breria et al., 2020).

### Genome Wide Association Studies (GWAS)

GWAS for 14 yield associated traits identified 49 SNPs contributing to 42 QTLs to be strongly associated with 12 traits except DtM and PpP. Among 42 significant genomic regions identified in the study, the number of QTLs for each trait were as YpP (4), DtF (1), PHM (8), BpP (1), NpP (2), IL (4), CpP (5), PL (1), SpP (4), BYpP (2), HI (7) and HSW (3). High -log_10_ *p*-value and PVE suggested presence of major QTLs i.e. *Q. DtF.10*, *Q. PHM.3.2*, *Q. NpP.6*, *Q. IL.8*, *Q. CpP.10*, *Q. SpP.3*, *Q. BYpP.*9, *Q. YpP.8,* and *Q. HI.8.2* at Ludhiana, whereas *Q. PHM.3.1*, *Q. PHM.6.1*, *Q. NpP.4*, *Q. IL.1.1*, *Q. CpP.4*, *Q. SpP.6, Q. BYpP.8, Q. YpP.4.2,* and *Q. HI.6* at Gurdaspur were found significant for trait regulation. QTLs *Q. PHM.8,* and *Q. IL.8* were found to be located at same position on eighth chromosome controlling PHM and IL, respectively. The pleiotropy of increased PHM with increased IL has been earlier reported in faba bean (Hughes et al., 2020).

### Allelic Effects

The significant allelic effects of MTAs suggested selection of superior alleles could substantially lead to significant improvement of crop. At Ludhiana, QTL *Q. DtF.10* with superior allele TT resulted in earlier flowering with a mean of 41.42 days as compared to 43.05 with alternative allele. *Q. PHM.3.1* with superior allele AA recorded higher mean plant height of 28.54 cm, and 43.23 cm as compared to 19.21 cm, and 27.21 cm with alternative allele GG at Ludhiana, and Gurdaspur respectively. Superior allele TT of QTL *Q. IL.8* decreased internode length with mean value of 2.01 cm, whereas alternate allele observed higher internodal length with mean value of 2.83 cm. For clusters per plant, *Q. CpP.1* with superior allele AA exhibited mean of 18.84 clusters, whereas alternate allele TT exhibited mean of 12.73 clusters with The SNP S6.1.29235837 of *Q. SpP.6* having allele AA produced more average seeds per pod of 6.71 in contrast to 6.45 by alternate allele. Higher yield per plant with average of 5.47 g was observed by presence of AA allele of QTL *Q. YpP.5* while 4.21 g yield was observed with alternate allele. *Q. HI.1* with allele GG exhibited an increased mean value of harvest index (22.53%) as compared to a lower harvest index due to alternate allele (18.62%). *Q. HI.8.2* with allele TT showed a higher harvest index (22.87%) as compared to alternate allele (19.44%). Allelic effects with superior alleles and alternative alleles have been reported for significantly associated markers for root, nutrient uptake and yield related traits in rice (Subedi et al., 2019), for spike ethylene and spike dry weight in wheat (Valluru et al., 2017), and for yield related and heat tolernce traits in wheat (Dhillon et., 2021).

### Postulation of Genes

Blackgram is a highly self-pollinated crop and with the given narrow genetic base, is expected to have large LD blocks and large chunks of haplotypes being transferred over the generations without recombination. Keeping this in view, the SNPs identified for traits can be searched upstream and downstream for candidate genes governing those traits (Dhillon et al., 2020). A total of 50 genes for 24 QTLs were identified with different functions with respect to different traits. The QTL *Q. DtF.10* identified for days to flowering was present in the vicinity of the gene coding plant homeodomain finger-like domain-containing enzyme 5B can be a putative gene as PHD finger-like genes are reported to delay flowering Arabidopsis (Greb et al., 2007).

The enzymes encoded by genes found for QTLs of PHM were involved in controlling the plant height as supported by earlier studies of enzyme E3 ubiquitin-enzyme ligase MARCH1 in rice (Hu et al., 2013), bZIP transcription factor 53 in soybean (Ali et al., 2016), enzyme trichome birefringence-like six in Rice (Gao et al., 2017), histone-lysine N-methyltransferase EZ2-like (https://www.uniprot.org/uniprot/O23372), DELLA enzyme RGL1-like in Arabidopsis (Serrano-Mislata et al., 2017), DEAD-box ATP-dependent RNA helicase 24 in rice (Wang et al., 2016), probable WRKY transcription factor 23 in rice (Cai et al., 2014), gibberellin 2-beta-dioxygenase two in Arabidopsis (Rosin et al., 2003), transcription factor JAMYB-like in rice (Zhang et al., 2017), acyl transferase four in rice (Basu et al., 2017), tropinone reductase homolog (Stead, 1989), in grapevine (Guillaumie et al., 2020), steroid 5-alpha-reductase DET2 in soybean (Huo et al., 2018), squamosa promoter-binding-like enzyme 14 in rice (Lu et al., 2013), cytochrome P450 71D11 in cucumber (Wang et al., 2017), pentatricopeptide repeat-containing enzyme At3g48810 in Arabidopsis (Lee et al., 2019), and pentatricopeptide repeat-containing enzyme At1g31430 in Arabidopsis (Lee et al., 2019).

For internodal length, genes coding for pectate lyase-like (PLL), and pectate lyase (PL) in rice (Leng et al., 2017), cytokinin hydroxylase in Arabidopsis (Kiba et al., 2013), purine permease 1 in cotton (Wang et al., 2020), ethylene-responsive transcription factor RAP2-4 enzymes Arabidopsis (Hinz et al., 2010) have been earlier reported. For clusters per plant, genes SKP1-interacting partner 15 (Lu et al., 2016), receptor-like protein 12 (Wang et al., 2008), polygalacturonase-like enzyme (Xiao et al., 2014) in Arabidopsis, probable trehalose-phosphate phosphatase C isoform X2 in *Nelumbo nucifera* (Jin et al., 2016), lysine-specific demethylase JMJ25 isoform X1 in Arabidopsis (Jiang et al., 2007), pollen defective in guidance 1 (Li et al., 2011), LRR receptor-like serine/threonine-protein kinase RPK2 enzymes in Arabidopsis (Mizuno et al., 2007), eukaryotic translation initiation factor 3 subunit H-like in Arabidopsis (Zhou et al., 2014), MLO-like protein 1 reported in peach (Ruperti et al., 2002), and DDB1- and CUL4-associated factor 13 enzyme (Bjerkan and Grini, 2013) has been earlier reported to be involved in controlling cell elongation and flower development.

For seeds per pod, galactinol synthase two in Chickpea (Salvi et al., 2016), Golgi apparatus membrane enzyme-like enzyme ECHIDNA in Arabidopsis (Jia et al., 2018), 60S ribosomal protein L28-2 in soybean (Jones et al., 2020), dihydrofolate synthetase in Arabidopsis (Corral et al., 2018), and ethylene-responsive transcription factor 1B-like enzyme in brassica (Kaur et al., 2020) were known to have a role in increasing number of seeds per pod/siliqua. For yield per plant, myb-related protein 305-like in Arabidopsis (Ambawat et al., 2013), CLAVATA3/ESR (CLE)-related protein 5-like in Arabidopsis (Fletcher, 2018), transcription factor PCF5 in wheat (Zhao et al., 2018), sodium/calcium exchanger NCL in soybean (Liu Y. et al., 2016), basic leucine zipper 34 isoform X1 enzymes in wheat (Sornaraj et al., 2016), alpha-mannosidase-like in wheat (Dal Cortivo et al., 2020), and putative 12-oxophytodienoate reductase 11 in wheat (Pigolev et al., 2018) were previously known for yield improvement. For harvest index, UV-B-induced protein At3g17800 in basil (Dou et al., 2019), cytochrome P450 CYP72A219-like enzyme in Arabidopsis (Bak et al., 2011), root UV-B sensitive in wheat (Agrawal et al., 2004), alpha-mannosidase-like in wheat (Dal Cortivo et al., 2020), and putative 12-oxophytodienoate reductase 11 in wheat (Pigolev et al., 2018) are known for triggering reductions in biomass, yield and harvest index.

The genes detected for HSW, pentatricopeptide repeat-containing protein At1g80550, have been earlier reported for kernel development in maize (Dai et al., 2018; Ren et al., 2019); peroxidase four in soybean (Zhang et al., 2015). The clusters per plant had several QTLs, but no known genes were identified in 200 kb genomic region.

### KEGG Pathway Analysis

KEGG analysis revealed role of candidate genes in biological pathways related to respective traits. Gibberellins (GAs) are endogenous phytohormones that are involved in the regulation of the life cycle of plants. It has been identified that the locus encoding gibberellin 2-beta-dioxygenase/GA 2-oxidase present in vicinity of SNP S3.1.7993147 on chromosome 3 significantly associated with plant height at 90% pod maturity participates in Diterpenoid biosynthesis (ko00904) pathway. The role of this locus in the regulation of plant growth in rice has been demonstrated by Sakamoto et al., 2001. KEGG pathway established role of another gene coding for steroid 5-alpha-reductase DET2 with QTL associated with PHM on chromosome six to participate in Brassinosteroid biosynthesis. Ortholog of this gene in cotton (*Gossypium hirsutum* L.), GhDET2 along with BRs are known to play a crucial role in the initiation and elongation of cotton fiber cells (Luo et al., 2007). Similarly, DET2 steroid 5d-reductase in Arabidopsis catalyzes a major rate-limiting in brassinosteroid (BR) biosynthesis (Nakaya et al., 2002). Additionally, Cytochrome P450 CYP72A219-like has also been reported to participate in Diterpenoid biosynthesis pathway (Bathe and Tissier, 2019). Using KEGG pathway analysis, function of only two QTLs could be established with diterpenoid pathway and brassinosteroid biosynthesis pathway. However, the function of remaining significant genomic regions could not be established using this analysis.

## Conclusion

GWAS analysis led to identification of novel MTA and few putative candidate genes. Though candidate genes need to be examined further and detailed investigations would validate their roles in governing agronomically important traits but the MTA will really help in selecting the genotypes with positive associations. The information derived from this study can be used in the generation of SNP based molecular markers to select traits of interest and accelerate blackgram breeding programme with a better ideotype. Since the blackgram is neglected crop in term of number of genetic and genomic resources, the current study, first of its kind in this crop will definitely open new avenues for broadening its base.

## Data Availability

The datasets presented in this study can be found in online repositories. The names of the repository/repositories and accession number(s) can be found below: NCBI Bioproject, accession number PRJNA802066.
