# Supplementary material for: Genome-wide Association Study for Yield and Yield-Related Traits in Diverse Blackgram Panel (Vigna mungo L. Hepper) Reveals Novel Putative Alleles for Future Breeding Programs
Source: Front Genet. 2022 Jul 11;13:849016. doi: 10.3389/fgene.2022.849016 (PMC9310006; doi:10.3389/fgene.2022.849016)
Supplement: Supplementary file 1 [file Table1.docx]

**Table S1: List of *Vigna mungo* genotypes used in the present investigation with their source**

| S. No. | Genotype | Source | S. No. | Genotype | Source | S. No | Genotype | Source |
| --- | --- | --- | --- | --- | --- | --- | --- | --- |
| 1 | IC140817 | NBPGR | 35 | IC472000 | NBPGR | 69 | PU15-38 | GBPUAT |
| 2 | IC144901 | NBPGR | 36 | IC519836 | NBPGR | 70 | PU15-44 | GBPUAT |
| 3 | IC257654 | NBPGR | 37 | IC519918 | NBPGR | 71 | PU31 | GBPUAT |
| 4 | IC257676 | NBPGR | 38 | IC530476 | NBPGR | 72 | PU19 | GBPUAT |
| 5 | IC261171 | NBPGR | 39 | IC530481 | NBPGR | 73 | IPU2-33 | IIPR KANPUR |
| 6 | IC269077 | NBPGR | 40 | IC530607 | NBPGR | 74 | IPU2-43 | IIPR KANPUR |
| 7 | IC274597 | NBPGR | 41 | IC530624 | NBPGR | 75 | IPU94-2 | IIPR KANPUR |
| 8 | IC279538 | NBPGR | 42 | IC530625 | NBPGR | 76 | RUG46 | IIPR, KANPUR |
| 9 | IC281997 | NBPGR | 43 | IC530630 | NBPGR | 77 | TV-99-2 | IIPR, KANPUR |
| 10 | IC296661 | NBPGR | 44 | IC557431 | NBPGR | 78 | PDU1 | KANPUR |
| 11 | IC305227 | NBPGR | 45 | IC557433 | NBPGR | 79 | NDU2-13-1 | NDU, AYODHYA |
| 12 | IC305283 | NBPGR | 46 | IC569464 | NBPGR | 80 | PATHANKOT | PAU |
| 13 | IC316310 | NBPGR | 47 | IC570267 | NBPGR | 81 | KUG363 | PAU |
| 14 | IC321482 | NBPGR | 48 | IC589045 | NBPGR | 82 | KUG489 | PAU |
| 15 | IC321654 | NBPGR | 49 | IC611676 | NBPGR | 83 | KUG503 | PAU |
| 16 | IC328783 | NBPGR | 50 | IC616484 | NBPGR | 84 | KUG586 | PAU |
| 17 | IC330880 | NBPGR | 51 | KARS-88 | NBPGR | 85 | KUG662 | PAU |
| 18 | IC330901 | NBPGR | 52 | OBG35 | ANGRAU | 86 | KUG673 | PAU |
| 19 | IC330912 | NBPGR | 53 | OBG36 | ANGRAU | 87 | KUG678 | PAU |
| 20 | IC331226 | NBPGR | 54 | LBG787 | ANGRAU | 88 | KUG744 | PAU |
| 21 | IC370938 | NBPGR | 55 | LBG788 | ANGRAU | 89 | KUG96-3 | PAU |
| 22 | IC390026 | NBPGR | 56 | DKU4 | ANGRAU | 90 | Mash1008 | PAU |
| 23 | IC393553 | NBPGR | 57 | KPU405 | ARS, Kota | 91 | Mash1-1 | PAU |
| 24 | IC395519 | NBPGR | 58 | PU6 | GBPUAT | 92 | Mash114 | PAU |
| 25 | IC396727 | NBPGR | 59 | UTTRA | GBPUAT | 93 | Mash218 | PAU |
| 26 | IC396752 | NBPGR | 60 | PantU19 | GBPUAT | 94 | Mash338 | PAU |
| 27 | IC398148 | NBPGR | 61 | PantU30 | GBPUAT | 95 | Mash391 | PAU |
| 28 | IC399642 | NBPGR | 62 | PU11-14 | GBPUAT | 96 | Mash479 | PAU |
| 29 | IC422061 | NBPGR | 63 | PU11-25 | GBPUAT | 97 | T9 | PAU |
| 30 | IC427979 | NBPGR | 64 | PU15-26 | GBPUAT | 98 | V.sylvestris | PAU |
| 31 | IC427980 | NBPGR | 65 | PU15-27 | GBPUAT | 99 | VBN6 | TNAU |
| 32 | IC436560 | NBPGR | 66 | PU15-28 | GBPUAT | 100 | VERBEN7 | TNAU |
| 33 | IC449252 | NBPGR | 67 | PU15-30 | GBPUAT |  |  |  |
| 34 | IC471986 | NBPGR | 68 | PU15-36 | GBPUAT |  |  |  |

**Table S2: Phenotypic evaluation of** 1**00 *V. mungo* germplasm lines for 14 traits recorded for two locations Ludhiana and Gurdaspur across two years**

| **Statistic** | **Env** | **Mean±SD** | **Range** | **Geno. Sig.** | **LSD** | **CV** | **H^2^** |
| --- | --- | --- | --- | --- | --- | --- | --- |
| DtF | E1 | 41.67±0.9 | 39.72-45.22 | 3.00E-06 | 02.67 | 04.11 | 0.61 |
|  | E2 | 41.95±1.1 | 40.08-46.08 | 1.00E-06 | 02.80 | 04.23 | 0.63 |
|  | E3 | 41.81±1.3 | 39.42-47.08 | 2.00E-28 | 01.97 | 03.61 | 0.85 |
|  | E4 | 44.48±1.4 | 41.51-48.44 | 5.00E-10 | 02.51 | 03.01 | 0.79 |
|  | E5 | 44.66±1.9 | 40.32-49.37 | 7.00E-24 | 02.04 | 02.24 | 0.90 |
|  | E6 | 44.57±1.7 | 40.65-49.27 | 1.00E-26 | 01.81 | 02.64 | 0.91 |
| DtM | E1 | 67.92±2.2 | 63.77-73.05 | 4.00E-18 | 03.59 | 02.89 | 0.84 |
|  | E2 | 68.51±2.1 | 64.18-73.24 | 1.00E-13 | 03.97 | 03.22 | 0.80 |
|  | E3 | 68.22±2.3 | 63.62-73.33 | 2.00E-39 | 02.56 | 02.78 | 0.92 |
|  | E4 | 82.30±4.4 | 69.31-89.77 | 6.00E-20 | 05.12 | 03.12 | 0.88 |
|  | E5 | 82.29±5.0 | 65.81-92.22 | 3.00E-35 | 03.81 | 02.24 | 0.95 |
|  | E6 | 82.29±4.7 | 68.88-90.73 | 1.00E-35 | 03.78 | 02.71 | 0.94 |
| PHM | E1 | 20.89±5.8 | 13.47-61.06 | 6.00E-55 | 04.44 | 10.75 | 0.98 |
|  | E2 | 20.97±5.6 | 13.31-56.67 | 4.00E-50 | 04.57 | 10.88 | 0.97 |
|  | E3 | 20.93±5.7 | 13.70-59.37 | 3.00E-82 | 02.85 | 09.77 | 0.99 |
|  | E4 | 29.83±9.4 | 18.27-88.68 | 2.00E-30 | 10.03 | 17.08 | 0.93 |
|  | E5 | 29.96±9.7 | 18.04-92.22 | 7.00E-35 | 09.06 | 15.09 | 0.94 |
|  | E6 | 29.90±10.3 | 17.07-94.34 | 1.00E-73 | 05.66 | 12.60 | 0.98 |
| BpP | E1 | 02.90±0.7 | 01.61-04.70 | 7.00E-12 | 01.04 | 19.75 | 0.79 |
|  | E2 | 02.95±0.8 | 01.43-04.83 | 6.00E-16 | 00.95 | 16.98 | 0.84 |
|  | E3 | 02.98±0.9 | 01.33-05.05 | 2.00E-54 | 05.76 | 07.28 | 0.95 |
|  | E4 | 03.51±0.5 | 02.30-05.28 | 2.00E-06 | 01.08 | 18.93 | 0.65 |
|  | E5 | 03.52±0.5 | 02.31-05.05 | 5.00E-06 | 01.10 | 19.47 | 0.63 |
|  | E6 | 03.49±0.7 | 01.82-05.86 | 2.00E-63 | 04.44 | 04.45 | 0.97 |
| NpP | E1 | 09.77±1.4 | 07.73-14.73 | 4.00E-09 | 02.53 | 15.12 | 0.72 |
|  | E2 | 09.90±1.8 | 07.04-16.31 | 4.00E-17 | 02.29 | 12.47 | 0.84 |
|  | E3 | 09.84±1.8 | 07.03-16.41 | 3.00E-41 | 01.59 | 12.00 | 0.92 |
|  | E4 | 12.74±1.9 | 08.77-19.30 | 9.00E-12 | 03.29 | 14.13 | 0.78 |
|  | E5 | 12.75±1.8 | 09.26-19.12 | 1.00E-09 | 03.37 | 14.92 | 0.74 |
|  | E6 | 12.74±2.3 | 07.98-20.73 | 8.00E-43 | 02.14 | 11.57 | 0.93 |
| IL | E1 | 02.18±0.6 | 01.20-06.43 | 2.00E-30 | 00.67 | 16.09 | 0.92 |
|  | E2 | 02.17±0.6 | 01.17-06.55 | 1.00E-38 | 00.54 | 12.99 | 0.95 |
|  | E3 | 02.18±0.7 | 01.14-06.69 | 8.00E-71 | 00.37 | 12.19 | 0.98 |
|  | E4 | 02.34±0.4 | 01.42-04.27 | 3.00E-15 | 00.69 | 15.84 | 0.82 |
|  | E5 | 02.36±0.4 | 01.42-04.62 | 2.00E-14 | 00.73 | 16.92 | 0.81 |
|  | E6 | 02.35±0.5 | 01.21-04.78 | 4.00E-47 | 00.45 | 13.37 | 0.94 |
| CpP | E1 | 09.11±1.3 | 06.11-15.62 | 4.00E-12 | 02.64 | 16.72 | 0.76 |
|  | E2 | 09.26±1.6 | 06.01-16.48 | 5.00E-21 | 02.12 | 12.37 | 0.86 |
|  | E3 | 09.19±1.7 | 05.59-17.08 | 2.00E-47 | 01.53 | 12.28 | 0.93 |
|  | E4 | 13.21±2.8 | 08.28-21.77 | 9.00E-16 | 03.74 | 15.79 | 0.82 |
|  | E5 | 13.24±2.8 | 08.53-22.14 | 2.00E-13 | 04.02 | 17.20 | 0.79 |
|  | E6 | 13.23±3.2 | 07.62-23.35 | 6.00E-46 | 02.48 | 13.85 | 0.93 |
| PpP | E1 | 23.80±4.6 | 13.73-38.15 | 2.00E-14 | 07.40 | 17.22 | 0.80 |
|  | E2 | 23.99±4.6 | 13.84-37.81 | 4.00E-13 | 07.72 | 18.06 | 0.78 |
|  | E3 | 23.89±5.4 | 12.07-40.43 | 7.00E-46 | 04.80 | 14.45 | 0.93 |
|  | E4 | 27.37±5.0 | 18.69-46.15 | 6.00E-13 | 07.75 | 16.20 | 0.77 |
|  | E5 | 27.50±5.0 | 18.66-47.07 | 4.00E-13 | 07.82 | 16.26 | 0.78 |
|  | E6 | 27.43±6.0 | 17.00-50.28 | 7.00E-46 | 04.93 | 13.32 | 0.92 |
| PL | E1 | 04.16±0.2 | 03.65-04.49 | 9.00E-24 | 00.37 | 04.61 | 0.89 |
|  | E2 | 04.22±0.2 | 03.56-04.51 | 8.00E-27 | 00.44 | 05.33 | 0.91 |
|  | E3 | 04.19±0.1 | 03.66-04.46 | 9.00E-16 | 00.48 | 05.02 | 0.81 |
|  | E4 | 04.53±0.2 | 03.90-05.11 | 3.00E-21 | 00.51 | 05.92 | 0.87 |
|  | E5 | 04.53±0.2 | 03.94-05.11 | 3.00E-18 | 00.53 | 06.27 | 0.85 |
|  | E6 | 04.53±0.3 | 03.85-05.16 | 4.00E-55 | 00.32 | 04.94 | 0.95 |
| SpP | E1 | 06.25±0.4 | 05.19-07.02 | 2.00E-11 | 00.71 | 06.35 | 0.77 |
|  | E2 | 06.35±0.4 | 05.08-07.05 | 2.00E-17 | 00.79 | 06.68 | 0.84 |
|  | E3 | 06.30±0.4 | 05.45-07.08 | 3.00E-16 | 00.68 | 06.65 | 0.82 |
|  | E4 | 06.67±0.4 | 05.73-07.48 | 9.00E-15 | 00.85 | 07.01 | 0.81 |
|  | E5 | 06.68±0.4 | 05.81-07.49 | 1.00E-09 | 00.90 | 07.87 | 0.72 |
|  | E6 | 06.68±0.5 | 05.63-07.68 | 6.00E-42 | 00.57 | 06.22 | 0.92 |
| BYpP | E1 | 20.89±3.5 | 14.49-28.96 | 1.00E-17 | 05.96 | 14.96 | 0.86 |
|  | E2 | 21.27±3.8 | 13.88-30.89 | 3.00E-23 | 05.18 | 12.30 | 0.90 |
|  | E3 | 21.08±4.0 | 13.57-30.83 | 4.00E-52 | 03.54 | 11.48 | 0.96 |
|  | E4 | 25.74±4.9 | 15.62-43.54 | 8.00E-28 | 05.81 | 11.86 | 0.91 |
|  | E5 | 25.91±5.0 | 15.65-44.82 | 1.00E-26 | 06.09 | 12.45 | 0.90 |
|  | E6 | 25.82±5.3 | 14.94-45.85 | 2.00E-64 | 03.60 | 09.80 | 0.97 |
| YpP | E1 | 04.44±1.2 | 02.59-08.06 | 4.00E-24 | 01.21 | 14.42 | 0.89 |
|  | E2 | 04.45±1.2 | 02.57-08.19 | 2.00E-25 | 01.16 | 13.80 | 0.90 |
|  | E3 | 04.45±1.3 | 02.45-08.44 | 9.00E-62 | 00.72 | 11.45 | 0.96 |
|  | E4 | 05.28±1.3 | 02.94-09.59 | 7.00E-24 | 01.27 | 12.82 | 0.89 |
|  | E5 | 05.28±1.2 | 03.12-09.30 | 4.00E-19 | 01.39 | 14.30 | 0.86 |
|  | E6 | 05.28±1.4 | 02.83-09.83 | 5.00E-57 | 00.82 | 11.35 | 0.96 |
| HSW | E1 | 04.52±0.3 | 03.82-05.19 | 2.00E-19 | 00.40 | 04.86 | 0.86 |
|  | E2 | 04.54±0.3 | 03.82-05.15 | 2.00E-13 | 00.48 | 05.98 | 0.78 |
|  | E3 | 04.53±0.3 | 03.71-05.26 | 5.00E-48 | 00.29 | 04.58 | 0.94 |
|  | E4 | 04.55±0.2 | 04.22-05.14 | 8.00E-12 | 00.42 | 05.16 | 0.78 |
|  | E5 | 04.57±0.2 | 04.17-05.23 | 3.00E-10 | 00.46 | 05.80 | 0.75 |
|  | E6 | 04.56±0.3 | 04.12-05.33 | 4.00E-38 | 00.30 | 04.61 | 0.92 |
| HI | E1 | 21.70±4.1 | 10.94-29.77 | 4.00E-20 | 04.81 | 11.63 | 0.87 |
|  | E2 | 21.37±4.3 | 10.12-29.92 | 1.00E-22 | 04.59 | 11.16 | 0.89 |
|  | E3 | 21.53±4.6 | 09.49-30.55 | 2.00E-52 | 02.99 | 09.82 | 0.95 |
|  | E4 | 21.12±3.9 | 12.49-29.38 | 3.00E-19 | 04.80 | 12.39 | 0.85 |
|  | E5 | 21.03±3.7 | 13.45-29.53 | 1.00E-14 | 05.45 | 14.52 | 0.81 |
|  | E6 | 21.08±4.3 | 11.92-29.62 | 4.00E-48 | 03.32 | 11.44 | 0.94 |

**Table S3: Five best performing genotypes under Ludhiana BLUP (L) and Gurdaspur BLUP (G) environments**

| S. No. | Env | DtF | DtM | PHM | NpP | IL | CpP | PpP | SpP | BYpP | YpP | HSW | HI |
| --- | --- | --- | --- | --- | --- | --- | --- | --- | --- | --- | --- | --- | --- |
| 1 | L | T9 | PU15-38 | IC328783 | PDU1 | IC519918 | MASH218 | IC370938 | KUG489 | NDU2-13-1 | KUG586 | IC557431 | KUG489 |
| 2 | L | KARS-88 | PU15-30 | IC530625 | IC331226 | IC281997 | IC328783 | MASH218 | PATHANKOT | KUG662 | MASH218 | PDU1 | KPU405 |
| 3 | L | KUG673 | IC530476 | IC316310 | IC393553 | IC471986 | KUG744 | IC519836 | KUG503 | IC530625 | IC471986 | PU6 | IC330901 |
| 4 | L | PU15-38 | KUG678 | MASH218 | IC330912 | PU11-25 | IC140817 | IC261171 | OBG35 | KUG586 | IC370938 | IPU2-33 | IC370938 |
| 5 | L | IC296661 | PU11-25 | NDU2-13-1 | LBG788 | IC519836 | MASH479 | IC530476 | IC427980 | IC471986 | MASH479 | IC370938 | MASH218 |
| 1 | G | KUG586 | KUG586 | IC530625 | IC328783 | PU15-44 | OBG35 | IC557431 | IC257654 | IC274597 | MASH1008 | IC557433 | IC396752 |
| 2 | G | OBG35 | IC569464 | IC328783 | IC530481 | PU11-14 | IC274597 | OBG35 | IC396752 | IC427980 | IC396752 | KARS-88 | IC398148 |
| 3 | G | IPU94-2 | KUG673 | IC330880 | IC530625 | PU15-28 | IC279538 | IC274597 | IC274597 | IC140817 | IC530607 | MASH479 | KUG673 |
| 4 | G | KUG673 | IC557431 | IC296661 | PU11-14 | KUG744 | TV-99-2 | IC321654 | V.sylvestris | MASH1008 | IC398148 | IC316310 | VERBEN7 |
| 5 | G | LBG787 | KUG678 | IC316310 | IC530607 | PU11-25 | IC530630 | NDU2-13-1 | IC279538 | OBG35 | IC557431 | KPU405 | IC472000 |

**Table S4: SNP distribution and coverage per individual chromosome of 100 of *V. mungo* germplasm lines against *Vigna radiata* genome**

| **Chr.** | **Size (Mb)** | **Raw Data** | | **After Filtering** | |
| --- | --- | --- | --- | --- | --- |
|  |  | Total SNPs | SNPs/Mb | Total SNPs | SNPs/Mb |
| 1 | 36.50 | 287906 | 7887.84 | 628 | 17.21 |
| 2 | 25.36 | 195053 | 7691.36 | 320 | 12.62 |
| 3 | 12.95 | 99572 | 7688.96 | 63 | 04.86 |
| 4 | 20.81 | 184038 | 8843.73 | 387 | 18.60 |
| 5 | 37.18 | 283594 | 7627.60 | 398 | 10.70 |
| 6 | 37.44 | 289471 | 7731.60 | 635 | 16.96 |
| 7 | 55.60 | 445155 | 8006.38 | 666 | 11.98 |
| 8 | 45.73 | 356526 | 7796.33 | 687 | 15.02 |
| 9 | 21.01 | 177687 | 8457.26 | 352 | 16.75 |
| 10 | 21.00 | 165077 | 7860.81 | 249 | 11.86 |
| 11 | 19.73 | 155385 | 7875.57 | 238 | 12.06 |
| Contigs | 68.63 | 910484 | 13266.56 | 2344 | 34.15 |
| Total | 401.94 | 3549948 | 8832.03 | 4623 | 11.50 |
| Average Chr. | 33.50 | 295829 | 8394.50 | 580.58 | 15.23 |

# Chromosome (Chr.), Million base pairs (Mb)

**Table S5: Allele frequency divergence (net nucleotide diversity) computed using estimates of P between subgroups and germplasm diversity**

| **Sub-pop.** | **1** | **2** | **3** | **4** | **O.M.P.** | **H_0_** | **Mean F_ST_** |
| --- | --- | --- | --- | --- | --- | --- | --- |
| **1** | - |  |  |  | 0.252 | 0.3780 | 0.0203 |
| **2** | 0.0742 | - |  |  | 0.069 | 0.2719 | 0.4537 |
| **3** | 0.0784 | 0.1237 | - |  | 0.253 | 0.0551 | 0.8040 |
| **4** | 0.0813 | 0.1245 | 0.0141 | - | 0.426 | 0.0250 | 0.9098 |

# Sub-population (Sub-pop.), Overall Membership Proportion (O.M.P), Expected heterozygosity (H_0_), (Mean F_ST_)

**Table S6: MTAs found in Genome wide association study for 100 blackgram germplasm lines across Env (environments) -** Ludhiana 2019 (E1); Ludhiana 2020 (E2); Ludhiana BLUP (E3); Gurdaspur 2019 (E4); Gurdaspur 2020 (E5) and Gurdaspur BLUP (E6) using methods FarmCPU and MLM along with positions in the respective chromosomes.

| **QTL** | **Trait** | **Env** | **Method** | **SNP** | **Chr** | **Position** | **PosMb** | **logP** | **maf** | **effect** | **PVE(%)** |
| --- | --- | --- | --- | --- | --- | --- | --- | --- | --- | --- | --- |
| *Q.DtF.10* | *DtF* | E1 | FarmCPU | S10.1.9527186 | 10 | 9527186 | 9.5272 | 5.1118 | 0.07 | 1.2261 | 20.038 |
|  |  | E2 | FarmCPU | S10.1.9527186 | 10 | 9527186 | 9.5272 | 3.9996 | 0.07 | 1.2122 | 14.022 |
|  |  | E3 | FarmCPU | S10.1.9527186 | 10 | 9527186 | 9.5272 | 4.9208 | 0.07 | 1.68 | 17.947 |
|  |  | E1 | MLM | S10.1.9527186 | 10 | 9527186 | 9.5272 | 4.4597 | 0.07 | 1.117 | 16.896 |
|  |  | E2 | MLM | S10.1.9527186 | 10 | 9527186 | 9.5272 | 3.4509 | 0.07 | 1.1419 | 13.689 |
|  |  | E3 | MLM | S10.1.9527186 | 10 | 9527186 | 9.5272 | 4.1457 | 0.07 | 1.5481 | 16.803 |
| *Q.PHM.3.1* | *PHM* | E4 | FarmCPU | S3.1.7993147 | 3 | 7993147 | 7.9931 | 3.5163 | 0.115 | -5.6686 | 16.262 |
|  |  | E5 | FarmCPU | S3.1.7993147 | 3 | 7993147 | 7.9931 | 3.6971 | 0.115 | -6.0609 | 16.983 |
|  |  | E6 | FarmCPU | S3.1.7993147 | 3 | 7993147 | 7.9931 | 3.6566 | 0.115 | -6.3321 | 16.506 |
| *Q.PHM.3.2* | *PHM* | E1 | FarmCPU | S3.1.8219594 | 3 | 8219594 | 8.2196 | 3.1879 | 0.15 | -3.0835 | 19.002 |
|  |  | E2 | FarmCPU | S3.1.8219594 | 3 | 8219594 | 8.2196 | 3.1888 | 0.15 | -2.9018 | 20.05 |
|  |  | E3 | FarmCPU | S3.1.8219594 | 3 | 8219594 | 8.2196 | 3.2337 | 0.15 | -3.0667 | 19.919 |
| *Q.PHM.4* | *PHM* | E1 | FarmCPU | S4.1.1259800 | 4 | 1259800 | 1.2598 | 3.9881 | 0.065 | 7.1935 | 18.209 |
|  |  | E2 | FarmCPU | S4.1.1259800 | 4 | 1259800 | 1.2598 | 3.7982 | 0.065 | 6.6011 | 16.242 |
|  |  | E3 | FarmCPU | S4.1.1259800 | 4 | 1259800 | 1.2598 | 3.8912 | 0.065 | 7.009 | 17.084 |
|  |  | E4 | FarmCPU | S4.1.1259800 | 4 | 1259800 | 1.2598 | 3.1061 | 0.065 | 9.9509 | 16.692 |
|  |  | E5 | FarmCPU | S4.1.1259800 | 4 | 1259800 | 1.2598 | 3.0509 | 0.065 | 10.261 | 16.717 |
|  |  | E6 | FarmCPU | S4.1.1259800 | 4 | 1259800 | 1.2598 | 3.0761 | 0.065 | 10.8299 | 16.46 |
|  |  | E4 | MLM | S4.1.1259800 | 4 | 1259800 | 1.2598 | 3.0978 | 0.065 | 10.6217 | 10.105 |
|  |  | E5 | MLM | S4.1.1259800 | 4 | 1259800 | 1.2598 | 3.0957 | 0.065 | 10.9508 | 10.059 |
|  |  | E6 | MLM | S4.1.1259800 | 4 | 1259800 | 1.2598 | 3.1226 | 0.065 | 11.6496 | 10.076 |
| *Q.PHM.6.1* | *PHM* | E4 | FarmCPU | S6.1.23358875 | 6 | 23358875 | 23.3589 | 3.6065 | 0.105 | 7.9794 | 10.833 |
|  |  | E5 | FarmCPU | S6.1.23358875 | 6 | 23358875 | 23.3589 | 3.4814 | 0.105 | 8.1524 | 10.539 |
|  |  | E6 | FarmCPU | S6.1.23358875 | 6 | 23358875 | 23.3589 | 3.4745 | 0.105 | 8.5575 | 10.469 |
| *Q.PHM.6.2* | *PHM* | E1 | FarmCPU | S6.1.25029431 | 6 | 25029431 | 25.0294 | 3.5422 | 0.205 | -3.4219 | 14.643 |
|  |  | E2 | FarmCPU | S6.1.25029431 | 6 | 25029431 | 25.0294 | 3.3731 | 0.205 | -3.1368 | 14.271 |
|  |  | E3 | FarmCPU | S6.1.25029431 | 6 | 25029431 | 25.0294 | 3.4664 | 0.205 | -3.3382 | 14.698 |
| *Q.PHM.8* | *PHM* | E1 | FarmCPU | S8.1.13991269 | 8 | 13991269 | 13.9913 | 4.1057 | 0.055 | 7.0437 | 17.489 |
|  |  | E2 | FarmCPU | S8.1.13991269 | 8 | 13991269 | 13.9913 | 4.2132 | 0.055 | 6.7236 | 17.267 |
|  |  | E3 | FarmCPU | S8.1.13991269 | 8 | 13991269 | 13.9913 | 4.2062 | 0.055 | 7.0402 | 17.697 |
|  |  | E4 | FarmCPU | S8.1.13991269 | 8 | 13991269 | 13.9913 | 4.064 | 0.055 | 11.0777 | 10.121 |
|  |  | E5 | FarmCPU | S8.1.13991269 | 8 | 13991269 | 13.9913 | 4.1232 | 0.055 | 11.617 | 11.653 |
|  |  | E6 | FarmCPU | S8.1.13991269 | 8 | 13991269 | 13.9913 | 4.1599 | 0.055 | 12.2652 | 11.234 |
|  |  | E1 | MLM | S8.1.13991269 | 8 | 13991269 | 13.9913 | 3.3191 | 0.055 | 6.1684 | 10.416 |
|  |  | E2 | MLM | S8.1.13991269 | 8 | 13991269 | 13.9913 | 3.3526 | 0.055 | 5.9991 | 10.613 |
|  |  | E3 | MLM | S8.1.13991269 | 8 | 13991269 | 13.9913 | 3.3945 | 0.055 | 6.216 | 10.728 |
|  |  | E4 | MLM | S8.1.13991269 | 8 | 13991269 | 13.9913 | 3.7085 | 0.055 | 10.756 | 12.646 |
|  |  | E5 | MLM | S8.1.13991269 | 8 | 13991269 | 13.9913 | 3.8079 | 0.055 | 11.2984 | 13.021 |
|  |  | E6 | MLM | S8.1.13991269 | 8 | 13991269 | 13.9913 | 3.8356 | 0.055 | 11.9305 | 13.02 |
| *Q.PHM.11.1* | *PHM* | E4 | FarmCPU | S11.1.16313748 | 11 | 16313748 | 16.3137 | 3.0742 | 0.19 | 4.6239 | 14.951 |
|  |  | E5 | FarmCPU | S11.1.16313748 | 11 | 16313748 | 16.3137 | 3.0774 | 0.19 | 4.8178 | 15.341 |
|  |  | E6 | FarmCPU | S11.1.16313748 | 11 | 16313748 | 16.3137 | 3.1353 | 0.19 | 5.1138 | 15.263 |
| *Q.PHM.11.2* | *PHM* | E1 | FarmCPU | S11.1.16898133 | 11 | 16898133 | 16.8981 | 3.0583 | 0.17 | 4.5379 | 16.279 |
|  |  | E2 | FarmCPU | S11.1.16898133 | 11 | 16898133 | 16.8981 | 3.0974 | 0.17 | 4.2992 | 15.998 |
|  |  | E3 | FarmCPU | S11.1.16898133 | 11 | 16898133 | 16.8981 | 3.055 | 0.17 | 4.4754 | 15.99 |
|  |  | E1 | FarmCPU | S11.1.16898169 | 11 | 16898169 | 16.8982 | 3.066 | 0.165 | 4.53 | 16.439 |
|  |  | E2 | FarmCPU | S11.1.16898169 | 11 | 16898169 | 16.8982 | 3.0114 | 0.165 | 4.2197 | 15.792 |
|  |  | E3 | FarmCPU | S11.1.16898169 | 11 | 16898169 | 16.8982 | 3.0204 | 0.165 | 4.4335 | 16.003 |
|  |  | E1 | FarmCPU | S11.1.16898170 | 11 | 16898170 | 16.8982 | 3.066 | 0.165 | 4.53 | 16.439 |
|  |  | E2 | FarmCPU | S11.1.16898170 | 11 | 16898170 | 16.8982 | 3.0114 | 0.165 | 4.2197 | 15.792 |
|  |  | E3 | FarmCPU | S11.1.16898170 | 11 | 16898170 | 16.8982 | 3.0204 | 0.165 | 4.4335 | 16.003 |
|  |  | E1 | FarmCPU | S11.1.16898225 | 11 | 16898225 | 16.8982 | 3.2831 | 0.155 | 4.614 | 15.193 |
|  |  | E2 | FarmCPU | S11.1.16898225 | 11 | 16898225 | 16.8982 | 3.5826 | 0.155 | 4.5519 | 15.776 |
|  |  | E3 | FarmCPU | S11.1.16898225 | 11 | 16898225 | 16.8982 | 3.4443 | 0.155 | 4.6729 | 15.565 |
| *Q.BpP.6* | *BpP* | E4 | MLM | S6.1.22219189 | 6 | 22219189 | 22.2192 | 3.6163 | 0.19 | -0.4196 | 13.644 |
|  |  | E5 | MLM | S6.1.22219189 | 6 | 22219189 | 22.2192 | 3.9567 | 0.19 | -0.4402 | 15.211 |
|  |  | E6 | MLM | S6.1.22219189 | 6 | 22219189 | 22.2192 | 3.7615 | 0.19 | -0.6003 | 14.341 |
| *Q.NpP.4* | *NpP* | E4 | FarmCPU | S4.1.1343019 | 4 | 1343019 | 1.343 | 3.5494 | 0.065 | 1.8453 | 14.355 |
|  |  | E5 | FarmCPU | S4.1.1343019 | 4 | 1343019 | 1.343 | 3.4553 | 0.065 | 1.7188 | 14.639 |
|  |  | E6 | FarmCPU | S4.1.1343019 | 4 | 1343019 | 1.343 | 3.6303 | 0.065 | 2.2705 | 15.003 |
|  |  | E4 | MLM | S4.1.1343019 | 4 | 1343019 | 1.343 | 3.3071 | 0.065 | 1.8335 | 10.598 |
|  |  | E5 | MLM | S4.1.1343019 | 4 | 1343019 | 1.343 | 3.3408 | 0.065 | 1.7415 | 10.275 |
|  |  | E6 | MLM | S4.1.1343019 | 4 | 1343019 | 1.343 | 3.4822 | 0.065 | 2.2851 | 10.85 |
| *Q.NpP.6* | *NpP* | E1 | FarmCPU | S6.1.32970231 | 6 | 32970231 | 32.9702 | 3.7621 | 0.095 | 1.312 | 19.693 |
|  |  | E2 | FarmCPU | S6.1.32970231 | 6 | 32970231 | 32.9702 | 3.4372 | 0.095 | 1.6186 | 17.534 |
|  |  | E3 | FarmCPU | S6.1.32970231 | 6 | 32970231 | 32.9702 | 3.5832 | 0.095 | 1.7559 | 18.857 |
|  |  | E1 | FarmCPU | S6.1.32970252 | 6 | 32970252 | 32.9703 | 4.3686 | 0.055 | 1.7678 | 20.99 |
|  |  | E2 | FarmCPU | S6.1.32970252 | 6 | 32970252 | 32.9703 | 3.1546 | 0.055 | 1.9256 | 15.691 |
|  |  | E3 | FarmCPU | S6.1.32970252 | 6 | 32970252 | 32.9703 | 3.8288 | 0.055 | 2.267 | 19.048 |
|  |  | E1 | FarmCPU | S6.1.32970258 | 6 | 32970258 | 32.9703 | 4.7773 | 0.085 | 1.5432 | 23.887 |
|  |  | E2 | FarmCPU | S6.1.32970258 | 6 | 32970258 | 32.9703 | 4.1169 | 0.085 | 1.8524 | 20.326 |
|  |  | E3 | FarmCPU | S6.1.32970258 | 6 | 32970258 | 32.9703 | 4.4295 | 0.085 | 2.0396 | 22.292 |
|  |  | E1 | MLM | S6.1.32970258 | 6 | 32970258 | 32.9703 | 3.5374 | 0.085 | 1.3629 | 11.733 |
|  |  | E2 | MLM | S6.1.32970258 | 6 | 32970258 | 32.9703 | 3.4138 | 0.085 | 1.7846 | 12.836 |
|  |  | E3 | MLM | S6.1.32970258 | 6 | 32970258 | 32.9703 | 3.4366 | 0.085 | 1.8555 | 12.377 |
| *Q.IL.1.1* | *IL* | E4 | FarmCPU | S1.1.2636313 | 1 | 2636313 | 2.6363 | 4.2055 | 0.115 | 0.4127 | 14.149 |
|  |  | E5 | FarmCPU | S1.1.2636313 | 1 | 2636313 | 2.6363 | 4.433 | 0.115 | 0.4243 | 15.205 |
|  |  | E6 | FarmCPU | S1.1.2636313 | 1 | 2636313 | 2.6363 | 4.4078 | 0.115 | 0.4871 | 14.843 |
|  |  | E4 | MLM | S1.1.2636313 | 1 | 2636313 | 2.6363 | 3.6947 | 0.115 | 0.4127 | 14.129 |
|  |  | E5 | MLM | S1.1.2636313 | 1 | 2636313 | 2.6363 | 3.849 | 0.115 | 0.4243 | 15.201 |
|  |  | E6 | MLM | S1.1.2636313 | 1 | 2636313 | 2.6363 | 3.7944 | 0.115 | 0.4871 | 14.693 |
| *Q.IL.1.2* | *IL* | E1 | FarmCPU | S1.1.35117546 | 1 | 35117546 | 35.1175 | 3.4155 | 0.06 | 0.6732 | 14.538 |
|  |  | E2 | FarmCPU | S1.1.35117546 | 1 | 35117546 | 35.1175 | 3.2778 | 0.06 | 0.6711 | 13.52 |
|  |  | E3 | FarmCPU | S1.1.35117546 | 1 | 35117546 | 35.1175 | 3.356 | 0.06 | 0.7035 | 14.089 |
| *Q.IL.5* | *IL* | E1 | MLM | S5.1.15033908 | 5 | 15033908 | 15.0339 | 3.4257 | 0.08 | 0.7585 | 13.275 |
|  |  | E2 | MLM | S5.1.15033908 | 5 | 15033908 | 15.0339 | 3.5087 | 0.08 | 0.7993 | 13.796 |
|  |  | E3 | MLM | S5.1.15033908 | 5 | 15033908 | 15.0339 | 3.4791 | 0.08 | 0.8146 | 13.589 |
| *Q.IL.8* | *IL* | E1 | FarmCPU | S8.1.13991269 | 8 | 13991269 | 13.9913 | 4.2343 | 0.055 | 0.7812 | 14.46 |
|  |  | E2 | FarmCPU | S8.1.13991269 | 8 | 13991269 | 13.9913 | 4.4634 | 0.055 | 0.8191 | 15.059 |
|  |  | E3 | FarmCPU | S8.1.13991269 | 8 | 13991269 | 13.9913 | 4.3615 | 0.055 | 0.8372 | 14.825 |
|  |  | E1 | MLM | S8.1.13991269 | 8 | 13991269 | 13.9913 | 3.6056 | 0.055 | 0.7727 | 14.149 |
|  |  | E2 | MLM | S8.1.13991269 | 8 | 13991269 | 13.9913 | 3.685 | 0.055 | 0.8047 | 14.665 |
|  |  | E3 | MLM | S8.1.13991269 | 8 | 13991269 | 13.9913 | 3.6602 | 0.055 | 0.8254 | 14.476 |
| *Q.CpP.1* | *CpP* | E4 | FarmCPU | S1.1.25545554 | 1 | 25545554 | 25.5456 | 4.1319 | 0.1 | -1.8845 | 23.8 |
|  |  | E5 | FarmCPU | S1.1.25545554 | 1 | 25545554 | 25.5456 | 4.2255 | 0.1 | -1.8188 | 24.122 |
|  |  | E6 | FarmCPU | S1.1.25545554 | 1 | 25545554 | 25.5456 | 4.1785 | 0.1 | -2.1507 | 24.26 |
| *Q.CpP.4* | *CpP* | E4 | MLM | S4.1.774872 | 4 | 774872 | 0.7749 | 3.4744 | 0.07 | -2.7122 | 10.358 |
|  |  | E5 | MLM | S4.1.774872 | 4 | 774872 | 0.7749 | 3.5784 | 0.07 | -2.6482 | 10.32 |
|  |  | E6 | MLM | S4.1.774872 | 4 | 774872 | 0.7749 | 3.5768 | 0.07 | -3.1131 | 10.448 |
| *Q.CpP.7* | *CpP* | E1 | FarmCPU | S7.1.36781633 | 7 | 36781633 | 36.7816 | 4.1238 | 0.06 | 1.5314 | 14.46 |
|  |  | E2 | FarmCPU | S7.1.36781633 | 7 | 36781633 | 36.7816 | 4.8327 | 0.06 | 1.9554 | 16.933 |
|  |  | E3 | FarmCPU | S7.1.36781633 | 7 | 36781633 | 36.7816 | 4.4572 | 0.06 | 1.9962 | 15.922 |
|  |  | E1 | MLM | S7.1.36781633 | 7 | 36781633 | 36.7816 | 3.571 | 0.06 | 1.5314 | 13.37 |
|  |  | E2 | MLM | S7.1.36781633 | 7 | 36781633 | 36.7816 | 4.0357 | 0.06 | 1.9554 | 15.637 |
|  |  | E3 | MLM | S7.1.36781633 | 7 | 36781633 | 36.7816 | 3.8541 | 0.06 | 1.9962 | 14.712 |
| *Q.CpP.9* | *CpP* | E1 | FarmCPU | S9.1.9823250 | 9 | 9823250 | 9.8233 | 3.3808 | 0.065 | 1.3476 | 14.557 |
|  |  | E2 | FarmCPU | S9.1.9823250 | 9 | 9823250 | 9.8233 | 3.1992 | 0.065 | 1.5363 | 13.505 |
|  |  | E3 | FarmCPU | S9.1.9823250 | 9 | 9823250 | 9.8233 | 3.2717 | 0.065 | 1.6571 | 14.218 |
| *Q.CpP.10* | *CpP* | E1 | FarmCPU | S10.1.1346198 | 10 | 1346198 | 1.3462 | 3.5238 | 0.185 | 0.5922 | 12.217 |
|  |  | E2 | FarmCPU | S10.1.1346198 | 10 | 1346198 | 1.3462 | 3.6663 | 0.185 | 0.7112 | 12.399 |
|  |  | E3 | FarmCPU | S10.1.1346198 | 10 | 1346198 | 1.3462 | 3.5792 | 0.185 | 0.7477 | 12.487 |
|  |  | E1 | MLM | S10.1.1346198 | 10 | 1346198 | 1.3462 | 3.1198 | 0.185 | 0.5922 | 11.297 |
|  |  | E2 | MLM | S10.1.1346198 | 10 | 1346198 | 1.3462 | 3.1943 | 0.185 | 0.7112 | 11.686 |
|  |  | E3 | MLM | S10.1.1346198 | 10 | 1346198 | 1.3462 | 3.1982 | 0.185 | 0.7477 | 11.66 |
|  |  | E1 | FarmCPU | S10.1.1346205 | 10 | 1346205 | 1.3462 | 3.8551 | 0.06 | 1.4676 | 15.149 |
|  |  | E2 | FarmCPU | S10.1.1346205 | 10 | 1346205 | 1.3462 | 4.6676 | 0.06 | 1.9078 | 18.014 |
|  |  | E3 | FarmCPU | S10.1.1346205 | 10 | 1346205 | 1.3462 | 4.2381 | 0.06 | 1.9314 | 16.817 |
|  |  | E1 | MLM | S10.1.1346205 | 10 | 1346205 | 1.3462 | 3.3717 | 0.06 | 1.4676 | 12.447 |
|  |  | E2 | MLM | S10.1.1346205 | 10 | 1346205 | 1.3462 | 3.9213 | 0.06 | 1.9078 | 15.087 |
|  |  | E3 | MLM | S10.1.1346205 | 10 | 1346205 | 1.3462 | 3.6948 | 0.06 | 1.9314 | 13.959 |
|  |  | E1 | FarmCPU | S10.1.1346249 | 10 | 1346249 | 1.3462 | 3.1734 | 0.095 | 1.0986 | 11.399 |
|  |  | E2 | FarmCPU | S10.1.1346249 | 10 | 1346249 | 1.3462 | 3.4583 | 0.095 | 1.3536 | 12.288 |
|  |  | E3 | FarmCPU | S10.1.1346249 | 10 | 1346249 | 1.3462 | 3.3004 | 0.095 | 1.4055 | 12.019 |
| *Q.PL.1* | *PL* | E1 | MLM | S1.1.30381382 | 1 | 30381382 | 30.3814 | 3.1612 | 0.075 | -0.1592 | 11.357 |
|  |  | E2 | MLM | S1.1.30381382 | 1 | 30381382 | 30.3814 | 3.0414 | 0.075 | -0.1604 | 10.934 |
|  |  | E3 | MLM | S1.1.30381382 | 1 | 30381382 | 30.3814 | 3.2522 | 0.075 | -0.1458 | 11.747 |
| *Q.SpP.3* | *SpP* | E1 | FarmCPU | S3.1.8767931 | 3 | 8767931 | 8.7679 | 3.6644 | 0.09 | 0.3757 | 14.329 |
|  |  | E2 | FarmCPU | S3.1.8767931 | 3 | 8767931 | 8.7679 | 3.5543 | 0.09 | 0.4074 | 14.291 |
|  |  | E3 | FarmCPU | S3.1.8767931 | 3 | 8767931 | 8.7679 | 4.1296 | 0.09 | 0.4397 | 15.931 |
|  |  | E1 | MLM | S3.1.8767931 | 3 | 8767931 | 8.7679 | 3.4451 | 0.09 | 0.3897 | 14.059 |
|  |  | E2 | MLM | S3.1.8767931 | 3 | 8767931 | 8.7679 | 3.3556 | 0.09 | 0.4268 | 13.574 |
|  |  | E3 | MLM | S3.1.8767931 | 3 | 8767931 | 8.7679 | 3.7625 | 0.09 | 0.4589 | 15.654 |
| *Q.SpP.6* | *SpP* | E4 | MLM | S6.1.29235889 | 6 | 29235889 | 29.2359 | 3.3721 | 0.13 | -0.3538 | 13.757 |
|  |  | E5 | MLM | S6.1.29235889 | 6 | 29235889 | 29.2359 | 3.5631 | 0.13 | -0.3419 | 14.473 |
|  |  | E6 | MLM | S6.1.29235889 | 6 | 29235889 | 29.2359 | 3.501 | 0.13 | -0.4234 | 14.263 |
| *Q.SpP.8.1* | *SpP* | E1 | MLM | S8.1.13990816 | 8 | 13990816 | 13.9908 | 3.2691 | 0.165 | -0.3222 | 13.17 |
|  |  | E2 | MLM | S8.1.13990816 | 8 | 13990816 | 13.9908 | 3.2343 | 0.165 | -0.3547 | 12.965 |
|  |  | E3 | MLM | S8.1.13990816 | 8 | 13990816 | 13.9908 | 3.0808 | 0.165 | -0.3413 | 12.201 |
| *Q.SpP.8.2* | *SpP* | E4 | MLM | S8.1.16767849 | 8 | 16767849 | 16.7678 | 3.0986 | 0.145 | 0.3228 | 12.379 |
|  |  | E5 | MLM | S8.1.16767849 | 8 | 16767849 | 16.7678 | 3.0926 | 0.145 | 0.2999 | 12.13 |
|  |  | E6 | MLM | S8.1.16767849 | 8 | 16767849 | 16.7678 | 3.1283 | 0.145 | 0.3782 | 12.395 |
| *Q.BYpP.8* | *BYpP* | E4 | FarmCPU | S8.1.33339056 | 8 | 33339056 | 33.3391 | 3.2978 | 0.15 | 3.4286 | 13.089 |
|  |  | E5 | FarmCPU | S8.1.33339056 | 8 | 33339056 | 33.3391 | 3.5682 | 0.15 | 3.5887 | 13.864 |
|  |  | E6 | FarmCPU | S8.1.33339056 | 8 | 33339056 | 33.3391 | 3.4848 | 0.15 | 3.7945 | 13.775 |
| *Q.BYpP.9* | *BYpP* | E1 | FarmCPU | S9.1.5335251 | 9 | 5335251 | 5.3353 | 3.2126 | 0.175 | -1.7959 | 11.465 |
|  |  | E2 | FarmCPU | S9.1.5335251 | 9 | 5335251 | 5.3353 | 3.3168 | 0.175 | -1.9943 | 13.806 |
|  |  | E3 | FarmCPU | S9.1.5335251 | 9 | 5335251 | 5.3353 | 3.3148 | 0.175 | -2.0795 | 13.007 |
| *Q.YpP.4.1* | *YpP* | E1 | MLM | S4.1.9615705 | 4 | 9615705 | 9.6157 | 3.2879 | 0.07 | 1.3004 | 12.919 |
|  |  | E2 | MLM | S4.1.9615705 | 4 | 9615705 | 9.6157 | 3.0929 | 0.07 | 1.254 | 12.017 |
|  |  | E3 | MLM | S4.1.9615705 | 4 | 9615705 | 9.6157 | 3.2364 | 0.07 | 1.3938 | 12.669 |
| *Q.YpP.4.2* | *YpP* | E4 | FarmCPU | S4.1.11912711 | 4 | 11912711 | 11.9127 | 3.8732 | 0.095 | 1.1828 | 13.391 |
|  |  | E5 | FarmCPU | S4.1.11912711 | 4 | 11912711 | 11.9127 | 3.2767 | 0.095 | 1.0099 | 11.564 |
|  |  | E6 | FarmCPU | S4.1.11912711 | 4 | 11912711 | 11.9127 | 3.6025 | 0.095 | 1.1945 | 12.541 |
|  |  | E4 | MLM | S4.1.11912711 | 4 | 11912711 | 11.9127 | 3.6643 | 0.095 | 1.1979 | 12.793 |
|  |  | E5 | MLM | S4.1.11912711 | 4 | 11912711 | 11.9127 | 3.1986 | 0.095 | 1.0303 | 10.436 |
|  |  | E6 | MLM | S4.1.11912711 | 4 | 11912711 | 11.9127 | 3.4516 | 0.095 | 1.214 | 11.667 |
| *Q.YpP.5* | *YpP* | E4 | FarmCPU | S5.1.35989113 | 5 | 35989113 | 35.9891 | 3.5121 | 0.08 | -1.1679 | 10.536 |
|  |  | E5 | FarmCPU | S5.1.35989113 | 5 | 35989113 | 35.9891 | 3.8343 | 0.08 | -1.1431 | 11.382 |
|  |  | E6 | FarmCPU | S5.1.35989113 | 5 | 35989113 | 35.9891 | 3.7002 | 0.08 | -1.2605 | 11.018 |
|  |  | E4 | MLM | S5.1.35989113 | 5 | 35989113 | 35.9891 | 3.1452 | 0.08 | -1.1405 | 10.575 |
|  |  | E5 | MLM | S5.1.35989113 | 5 | 35989113 | 35.9891 | 3.4291 | 0.08 | -1.127 | 11.382 |
|  |  | E6 | MLM | S5.1.35989113 | 5 | 35989113 | 35.9891 | 3.3001 | 0.08 | -1.2365 | 11.032 |
| *Q.YpP.8* | *YpP* | E1 | FarmCPU | S8.1.19533014 | 8 | 19533014 | 19.533 | 3.8014 | 0.17 | -0.9569 | 10.918 |
|  |  | E2 | FarmCPU | S8.1.19533014 | 8 | 19533014 | 19.533 | 3.7945 | 0.17 | -0.9558 | 10.914 |
|  |  | E3 | FarmCPU | S8.1.19533014 | 8 | 19533014 | 19.533 | 3.7596 | 0.17 | -1.0298 | 10.751 |
|  |  | E1 | MLM | S8.1.19533014 | 8 | 19533014 | 19.533 | 3.3709 | 0.17 | -0.9529 | 13.327 |
|  |  | E2 | MLM | S8.1.19533014 | 8 | 19533014 | 19.533 | 3.3549 | 0.17 | -0.9515 | 13.3 |
|  |  | E3 | MLM | S8.1.19533014 | 8 | 19533014 | 19.533 | 3.3417 | 0.17 | -1.0257 | 13.184 |
| *Q.HI.1* | *HI* | E1 | FarmCPU | S1.1.8549788 | 1 | 8549788 | 8.5498 | 3.8719 | 0.095 | -4.1399 | 10.132 |
|  |  | E2 | FarmCPU | S1.1.8549788 | 1 | 8549788 | 8.5498 | 4.295 | 0.095 | -4.5706 | 12.345 |
|  |  | E3 | FarmCPU | S1.1.8549788 | 1 | 8549788 | 8.5498 | 4.2388 | 0.095 | -4.7838 | 11.425 |
|  |  | E1 | MLM | S1.1.8549788 | 1 | 8549788 | 8.5498 | 3.5609 | 0.095 | -4.1626 | 14.526 |
|  |  | E2 | MLM | S1.1.8549788 | 1 | 8549788 | 8.5498 | 3.9142 | 0.095 | -4.5803 | 16.607 |
|  |  | E3 | MLM | S1.1.8549788 | 1 | 8549788 | 8.5498 | 3.8081 | 0.095 | -4.8079 | 15.933 |
| *Q.HI.5* | *HI* | E4 | FarmCPU | S5.1.29623709 | 5 | 29623709 | 29.6237 | 3.3325 | 0.145 | -3.0737 | 10.273 |
|  |  | E5 | FarmCPU | S5.1.29623709 | 5 | 29623709 | 29.6237 | 3.6104 | 0.145 | -3.0562 | 11.887 |
|  |  | E6 | FarmCPU | S5.1.29623709 | 5 | 29623709 | 29.6237 | 3.5094 | 0.145 | -3.4666 | 11.234 |
| *Q.HI.6* | *HI* | E4 | MLM | S6.1.16902154 | 6 | 16902154 | 16.9022 | 3.2179 | 0.08 | 3.9188 | 12.842 |
|  |  | E5 | MLM | S6.1.16902154 | 6 | 16902154 | 16.9022 | 3.8436 | 0.08 | 4.1457 | 15.917 |
|  |  | E6 | MLM | S6.1.16902154 | 6 | 16902154 | 16.9022 | 3.5694 | 0.08 | 4.5656 | 14.564 |
| *Q.HI.7* | *HI* | E4 | FarmCPU | S7.1.49416375 | 7 | 49416375 | 49.4164 | 3.5877 | 0.16 | -3.1184 | 16.978 |
|  |  | E5 | FarmCPU | S7.1.49416375 | 7 | 49416375 | 49.4164 | 3.5857 | 0.16 | -2.9683 | 18.571 |
|  |  | E6 | FarmCPU | S7.1.49416375 | 7 | 49416375 | 49.4164 | 3.6277 | 0.16 | -3.4401 | 18.045 |
| *Q.HI.8.1* | *HI* | E4 | MLM | S8.1.4181215 | 8 | 4181215 | 4.1812 | 3.6928 | 0.095 | 4.374 | 15.245 |
|  |  | E5 | MLM | S8.1.4181215 | 8 | 4181215 | 4.1812 | 3.0087 | 0.095 | 3.6445 | 11.729 |
|  |  | E6 | MLM | S8.1.4181215 | 8 | 4181215 | 4.1812 | 3.3758 | 0.095 | 4.5191 | 13.586 |
| *Q.HI.8.2* | *HI* | E1 | FarmCPU | S8.1.19533014 | 8 | 19533014 | 19.533 | 3.6593 | 0.17 | -3.234 | 11.263 |
|  |  | E2 | FarmCPU | S8.1.19533014 | 8 | 19533014 | 19.533 | 3.5391 | 0.17 | -3.322 | 12.449 |
|  |  | E3 | FarmCPU | S8.1.19533014 | 8 | 19533014 | 19.533 | 3.7094 | 0.17 | -3.5888 | 12.072 |
|  |  | E1 | MLM | S8.1.19533014 | 8 | 19533014 | 19.533 | 3.3159 | 0.17 | -3.2202 | 13.291 |
|  |  | E2 | MLM | S8.1.19533014 | 8 | 19533014 | 19.533 | 3.1396 | 0.17 | -3.2524 | 12.617 |
|  |  | E3 | MLM | S8.1.19533014 | 8 | 19533014 | 19.533 | 3.2618 | 0.17 | -3.5455 | 13.138 |
| *Q.HI.10* | *HI* | E4 | MLM | S10.1.2365261 | 10 | 2365261 | 2.3653 | 3.1451 | 0.085 | -3.8991 | 12.48 |
|  |  | E5 | MLM | S10.1.2365261 | 10 | 2365261 | 2.3653 | 3.0781 | 0.085 | -3.6426 | 12.068 |
|  |  | E6 | MLM | S10.1.2365261 | 10 | 2365261 | 2.3653 | 3.1461 | 0.085 | -4.2602 | 12.443 |
| *Q.HSW.6* | *HSW* | E1 | FarmCPU | S6.1.8707985 | 6 | 8707985 | 8.708 | 3.4907 | 0.065 | -0.3237 | 10.817 |
|  |  | E2 | FarmCPU | S6.1.8707985 | 6 | 8707985 | 8.708 | 3.2914 | 0.065 | -0.2849 | 10.51 |
|  |  | E3 | FarmCPU | S6.1.8707985 | 6 | 8707985 | 8.708 | 3.4374 | 0.065 | -0.3472 | 10.822 |
| *Q.HSW.7* | *HSW* | E4 | FarmCPU | S7.1.20599131 | 7 | 20599131 | 20.5991 | 4.1018 | 0.08 | 0.2092 | 11.374 |
|  |  | E5 | FarmCPU | S7.1.20599131 | 7 | 20599131 | 20.5991 | 3.9394 | 0.08 | 0.2139 | 10.457 |
|  |  | E6 | FarmCPU | S7.1.20599131 | 7 | 20599131 | 20.5991 | 4.3788 | 0.08 | 0.2654 | 12.201 |
|  |  | E4 | MLM | S7.1.20599131 | 7 | 20599131 | 20.5991 | 3.3634 | 0.08 | 0.1898 | 10.307 |
|  |  | E5 | MLM | S7.1.20599131 | 7 | 20599131 | 20.5991 | 3.3524 | 0.08 | 0.2074 | 11.893 |
|  |  | E6 | MLM | S7.1.20599131 | 7 | 20599131 | 20.5991 | 3.5899 | 0.08 | 0.2455 | 12.339 |
| *Q.HSW.10* | *HSW* | E1 | FarmCPU | S10.1.20952550 | 10 | 20952550 | 20.9526 | 3.1092 | 0.135 | 0.2339 | 11.996 |
|  |  | E2 | FarmCPU | S10.1.20952550 | 10 | 20952550 | 20.9526 | 3.0539 | 0.135 | 0.2104 | 11.129 |
|  |  | E3 | FarmCPU | S10.1.20952550 | 10 | 20952550 | 20.9526 | 3.1235 | 0.135 | 0.2536 | 11.731 |

**Table S7: Significant allelic effect determination using t-test significance for all MTAs with respect to different characters.**

| **MTA** | **SNP** | **Alleles** | **Character** | **E1** | **E2** | **E3** | **E4** | **E5** | **E6** |
| --- | --- | --- | --- | --- | --- | --- | --- | --- | --- |
| *Q.DtF.10* | S10.1.9527186 | TT/CT | DtF | 0.00E+00 | 1.20E-04 | 1.00E-05 | 4.80E-01 | 7.80E-01 | 7.81E-01 |
| *Q.PHM.3.1* | S3.1.7993147 | AA/GG | PHM | 1.20E-04 | 7.00E-05 | 8.00E-05 | 2.10E-04 | 1.40E-04 | 1.49E-04 |
| *Q.PHM.3.2* | S3.1.8219594 | AA/GG | PHM | 6.00E-05 | 3.00E-05 | 4.00E-05 | 6.00E-03 | 5.00E-03 | 5.44E-03 |
| *Q.PHM.4* | S4.1.1259800 | CC/CT | PHM | 1.00E-05 | 3.00E-05 | 2.00E-05 | 2.00E-05 | 2.00E-05 | 2.41E-05 |
| *Q.PHM.6.1* | S6.1.23358875 | CC/CT | PHM | 4.10E-02 | 2.50E-02 | 3.00E-02 | 8.30E-04 | 9.80E-04 | 9.83E-04 |
| *Q.PHM.6.2* | S6.1.25029431 | GG/TT | PHM | 6.13E-04 | 7.76E-04 | 6.13E-04 | 7.61E-04 | 5.78E-04 | 5.78E-04 |
| *Q.PHM.8* | S8.1.13991269 | TT/CT | PHM | 2.00E-05 | 2.00E-05 | 1.00E-05 | 1.00E-05 | 1.00E-05 | 6.08E-06 |
| *Q.PHM.11.1* | S11.1.16313748 | CC/GG | PHM | 1.80E-03 | 6.40E-04 | 9.00E-04 | 6.50E-04 | 5.60E-04 | 8.11E-04 |
| *Q.PHM.11.2* | S11.1.16898133 | TT/CT | PHM | 5.00E-05 | 5.00E-05 | 5.00E-05 | 0.00E+00 | 0.00E+00 | 3.30E-06 |
|  | S11.1.16898169 | AA/AG | PHM | 4.00E-05 | 6.00E-05 | 5.00E-05 | 4.00E-05 | 4.00E-05 | 4.33E-05 |
|  | S11.1.16898170 | AA/AG | PHM | 4.00E-05 | 6.00E-05 | 5.00E-05 | 4.00E-05 | 4.00E-05 | 4.33E-05 |
|  | S11.1.16898225 | GG/AG | PHM | 8.00E-05 | 6.00E-05 | 6.00E-05 | 3.60E-04 | 3.70E-04 | 3.69E-04 |
| *Q.BpP.6* | S6.1.22219189 | AA/AT | BpP | 8.60E-01 | 9.60E-01 | 9.40E-01 | 4.10E-01 | 3.80E-01 | 3.80E-01 |
| *Q.NpP.4* | S4.1.1343019 | CC/CT | NpP | 7.00E-01 | 9.30E-01 | 8.80E-01 | 1.00E-04 | 9.00E-05 | 8.55E-05 |
| *Q.NpP.6* | S6.1.32970231 | TT/CT | NpP | 0.00E+00 | 2.00E-05 | 1.00E-05 | 1.40E-02 | 1.40E-02 | 1.35E-02 |
|  | S6.1.32970252 | GG/AG | NpP | 0.00E+00 | 5.00E-05 | 1.00E-05 | 2.00E-02 | 1.20E-02 | 1.16E-02 |
|  | S6.1.32970258 | TT/CT | NpP | 0.00E+00 | 0.00E+00 | 0.00E+00 | 9.70E-02 | 6.50E-02 | 6.54E-02 |
| *Q.IL.1.1* | S1.1.2636313 | CC/CT | IL | 9.10E-04 | 6.40E-04 | 7.40E-04 | 1.40E-04 | 7.00E-05 | 7.22E-05 |
| *Q.IL.1.2* | S1.1.35117546 | CC/CT | IL | 1.00E-04 | 1.80E-04 | 1.30E-04 | 2.70E-01 | 1.90E-01 | 1.93E-01 |
| *Q.IL.5* | S5.1.15033908 | GG/AG | IL | 1.80E-02 | 1.20E-02 | 1.40E-02 | 9.80E-02 | 1.30E-01 | 1.31E-01 |
| *Q.IL.8* | S8.1.13991269 | CC/CT | IL | 1.00E-04 | 7.00E-05 | 8.00E-05 | 1.30E-03 | 5.10E-04 | 5.10E-04 |
| *Q.CpP.1* | S1.1.25545554 | AA/TT | CpP | 1.00E+00 | 1.00E+00 | 1.00E+00 | 0.00E+00 | 0.00E+00 | 1.95E-06 |
| *Q.CpP.4* | S4.1.774872 | TT/CT | CpP | 6.00E-01 | 5.10E-01 | 5.50E-01 | 9.60E-02 | 1.10E-01 | 1.10E-01 |
| *Q.CpP.7* | S7.1.36781633 | TT/AT | CpP | 1.00E-04 | 2.00E-05 | 4.00E-05 | 7.30E-01 | 9.70E-01 | 9.68E-01 |
| *Q.CpP.9* | S9.1.9823250 | AA/AG | CpP | 1.00E-04 | 1.80E-04 | 1.20E-04 | 5.80E-01 | 5.60E-01 | 5.55E-01 |
| *Q.CpP.10* | S10.1.1346198 | GG/GT | CpP | 3.90E-04 | 3.50E-04 | 3.30E-04 | 9.70E-01 | 8.30E-01 | 8.30E-01 |
|  | S10.1.1346205 | AA/AG | CpP | 7.00E-05 | 1.00E-05 | 3.00E-05 | 8.90E-01 | 9.40E-01 | 9.37E-01 |
|  | S10.1.1346249 | AA/AG | CpP | 5.90E-04 | 3.50E-04 | 4.10E-04 | 5.80E-01 | 6.30E-01 | 6.33E-01 |
| *Q.PL.1* | S1.1.30381382 | TT/CT | PL | 9.50E-02 | 1.50E-01 | 1.20E-01 | 2.60E-01 | 2.20E-01 | 2.23E-01 |
| *Q.SpP.3* | S3.1.8767931 | TT/CT | SpP | 1.80E-04 | 1.90E-04 | 7.00E-05 | 1.80E-01 | 1.20E-01 | 1.23E-01 |
| *Q.SpP.6* | S6.1.29235889 | GG/CG | SpP | 2.00E-01 | 2.70E-01 | 1.80E-01 | 2.00E-02 | 2.10E-02 | 2.11E-02 |
| *Q.SpP.8.1* | S8.1.13990816 | TT/CT | SpP | 5.90E-02 | 6.60E-02 | 8.40E-02 | 6.50E-01 | 6.40E-01 | 6.41E-01 |
| *Q.SpP.8.2* | S8.1.16767849 | GG/AG | SpP | 1.40E-02 | 2.80E-02 | 1.80E-02 | 1.70E-03 | 3.00E-03 | 3.03E-03 |
| *Q.BYpP.8* | S8.1.33339056 | AA/AG | BYpP | 5.50E-01 | 5.00E-01 | 5.80E-01 | 7.30E-03 | 9.10E-03 | 9.07E-03 |
| *Q.BYpP.9* | S9.1.5335251 | GG/TT | BYpP | 1.00E+00 | 1.00E+00 | 1.00E+00 | 7.40E-01 | 4.30E-01 | 2.37E-01 |
| *Q.YpP.4.1* | S4.1.9615705 | AA/AT | YpP | 1.20E-03 | 1.80E-03 | 1.30E-03 | 6.00E-01 | 5.50E-01 | 5.53E-01 |
| *Q.YpP.4.2* | S4.1.11912711 | TT/CT | YpP | 6.90E-01 | 7.50E-01 | 7.40E-01 | 2.90E-04 | 8.00E-04 | 7.98E-04 |
| *Q.YpP.5* | S5.1.35989113 | AA/AT | YpP | 6.50E-02 | 7.30E-02 | 6.60E-02 | 1.00E-03 | 6.40E-04 | 6.38E-04 |
| *Q.YpP.8* | S8.1.19533014 | TT/CT | YpP | 1.10E-03 | 1.10E-03 | 1.20E-03 | 3.40E-01 | 4.00E-01 | 4.05E-01 |
| *Q.HI.1* | S1.1.8549788 | GG/AG | HI | 1.20E-03 | 3.40E-04 | 5.80E-04 | 3.50E-01 | 2.00E-01 | 1.99E-01 |
| *Q.HI.5* | S5.1.29623709 | GG/AG | HI | 6.00E-01 | 6.70E-01 | 6.40E-01 | 1.20E-03 | 4.70E-04 | 4.74E-04 |
| *Q.HI.6* | S6.1.16902154 | CC/CG | HI | 9.10E-01 | 9.20E-01 | 9.00E-01 | 1.50E-03 | 3.80E-04 | 3.76E-04 |
| *Q.HI.7* | S7.1.49416375 | GG/AG | HI | 3.14E-01 | 3.82E-01 | 3.55E-01 | 2.74E-04 | 1.14E-04 | 1.14E-04 |
| *Q.HI.8.1* | S8.1.4181215 | GG/AG | HI | 2.50E-01 | 2.40E-01 | 2.50E-01 | 8.80E-03 | 7.10E-03 | 7.06E-03 |
| *Q.HI.8.2* | S8.1.19533014 | TT/CT | HI | 8.90E-04 | 4.50E-04 | 5.60E-04 | 8.70E-01 | 9.40E-01 | 9.36E-01 |
| *Q.HI.10* | S10.1.2365261 | AA/AC | HI | 7.10E-01 | 7.60E-01 | 7.10E-01 | 8.00E-04 | 1.90E-03 | 1.89E-03 |
| *Q.HSW.6* | S6.1.8707985 | CC/CT | HSW | 8.90E-04 | 1.10E-03 | 8.90E-04 | 5.00E-01 | 6.70E-01 | 6.71E-01 |
| *Q.HSW.7* | S7.1.20599131 | CC/CT | HSW | 1.80E-01 | 2.00E-01 | 1.90E-01 | 6.40E-04 | 1.10E-03 | 1.10E-03 |
| *Q.HSW.10* | S10.1.20952550 | AA/AG | HSW | 4.20E-04 | 6.90E-04 | 4.90E-04 | 6.50E-01 | 5.00E-01 | 4.97E-01 |

**Table S8. Superior and Inferior alleles along with mean phenotypic values of different MTAs associated with characters**

| MTA | SNP | Env | | Alleles | SA | IA | SAC (%) | IAC (%) | L | | | G | | |
| --- | --- | --- | --- | --- | --- | --- | --- | --- | --- | --- | --- | --- | --- | --- |
|  |  |  |  |  |  |  |  |  | SAM | IAM | P-value | SAM | IAM | P-value |
| *pQ.DtF.10.1* | S10.1.9527186 | L |  | TT/CT | TT | CT | 86 (86%) | 14 (14%) | 41.4 | 43.1 | 1.00E-05 | NA | NA | 7.81E-01 |
| *pQ.PHM.3.1* | S3.1.7993147 | L | G | AA/GG | AA | GG | 7 (8%) | 92 (92%) | 28.5 | 19.2 | 8.00E-05 | 43.2 | 27.2 | 1.49E-04 |
| *pQ.PHM.3.2* | S3.1.8219594 | L |  | AA/GG | AA | GG | 9 (10%) | 90 (90%) | 27.7 | 19 | 4.00E-05 | 39.1 | 27.8 | 5.44E-03 |
| *pQ.PHM.4.1* | S4.1.1259800 | L | G | CC/CT | CT | CC | 13 (13%) | 87 (87%) | 25.9 | 18.9 | 2.00E-05 | 39.5 | 27.2 | 2.41E-05 |
| *pQ.PHM.6.1* | S6.1.23358875 |  | G | CC/CT | CT | CC | 21 (21%) | 79 (79%) | 22.2 | 19.2 | 3.00E-02 | 35.2 | 27.1 | 9.83E-04 |
| *pQ.PHM.6.2* | S6.1.25029431 | L |  | GG/TT | GG | TT | 19 (19%) | 77 (81%) | 24.2 | 18.6 | 6.13E-04 | 36.5 | 26.5 | 5.78E-04 |
| *pQ.PHM.8.1* | S8.1.13991269 | L | G | TT/CT | CT | TT | 11 (11%) | 89 (89%) | 26.7 | 19 | 1.00E-05 | 41.5 | 27.2 | 6.08E-06 |
| *pQ.PHM.11.1* | S11.1.16313748 |  | G | CC/GG | GG | CC | 11 (11%) | 71 (89%) | 26.5 | 19.1 | 9.00E-04 | 41.3 | 27.5 | 8.11E-04 |
| *pQ.PHM.11.2* | S11.1.16898133 | L |  | TT/CT | CT | TT | 31 (31%) | 66 (69%) | 23.4 | 18.4 | 5.00E-05 | 36.1 | 25.9 | 3.30E-06 |
|  | S11.1.16898169 | L |  | AA/AG | AG | AA | 30 (30%) | 67 (70%) | 23.4 | 18.4 | 5.00E-05 | 35.5 | 26.3 | 4.33E-05 |
|  | S11.1.16898170 | L |  | AA/AG | AG | AA | 30 (30%) | 67 (70%) | 23.4 | 18.4 | 5.00E-05 | 35.5 | 26.3 | 4.33E-05 |
|  | S11.1.16898225 | L |  | GG/AG | AG | GG | 28 (29%) | 71 (71%) | 23.5 | 18.4 | 6.00E-05 | 34.7 | 26.6 | 3.69E-04 |
| *pQ.NpP.4.1* | S4.1.1343019 |  | G | CC/CT | CT | CC | 13 (13%) | 87 (87%) | NA | NA | 8.80E-01 | 14.9 | 12.3 | 8.55E-05 |
| *pQ.NpP.6.1* | S6.1.32970231 | L |  | TT/CT | CT | TT | 19 (19%) | 81 (81%) | 11.4 | 9.37 | 1.00E-05 | 13.8 | 12.3 | 1.35E-02 |
|  | S6.1.32970252 | L |  | GG/AG | AG | GG | 11 (11%) | 89 (89%) | 12 | 9.48 | 1.00E-05 | 14.2 | 12.4 | 1.16E-02 |
|  | S6.1.32970258 | L |  | TT/CT | CT | TT | 17 (17%) | 83 (83%) | 11.7 | 9.37 | 0.00E+00 | NA | NA | 6.54E-02 |
| *pQ.IL.1.1* | S1.1.2636313 |  | G | CC/CT | CC | CT | 77 (79%) | 21 (21%) | 1.98 | 2.53 | 7.40E-04 | 2.19 | 2.68 | 7.22E-05 |
| *pQ.IL.1.2* | S1.1.35117546 | L |  | CC/CT | CC | CT | 88 (89%) | 11 (11%) | 2.01 | 2.81 | 1.30E-04 | NA | NA | 1.93E-01 |
| *pQ.IL.5.1* | S5.1.15033908 | L |  | GG/AG | GG | AG | 84 (86%) | 14 (14%) | 2.01 | 2.35 | 1.40E-02 | NA | NA | 1.31E-01 |
| *pQ.IL.8.1* | S8.1.13991269 | L |  | CC/CT | TT | CT | 89 (89%) | 11 (11%) | 2.01 | 2.83 | 8.00E-05 | 2.24 | 2.8 | 5.10E-04 |
| *pQ.CpP.1.2* | S1.1.25545554 |  | G | AA/TT | AA | TT | 7 (7%) | 93 (93%) | NA | NA | 1.00E+00 | 18.8 | 12.7 | 1.95E-06 |
| *pQ.CpP.4.1* | S4.1.1341505 |  | G | GG/AG | GG | AG | 85 (85%) | 15 (15%) | NA | NA | 6.50E-01 | 13.6 | 11.6 | 2.70E-02 |
| *pQ.CpP.7.1* | S7.1.36781633 | L |  | TT/AT | AT | TT | 12 (12%) | 88 (88%) | 10.9 | 8.8 | 4.00E-05 | NA | NA | 9.68E-01 |
| *pQ.CpP.9.1* | S9.1.9823250 | L |  | AA/AG | AG | AA | 12 (12%) | 88 (88%) | 10.8 | 8.82 | 1.20E-04 | NA | NA | 5.55E-01 |
| *pQ.CpP.10.1* | S10.1.1346198 | L |  | GG/GT | GT | GG | 18 (18%) | 82 (82%) | 10.3 | 8.77 | 3.30E-04 | NA | NA | 8.30E-01 |
|  | S10.1.1346205 | L |  | AA/AG | AG | AA | 11 (11%) | 89 (89%) | 11 | 8.81 | 3.00E-05 | NA | NA | 9.37E-01 |
|  | S10.1.1346249 | L |  | AA/AG | AG | AA | 19 (19%) | 81 (81%) | 10.2 | 8.77 | 4.10E-04 | NA | NA | 6.33E-01 |
| *pQ.SpP.3.1* | S3.1.8767931 | L |  | TT/CT | CT | TT | 11 (12%) | 88 (88%) | 6.67 | 6.18 | 7.00E-05 | NA | NA | 1.23E-01 |
| *pQ.SpP.6.1* | S6.1.29235836 |  | G | CC/AC | CC | AC | 65 (71%) | 29 (29%) | NA | NA | 9.70E-01 | 6.71 | 6.45 | 1.58E-02 |
|  | S6.1.29235837 |  | G | AA/AG | AA | AG | 65 (71%) | 29 (29%) | NA | NA | 9.70E-01 | 6.71 | 6.45 | 1.58E-02 |
|  | S6.1.29235889 |  | G | GG/CG | GG | CG | 74 (80%) | 20 (20%) | NA | NA | 1.80E-01 | 6.69 | 6.4 | 2.11E-02 |
|  | S6.1.29235890 |  | G | TT/CT | TT | CT | 76 (83%) | 17 (17%) | NA | NA | 1.70E-01 | 6.68 | 6.42 | 5.53E-02 |
| *pQ.SpP.8.2* | S8.1.16767849 |  | G | GG/AG | GG*/AG** | AG*/GG** | 71*/ 29** | 29*/ 71** | 6.31 | 6.1 | 1.80E-02 | 6.51 | 6.82 | 3.03E-03 |
| *pQ.BYpP.8.1* | S8.1.33339056 |  | G | AA/AG | AG | AA | 28 (29%) | 71 (71%) | NA | NA | 5.80E-01 | 28.1 | 23.8 | 9.07E-03 |
| *pQ.YpP.4.1* | S4.1.9615705 | L |  | AA/AT | AT | AA | 11 (11%) | 89 (89%) | 5.52 | 4.22 | 1.30E-03 | NA | NA | 5.53E-01 |
| *pQ.YpP.4.2* | S4.1.11912711 |  | G | TT/CT | CT | TT | 13 (14%) | 86 (86%) | NA | NA | 7.40E-01 | 6.41 | 5.03 | 7.98E-04 |
| *pQ.YpP.5.1* | S5.1.35989113 |  | G | AA/AT | AA | AT | 84 (85%) | 15 (15%) | NA | NA | 6.60E-02 | 5.47 | 4.21 | 6.38E-04 |
| *pQ.YpP.8.1* | S8.1.19533014 | L |  | TT/CT | TT | CT | 66 (69%) | 31 (31%) | 4.73 | 3.79 | 1.20E-03 | NA | NA | 4.05E-01 |
| *pQ.HSW.6.1* | S6.1.8707985 | L |  | CC/CT | CC | CT | 87 (88%) | 12 (12%) | 4.6 | 4.28 | 8.90E-04 | NA | NA | 6.71E-01 |
| *pQ.HSW.7.1* | S7.1.20599131 |  | G | CC/CT | CT | CC | 15 (15%) | 85 (85%) | NA | NA | 1.90E-01 | 4.79 | 4.54 | 1.10E-03 |
| *pQ.HSW.10.1* | S10.1.20952550 | L |  | AA/AG | AG | AA | 27 (27%) | 73 (73%) | 4.73 | 4.49 | 4.90E-04 | NA | NA | 4.97E-01 |
| *pQ.HI.1.1* | S1.1.8549788 | L |  | GG/AG | GG | AG | 81 (81%) | 19 (19%) | 22.5 | 18.6 | 5.80E-04 | NA | NA | 1.99E-01 |
| *pQ.HI.5.1* | S5.1.29623709 |  | G | GG/AG | GG | AG | 71 (72%) | 28 (28%) | NA | NA | 6.40E-01 | 22.3 | 19.1 | 4.74E-04 |
| *pQ.HI.6.1* | S6.1.16902154 |  | G | CC/CG | CG | CC | 16 (16%) | 84 (84%) | NA | NA | 9.00E-01 | 24.6 | 20.7 | 3.76E-04 |
| *pQ.HI.7.1* | S7.1.49416375 |  | G | GG/AG | GG | GT | 69 (78%) | 22 (22%) | NA | NA | 3.55E-01 | 22.6 | 18.2 | 1.14E-04 |
| *pQ.HI.8.1* | S8.1.4181215 |  | G | GG/AG | AG | GG | 14 (15%) | 85 (85%) | NA | NA | 2.50E-01 | 23.9 | 20.7 | 7.06E-03 |
| *pQ.HI.8.2* | S8.1.19533014 | L |  | TT/CT | TT | CT | 66 (69%) | 31 (31%) | 22.9 | 19.4 | 5.60E-04 | NA | NA | 9.36E-01 |
| *pQ.HI.10.1* | S10.1.2365261 |  | G | AA/AC | AA | AC | 83 (83%) | 17 (17%) | NA | NA | 7.10E-01 | 22 | 18.3 | 1.89E-03 |

L- Ludhiana, G - Gurdaspur *- superior/inferior in E3, **- superior/inferior in E6

**Table S9: Summary of the high performing genotypes along with QTL composition of these genotypes**

| Genotype | QTLs present |
| --- | --- |
| IC328783 | *pQ.PHM.11.1 ; pQ.PHM.3.1 ; pQ.PHM.3.2 ; pQ.PHM.4.1 ; pQ.PHM.6.1 ; pQ.PHM.6.2 ; pQ.PHM.8.1 ; pQ.NpP.4.1 ; pQ.NpP.6.1 ; pQ.CpP.10.1 ; pQ.CpP.7.1 ; pQ.CpP.9.1* |
| MASH218 | *pQ.PHM.3.2 ; pQ.CpP.10.1 ; pQ.CpP.9.1 ; pQ.HI.1.1 ; pQ.HI.10.1 ; pQ.HI.5.1 ; pQ.HI.8.2 ; pQ.YpP.4.1 ; pQ.YpP.5.1 ; pQ.YpP.8.1* |
| IC370938 | *pQ.PpP.8.1 ; pQ.YpP.4.2 ; pQ.YpP.8.1 ; pQ.HSW.10.1 ; pQ.HSW.6.1 ; pQ.HSW.7.1 ; pQ.HI.1.1 ; pQ.HI.10.1 ; pQ.HI.5.1 ; pQ.HI.7.1 ; pQ.HI.8.2* |
| IC274597 | *pQ.CpP.1.2 ; pQ.CpP.10.1 ; pQ.SpP.6.1 ; pQ.SpP.8.2 ; pQ.BYpP.8.1* |
| KUG673 | *pQ.DtF.10.1 ;pQ.HI.1.1 ; pQ.HI.10.1 ; pQ.HI.5.1 ; pQ.HI.6.1 ; pQ.HI.7.1* |

**Table S10: List of candidate genes with KEGG pathway and Enzyme annotation**

| QTL | Trait | Gene ID | Dist (Kb) | function | Pathway | | | Enzyme code | Enzyme name | | | |
| --- | --- | --- | --- | --- | --- | --- | --- | --- | --- | --- | --- | --- |
| *Q.DtF.10* | DtF | *LOC106774489* | 73.878 | PHD finger-like domain-containing protein 5B | Transcription (Spliceosome) | | |  |  | | | |
| *Q.PHM.3.1* | PHM | *LOC106757287* | -100.237 | E3 ubiquitin-protein ligase MARCH1 |  | | | EC:2.7.3; EC:2.7.13.3; EC:6 | Transferring phosphorus-containing groups; Histidine kinase; Ligases | | | |
|  |  | *LOC106757069* | -65.973 | bZIP transcription factor 53 |  | | | EC:2.7.11.24 | Mitogen-activated protein kinase | | | |
|  |  | *LOC106757136* | -47.55 | protein trichome birefringence-like 6 |  | | | EC:2.3.1 | Acyltransferases | | | |
|  |  | *LOC106756978* | -43.026 | histone-lysine N-methyltransferase EZ2-like | Lysine degradation (Amino acid metabolism) | | | EC:2.1.1.356 |  | | | |
|  |  | *LOC111241394* | -33.113 | DELLA protein RGL1-like |  | | |  |  | | | |
|  |  | *LOC106757804* | -2.328 | DEAD-box ATP-dependent RNA helicase 24 | Transcription (Spliceosome) | | | EC:3.6.4.13 | RNA helicase | | | |
|  |  | *LOC106757666* | 50.731 | probable WRKY transcription factor 23 |  | | |  |  | | | |
|  |  | *LOC106756983* | 98.775 | gibberellin 2-beta-dioxygenase 2 | Diterpenoid biosynthesis | | | EC:1.14.11.13 | Gibberellin 2-beta-dioxygenase | | | |
|  |  | *LOC106756984* | 118.512 | transcription factor JAMYB-like |  | | |  |  | | | |
| *Q.PHM.4* | PHM | *LOC106759452* | -178.201 | tropinone reductase homolog | Tropane, piperidine and pyridine alkaloid biosynthesis | | | EC:1 | Oxidoreductases | | | |
|  |  | *LOC106758588* | -69.349 | tropinone reductase homolog At5g06060 |  | | | EC:2.7.7 | Transferring phosphorus-containing groups | | | |
| *Q.PHM.6.2* | PHM | *LOC106764341* | -82.244 | steroid 5-alpha-reductase DET2 | Brassinosteroid biosynthesis, Steroid degradation, Steroid hormone biosynthesis | | | EC:1.3.99.5; EC:1.3.1.22 | 3-oxo-5-alpha-steroid 4-dehydrogenase (acceptor); 3-oxo-5-alpha-steroid 4-dehydrogenase (NADP(+)) | | | |
| *Q.PHM.11.1* | PHM | *LOC106777611* | -171.802 | squamosa promoter-binding-like protein 14 |  | | |  |  | | | |
|  |  | *LOC106777237* | -112.535 | cytochrome P450 71D11 |  | | | EC:1.14 | Acting on paired donors, with incorporation or reduction of molecular oxygen. The oxygen incorporated need not be derived from O(2) | | | |
|  |  | *LOC106777539* | -82.108 | pentatricopeptide repeat-containing protein At3g48810 |  | | | EC:3.4 | Acting on peptide bonds (peptidases) | | | |
| *Q.IL.1.1* | IL | *LOC106766854* | -33.322 | pectate lyase-like | Pentose and glucuronate interconversions, Quorum sensing | | | EC:4.2.2.2 | Pectate lyase | | | |
|  |  | *LOC106765724* | -33.083 | pectate lyase | Pentose and glucuronate interconversions, Quorum sensing | | | EC:4.2.2.2 | Pectate lyase | | | |
| *Q.IL.1.2* | IL | *LOC106762425* | -99.425 | cytokinin hydroxylase |  | | | EC:1.14 | Acting on paired donors, with incorporation or reduction of molecular oxygen. The oxygen incorporated need not be derived from O(2) | | | |
| *Q.IL.5* | IL | *LOC106760883* | -189.1 | purine permease 1 |  | | | EC:7 | Translocases | | | |
|  |  | *LOC106762422* | 16.957 | ethylene-responsive transcription factor RAP2-4 |  | | |  |  | | | |
| *Q.CpP.1* | CpP | *LOC106768944* | -108.532 | SKP1-interacting partner 15 |  | | |  |  | | | |
|  |  | *LOC106760064* | -32.774 | receptor-like protein 12 |  | | |  |  | | | |
|  |  | *LOC106760083* | 68.491 | polygalacturonase-like | Pentose and glucuronate interconversions, | | | EC:3.2.1.15 | Endo-polygalacturonase | | | |
| *Q.CpP.7* | CpP | *LOC106765735* | -115.443 | protein POLLEN DEFECTIVE IN GUIDANCE 1 |  | | |  |  | | | |
|  |  | *LOC106766388* | 42.507 | LRR receptor-like serine/threonine-protein kinase RPK2 | Starch and sucrose metabolism | | | EC:2.7.1; EC:2.4.1.12 | Transferring phosphorus-containing groups; Cellulose synthase (UDP-forming) | | | |
| *Q.CpP.9* | CpP | *LOC111242573* | -79.009 | eukaryotic translation initiation factor 3 subunit H-like |  | | | EC:3.4; EC:2 | Acting on peptide bonds (peptidases); Transferases | | | |
|  |  | *LOC106773784* | 166.402 | MLO-like protein 1 |  | | |  |  | | | |
| *Q.CpP.10* | CpP | *LOC106775061* | 131.587 | DDB1- and CUL4-associated factor 13 |  | | |  |  | | | |
| *Q.SpP.3* | SpP | *LOC106757271* | 71.381 | galactinol synthase 2 | Galactose metabolism | | | EC:2.4.1.123 | Inositol 3-alpha-galactosyltransferase | | | |
|  |  | *LOC106757661* | 95.044 | Golgi apparatus membrane protein-like protein ECHIDNA |  | | |  |  | | | |
|  |  | *LOC106756994* | 100.043 | alkaline/neutral invertase A, mitochondrial | Galactose metabolism, Starch and sucrose metabolism | | | EC:3.2.1.20; EC:3.2.1.97; EC:3.2.1.26; EC:3.2.1.48 | Alpha-glucosidase; Endo-alpha-N-acetylgalactosaminidase; Beta-fructofuranosidase; Sucrose alpha-glucosidase | | | |
| *Q.SpP.6* | SpP | *LOC106765120* | 64.962 | dihydrofolate synthetase |  | | | EC:3.1; EC:2.7.7 | Acting on ester bonds; Transferring phosphorus-containing groups | | | |
| *Q.SpP.8.2* | SpP | *LOC106770299* | 147.684 | ethylene-responsive transcription factor 1B-like |  | | |  |  | | | |
| *Q.YpP.4.1* | YpP | *LOC106759105* | 7.311 | myb-related protein 305-like |  | | |  |  | | | |
| *Q.YpP.5* | YpP | *LOC106761836* | -177.436 | CLAVATA3/ESR (CLE)-related protein 5-like |  | | |  |  | | | |
|  |  | *LOC106760678* | -107.67 | transcription factor PCF5 |  | | |  |  | | | |
|  |  | *LOC106762074* | -50.026 | sodium/calcium exchanger NCL |  | | | EC:7 | Translocases | | | |
|  |  | *LOC106759995* | 141.103 | basic leucine zipper 34 isoform X1 |  | | |  |  | | | |
| *Q.YpP.8* | YpP | *LOC111242272* | 116.438 | alpha-mannosidase-like | Other glycan degradation | | | EC:3.2.1.24 | Alpha-mannosidase | | | |
|  |  | *LOC106771274* | 129.392 | putative 12-oxophytodienoate reductase 11 |  | | | EC:1 | Oxidoreductases | | | |
| *Q.HI.1* | HI | *LOC106758323* | -174.544 | UV-B-induced protein, chloroplastic isoform X1 |  | | |  |  | | | |
| *Q.HI.5* | HI | *LOC106760579* | -189.197 | cytochrome P450 CYP72A219-like | Diterpenoid biosynthesis, Steroid hormone biosynthesis | | | EC:1.14 | Acting on paired donors, with incorporation or reduction of molecular oxygen. The oxygen incorporated need not be derived from O(2) | | | |
| *Q.HI.7* | HI | *LOC106769438* | 105.344 | protein root UVB sensitive 6 |  | | | EC:2 | Transferases | | | |
| *Q.HI.8.2* | HI | *LOC111242272* | 116.438 | alpha-mannosidase-like | Other glycan degradation | | | EC:3.2.1.24 | Alpha-mannosidase | | | |
|  |  | *LOC106771274* | 129.392 | putative 12-oxophytodienoate reductase 11 |  | | | EC:1 | Oxidoreductases | | | |
| *Q.HSW.6* | HSW | *LOC106764301* | -15.52 | putative pentatricopeptide repeat-containing protein At1g12700, mitochondrial isoform X1 |  | | | EC:3.1 | Acting on ester bonds | | | |
|  |  | *LOC106765194* | 12.233 | peroxidase 4 | Phenylpropanoid biosynthesis | | | EC:1.11.1 | Acting on a peroxide as acceptor | | | |
| *QTL* | Trait | *Gene ID* | Dist (Kb) | function | Pathway | | | Enzyme code | Enzyme name | | | |
| *Q.DtF.10* | DtF | *LOC106774489* | 73.878 | PHD finger-like domain-containing protein 5B | Transcription (Spliceosome) | |  |  |  |  |  |  |
| *Q.PHM.3.1* | PHM | *LOC106757287* | -100.237 | E3 ubiquitin-protein ligase MARCH1 | | EC:2.7.3; EC:2.7.13.3; EC:6 | | | | Transferring phosphorus-containing groups; Histidine kinase; Ligases | |  |
|  |  | *LOC106757069* | -65.973 | bZIP transcription factor 53 | | EC:2.7.11.24 | | | | Mitogen-activated protein kinase | |  |
|  |  | *LOC106757136* | -47.55 | protein trichome birefringence-like 6 | | EC:2.3.1 | | | | Acyltransferases | |  |
|  |  | *LOC106756978* | -43.026 | histone-lysine N-methyltransferase EZ2-like | Lysine degradation (Amino acid metabolism) | | | EC:2.1.1.356 | | |  |  |
|  |  | *LOC111241394* | -33.113 | DELLA protein RGL1-like | | | | | |  | |  |
|  |  | *LOC106757804* | -2.328 | DEAD-box ATP-dependent RNA helicase 24 | Transcription (Spliceosome) | | | EC:3.6.4.13 | RNA helicase | | | |
|  |  | *LOC106757666* | 50.731 | probable WRKY transcription factor 23 | | |  |  |  |  |  |  |
|  |  | *LOC106756983* | 98.775 | gibberellin 2-beta-dioxygenase 2 | Diterpenoid biosynthesis | | | EC:1.14.11.13 | Gibberellin 2-beta-dioxygenase | | | |
|  |  | *LOC106756984* | 118.512 | transcription factor JAMYB-like | | |  |  |  |  |  |  |
| *Q.PHM.4* | PHM | *LOC106759452* | -178.201 | tropinone reductase homolog | Tropane, piperidine and pyridine alkaloid biosynthesis | | | EC:1 | Oxidoreductases | | | |
|  |  | *LOC106758588* | -69.349 | tropinone reductase homolog At5g06060 | | EC:2.7.7 | | | | Transferring phosphorus-containing groups | |  |
| *Q.PHM.6.2* | PHM | *LOC106764341* | -82.244 | steroid 5-alpha-reductase DET2 | Brassinosteroid biosynthesis, Steroid degradation, Steroid hormone biosynthesis | | | EC:1.3.99.5; EC:1.3.1.22 | 3-oxo-5-alpha-steroid 4-dehydrogenase (acceptor); 3-oxo-5-alpha-steroid 4-dehydrogenase (NADP(+)) | | | |
| *Q.PHM.11.1* | PHM | *LOC106777611* | -171.802 | squamosa promoter-binding-like protein 14 | | |  |  |  |  |  |  |
|  |  | *LOC106777237* | -112.535 | cytochrome P450 71D11 | | EC:1.14 | | | | Acting on paired donors, with incorporation or reduction of molecular oxygen. The oxygen incorporated need not be derived from O(2) | |  |
|  |  | *LOC106777539* | -82.108 | pentatricopeptide repeat-containing protein At3g48810 | | EC:3.4 | | | | Acting on peptide bonds (peptidases) | |  |
| *Q.IL.1.1* | IL | *LOC106766854* | -33.322 | pectate lyase-like | Pentose and glucuronate interconversions, Quorum sensing | | | EC:4.2.2.2 | Pectate lyase | | | |
|  |  | *LOC106765724* | -33.083 | pectate lyase | Pentose and glucuronate interconversions, Quorum sensing | | | EC:4.2.2.2 | Pectate lyase | | | |
| *Q.IL.1.2* | IL | *LOC106762425* | -99.425 | cytokinin hydroxylase | | EC:1.14 | | | | Acting on paired donors, with incorporation or reduction of molecular oxygen. The oxygen incorporated need not be derived from O(2) | |  |
| *Q.IL.5* | IL | *LOC106760883* | -189.1 | purine permease 1 | | EC:7 | | | | Translocases | |  |
|  |  | *LOC106762422* | 16.957 | ethylene-responsive transcription factor RAP2-4 | | |  |  |  |  |  |  |
| *Q.CpP.1* | CpP | *LOC106768944* | -108.532 | SKP1-interacting partner 15 | | | | | |  | |  |
|  |  | *LOC106760064* | -32.774 | receptor-like protein 12 | | | | | |  | |  |
|  |  | *LOC106760083* | 68.491 | polygalacturonase-like | Pentose and glucuronate interconversions, | | | EC:3.2.1.15 | Endo-polygalacturonase | | | |
| *Q.CpP.7* | CpP | *LOC106765735* | -115.443 | protein POLLEN DEFECTIVE IN GUIDANCE 1 | | |  |  |  |  |  |  |
|  |  | *LOC106766388* | 42.507 | LRR receptor-like serine/threonine-protein kinase RPK2 | Starch and sucrose metabolism | | | EC:2.7.1; EC:2.4.1.12 | Transferring phosphorus-containing groups; Cellulose synthase (UDP-forming) | | | |
| *Q.CpP.9* | CpP | *LOC111242573* | -79.009 | eukaryotic translation initiation factor 3 subunit H-like | | EC:3.4; EC:2 | | | | Acting on peptide bonds (peptidases); Transferases | |  |
|  |  | *LOC106773784* | 166.402 | MLO-like protein 1 | |  | | | |  | |  |
| *Q.CpP.10* | CpP | *LOC106775061* | 131.587 | DDB1- and CUL4-associated factor 13 | | |  |  |  |  |  |  |
| *Q.SpP.3* | SpP | *LOC106757271* | 71.381 | galactinol synthase 2 | Galactose metabolism | | | EC:2.4.1.123 | Inositol 3-alpha-galactosyltransferase | | | |
|  |  | *LOC106757661* | 95.044 | Golgi apparatus membrane protein-like protein ECHIDNA | | |  |  |  |  |  |  |
|  |  | *LOC106756994* | 100.043 | alkaline/neutral invertase A, mitochondrial | Galactose metabolism, Starch and sucrose metabolism | | | EC:3.2.1.20; EC:3.2.1.97; EC:3.2.1.26; EC:3.2.1.48 | Alpha-glucosidase; Endo-alpha-N-acetylgalactosaminidase; Beta-fructofuranosidase; Sucrose alpha-glucosidase | | | |
| *Q.SpP.6* | SpP | *LOC106765120* | 64.962 | dihydrofolate synthetase | | EC:3.1; EC:2.7.7 | | | | Acting on ester bonds; Transferring phosphorus-containing groups | |  |
| *Q.SpP.8.2* | SpP | *LOC106770299* | 147.684 | ethylene-responsive transcription factor 1B-like | | |  |  |  |  |  |  |
| *Q.YpP.4.1* | YpP | *LOC106759105* | 7.311 | myb-related protein 305-like | | | | | |  | |  |
| *Q.YpP.5* | YpP | *LOC106761836* | -177.436 | CLAVATA3/ESR (CLE)-related protein 5-like | | |  |  |  |  |  |  |
|  |  | *LOC106760678* | -107.67 | transcription factor PCF5 | | | | | |  | |  |
|  |  | *LOC106762074* | -50.026 | sodium/calcium exchanger NCL | | EC:7 | | | | Translocases | |  |
|  |  | *LOC106759995* | 141.103 | basic leucine zipper 34 isoform X1 | | |  |  |  |  |  |  |
| *Q.YpP.8* | YpP | *LOC111242272* | 116.438 | alpha-mannosidase-like | Other glycan degradation | | | EC:3.2.1.24 | Alpha-mannosidase | | | |
|  |  | *LOC106771274* | 129.392 | putative 12-oxophytodienoate reductase 11 | | EC:1 | | | | Oxidoreductases | |  |
| *Q.HI.1* | HI | *LOC106758323* | -174.544 | UV-B-induced protein, chloroplastic isoform X1 | | |  |  |  |  |  |  |
| *Q.HI.5* | HI | *LOC106760579* | -189.197 | cytochrome P450 CYP72A219-like | Diterpenoid biosynthesis, Steroid hormone biosynthesis | | | EC:1.14 | Acting on paired donors, with incorporation or reduction of molecular oxygen. The oxygen incorporated need not be derived from O(2) | | | |
| *Q.HI.7* | HI | *LOC106769438* | 105.344 | protein root UVB sensitive 6 | | EC:2 | | | | Transferases | |  |
| *Q.HI.8.2* | HI | *LOC111242272* | 116.438 | alpha-mannosidase-like | Other glycan degradation | | | EC:3.2.1.24 | Alpha-mannosidase | | | |
|  |  | *LOC106771274* | 129.392 | putative 12-oxophytodienoate reductase 11 | | EC:1 | | | | Oxidoreductases | |  |
| *Q.HSW.6* | HSW | *LOC106764301* | -15.52 | putative pentatricopeptide repeat-containing protein At1g12700, mitochondrial isoform X1 | | EC:3.1 | | | | Acting on ester bonds | |  |
|  |  | *LOC106765194* | 12.233 | peroxidase 4 | Phenylpropanoid biosynthesis | | | EC:1.11.1 | Acting on a peroxide as acceptor | | | |
| *Q.HSW.10* | HSW | *LOC106776199* | -175.528 | bromodomain-containing  protein 4B | | |  |  |  |  |  |  |

# Distance of 5’ position of the gene from SNP identified associated with the QTL, where – sign shows that the gene was located downstream of the SNP and +sign shows that the gene was located upstream of the SNP.

##Days to 50% flowering (DtF); Plant height at 90% pod maturity (PHM); Internodal length (IL), Clusters per plant (CpP); Seeds per pod (SpP); Yield per plant (YpP); Harvest index (HI) and Hundred seed weight (HSW).
