# Supplementary material for: Genome-wide Association Study for Yield and Yield-Related Traits in Diverse Blackgram Panel (Vigna mungo L. Hepper) Reveals Novel Putative Alleles for Future Breeding Programs
Source: Front Genet. 2022 Jul 11;13:849016. doi: 10.3389/fgene.2022.849016 (PMC9310006; doi:10.3389/fgene.2022.849016)
Supplement: Supplementary file 2 [file Image1.pdf]

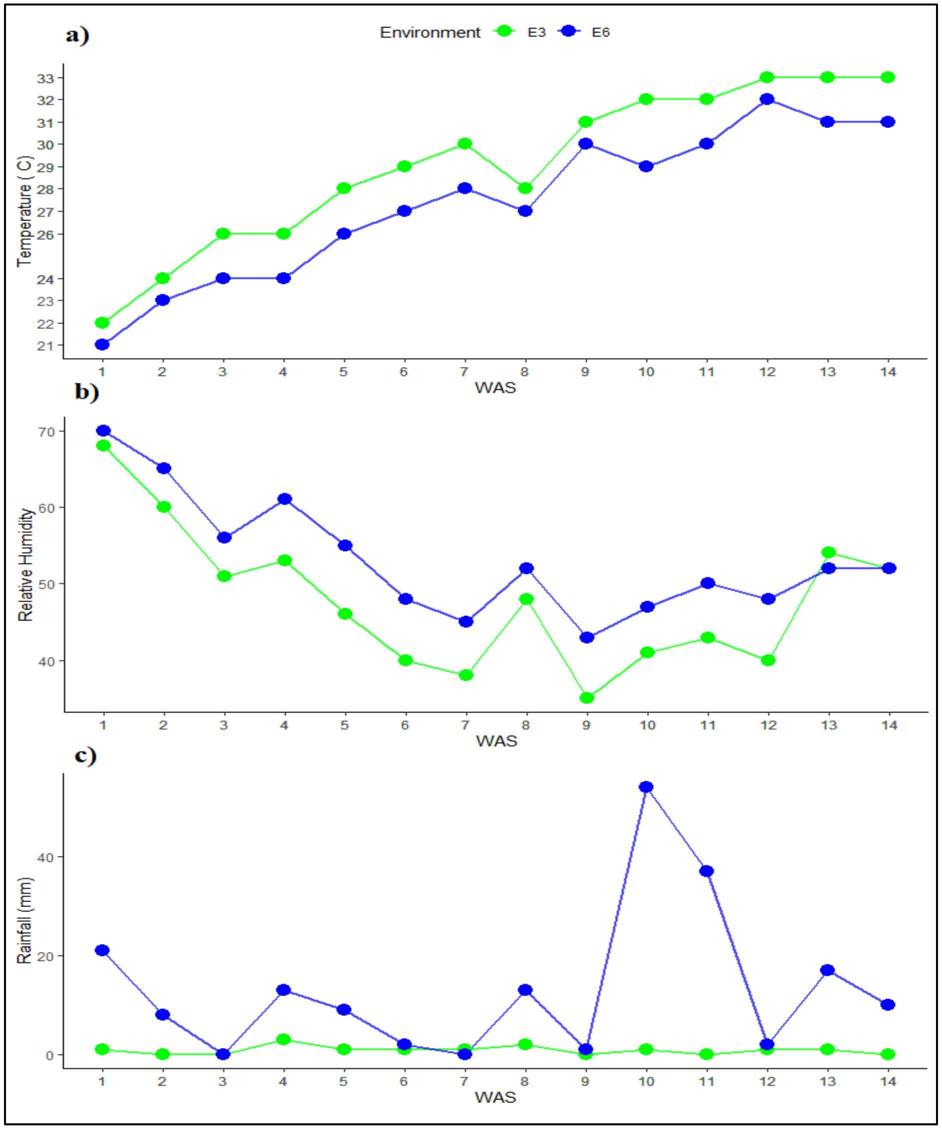

Supplementary Figure S1: Average weekly a) temperature, b) Relative humidity, and c) Rainfall in 1-14 weeks after sowing (WAS), E3 = Ludhiana and E6 = Gurdaspur average over 2019 and 2020.

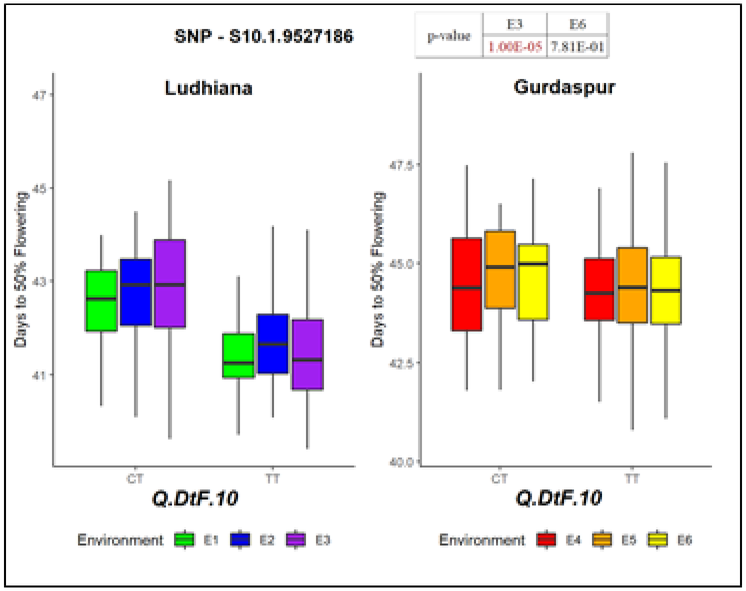

Supplementary Figure S2 (a): Allelic effect of Q_DtF_10-1 on Days to 50% flowering (DtF). (NS) – Non- Significant, (*) – significant at α = 0.05, (**) – significant at α = 0.01, (***) – significant at α = 0.001

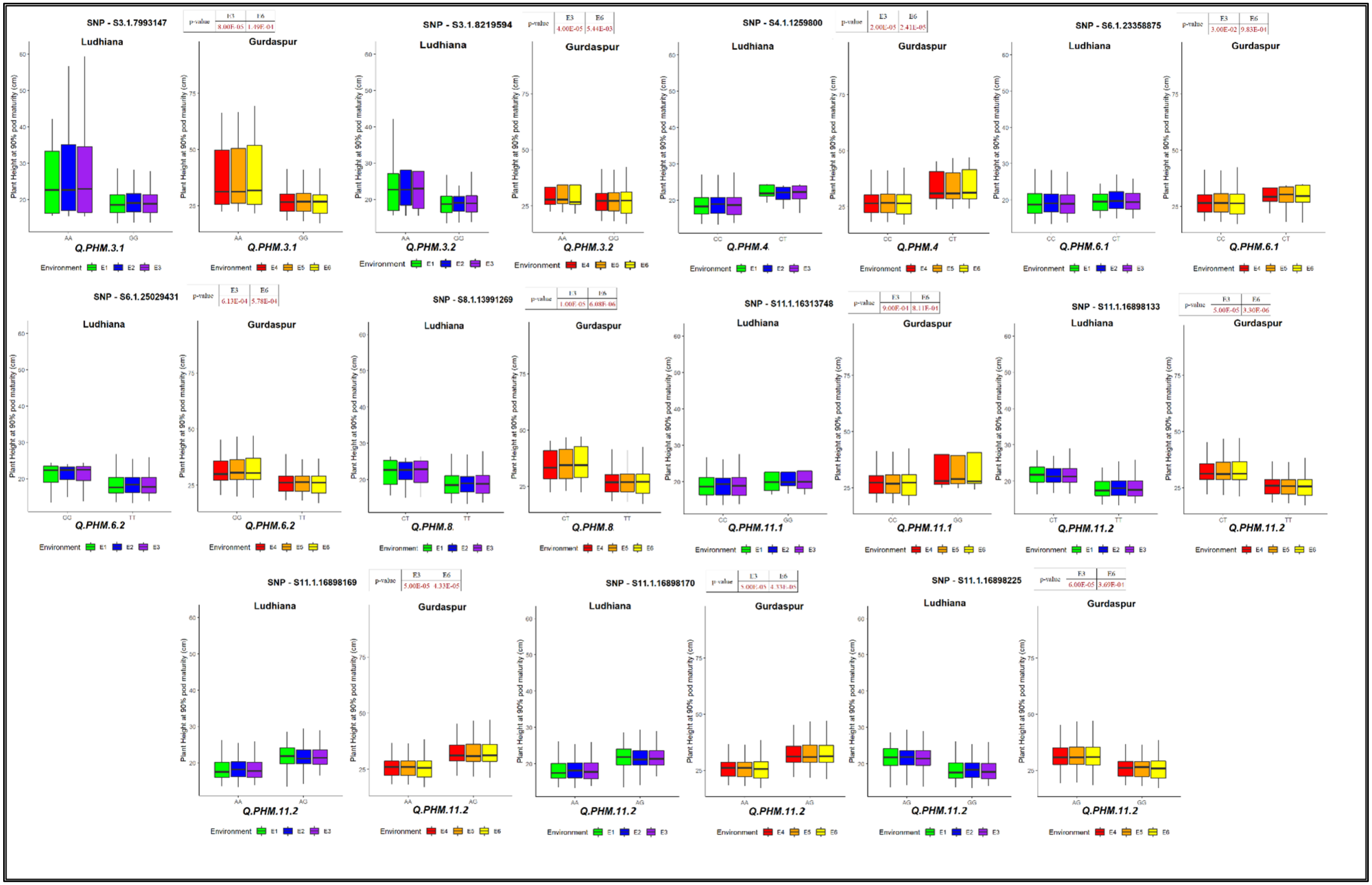

Supplementary Figure S2 (b): Allelic effect of different MTAs on Plant Height at 90% pod maturity (PHM). (NS) – Non- Significant, (*) – significant at α = 0.05, (**) – significant at α = 0.01, (***) – significant at α = 0.001

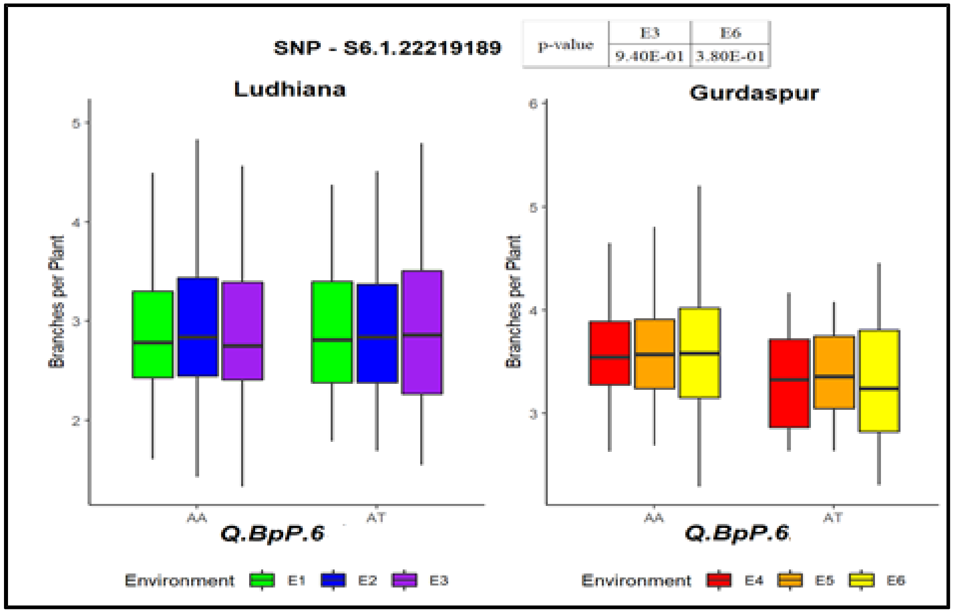

Supplementary Figure S2 (c): Allelic effect of different MTAs on branches per plant (BpP). (NS) – Non- Significant, (*) – significant at α = 0.05, (**) – significant at α = 0.01, (***) – significant at α = 0.001

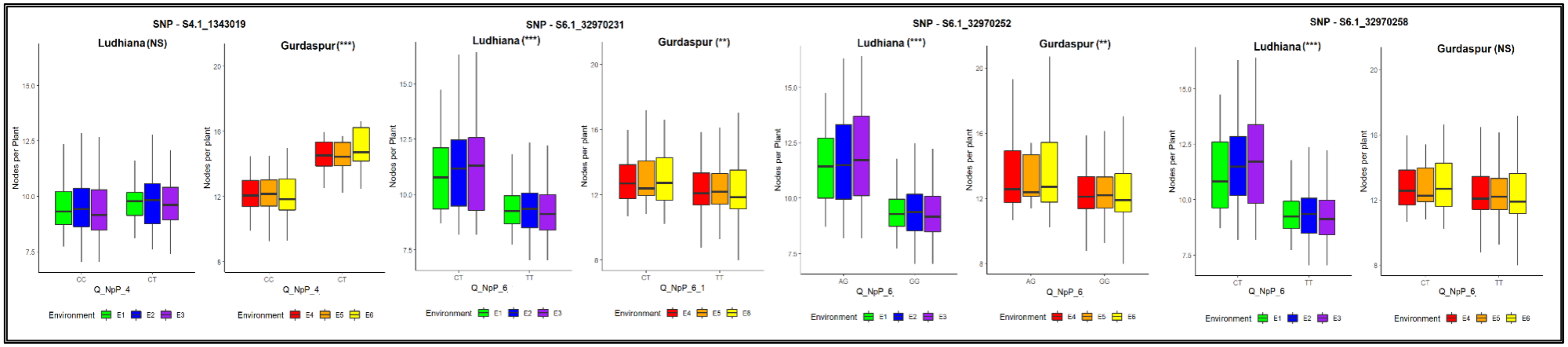

Supplementary Figure S2 (d): Allelic effect of different MTAs on nodes per plant (NpP). (NS) – Non- Significant, (*) – significant at α = 0.05, (**) – significant at α = 0.01, (***) – significant at α = 0.001

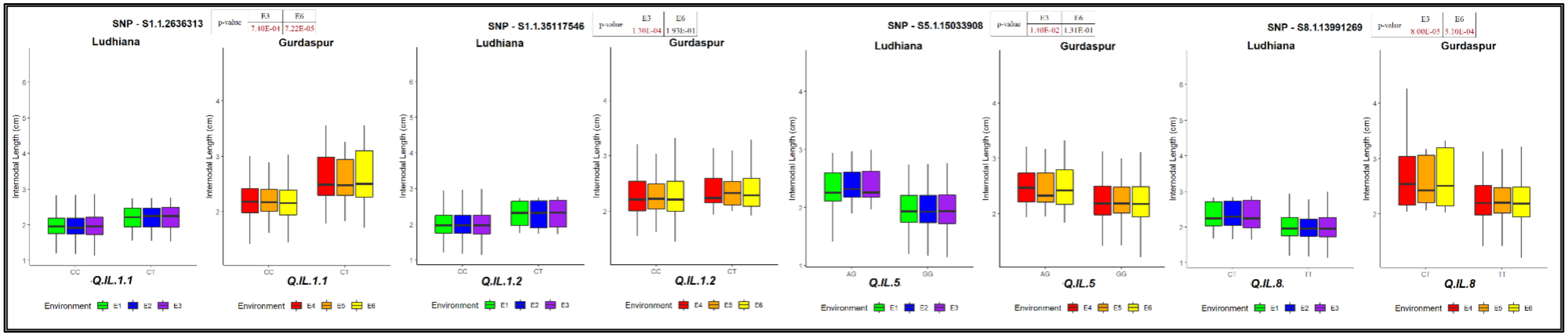

Supplementary Figure S2 (e): Allelic effect of different MTAs on internodal length (IL). (NS) – Non- Significant, (*) – significant at α = 0.05, (**) – significant at α = 0.01, (***) – significant at α = 0.001

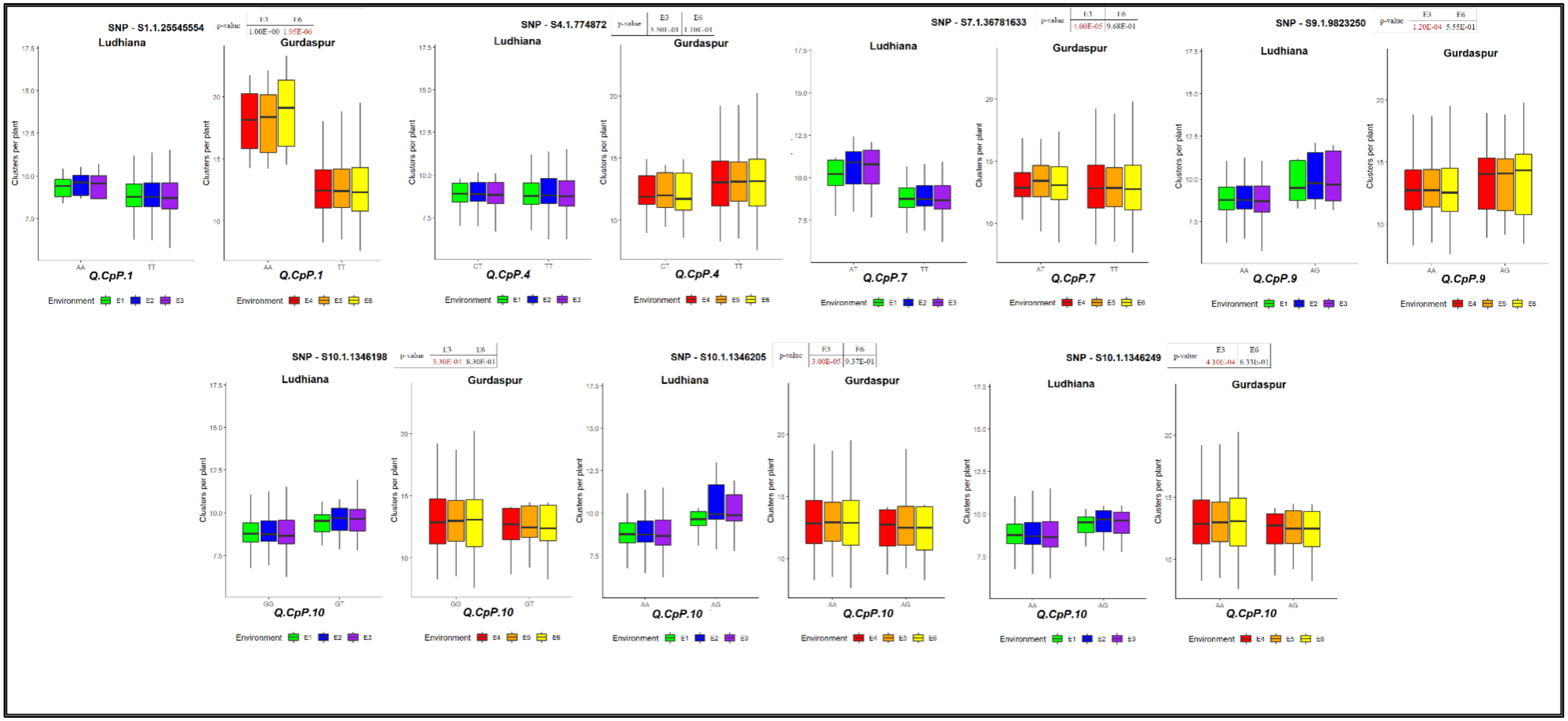

Supplementary Figure S2 (f): Allelic effect of different MTAs on clusters per plant (CpP). (NS) – Non- Significant, (*) – significant at α = 0.05, (**) – significant at α = 0.01, (***) – significant at α = 0.001

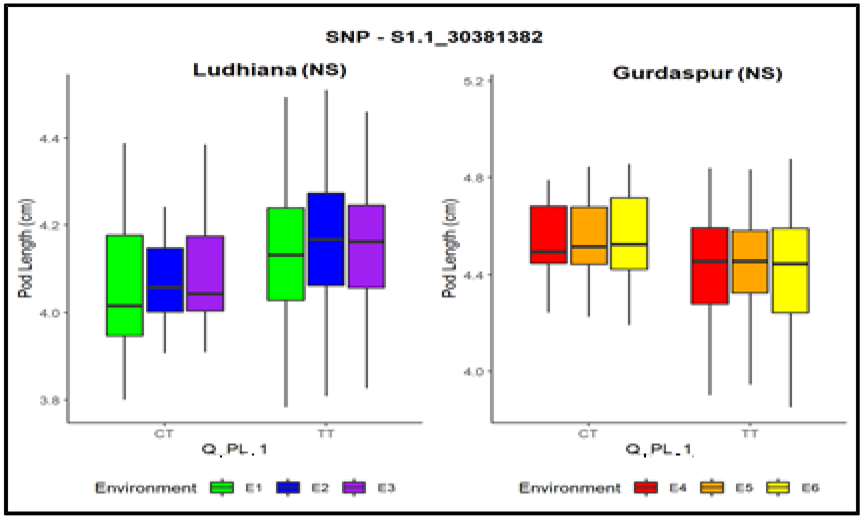

Supplementary Figure S2(g): Allelic effect of different MTAs on pod length (PL). (NS) – Non- Significant, (*) – significant at α = 0.05, (**) – significant at α = 0.01, (***) – significant at α = 0.001

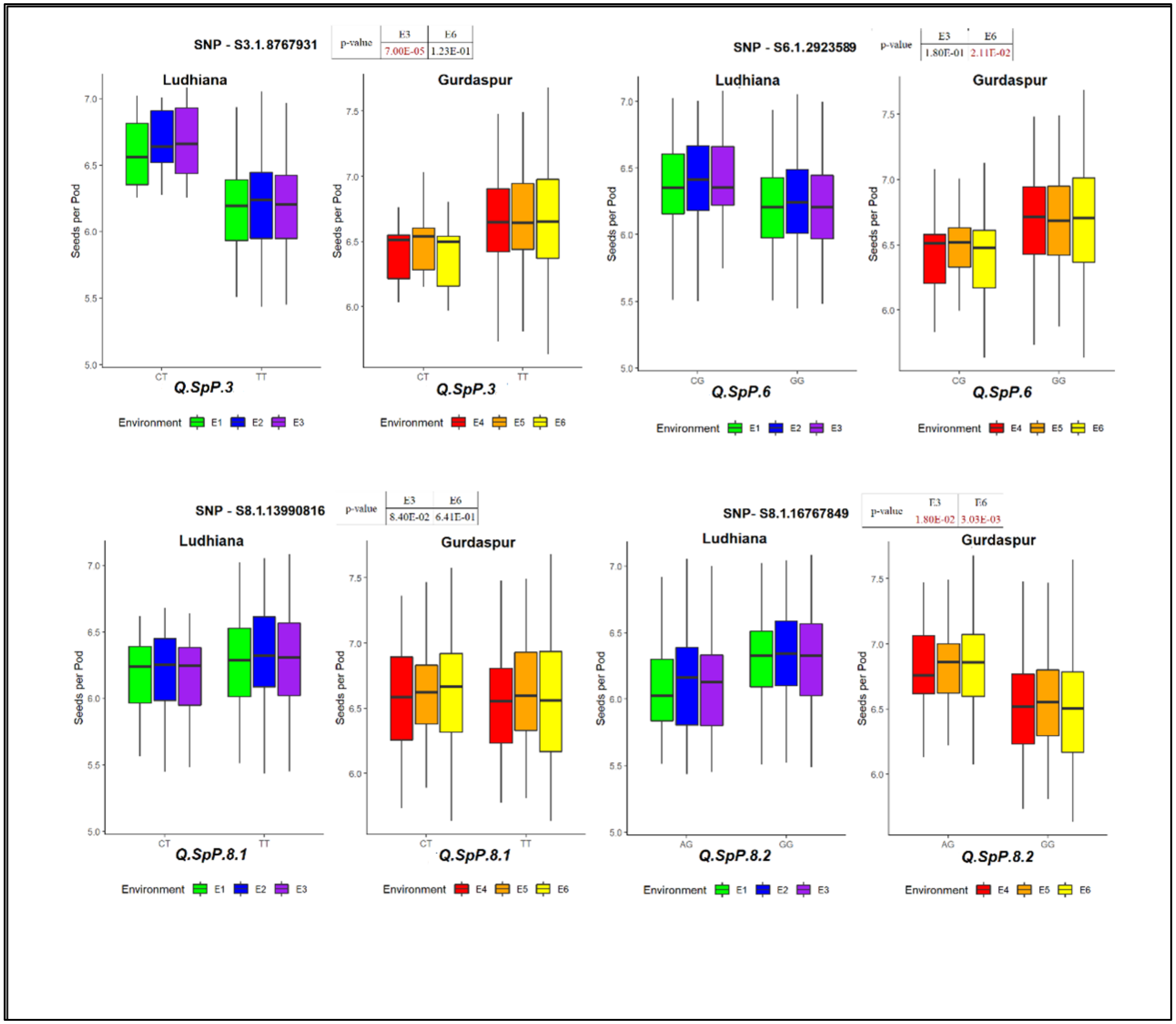

Supplementary Figure S2 (h): Allelic effect of different MTAs seeds per pod (SpP). (NS) – Non- Significant, (*) – significant at α = 0.05, (**) – significant at α = 0.01, (***) – significant at α = 0.001

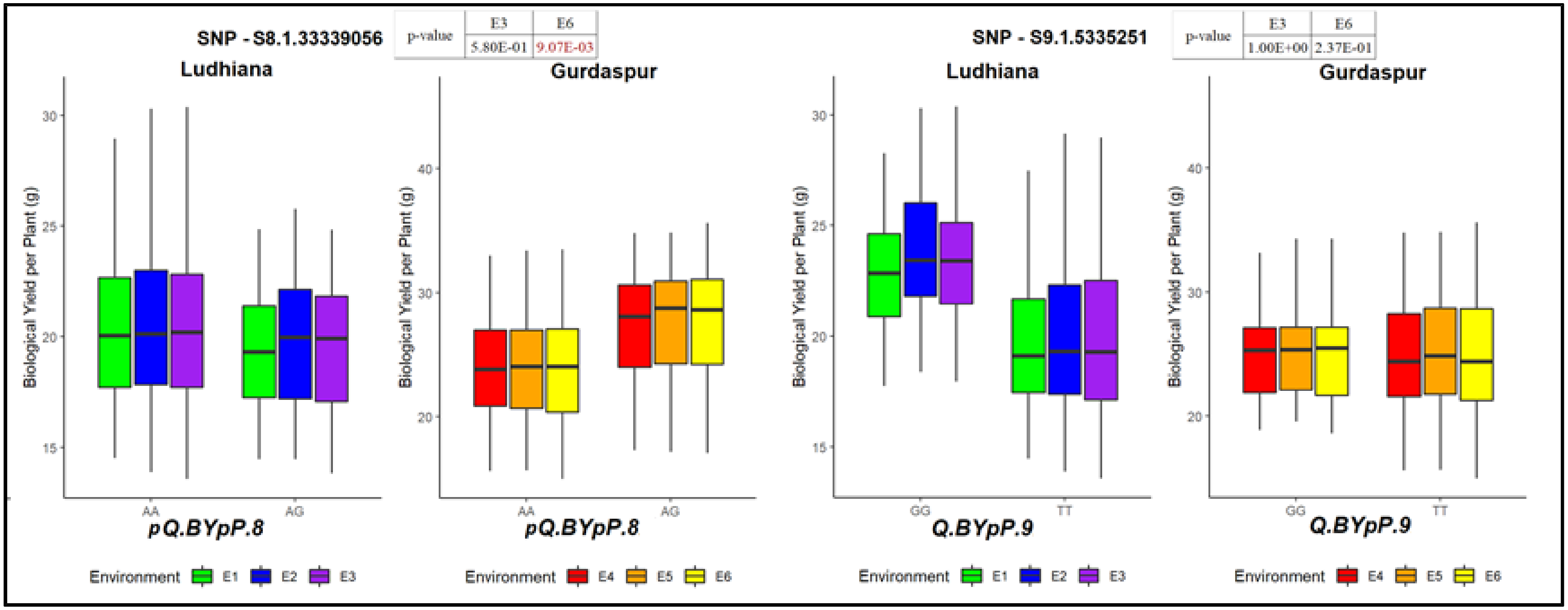

Supplementary Figure S2 (i): Allelic effect of different MTAs on biological yield per plant (BYpP). (NS) – Non- Significant, (*) – significant at α = 0.05, (**) – significant at α = 0.01, (***) – significant at α = 0.001

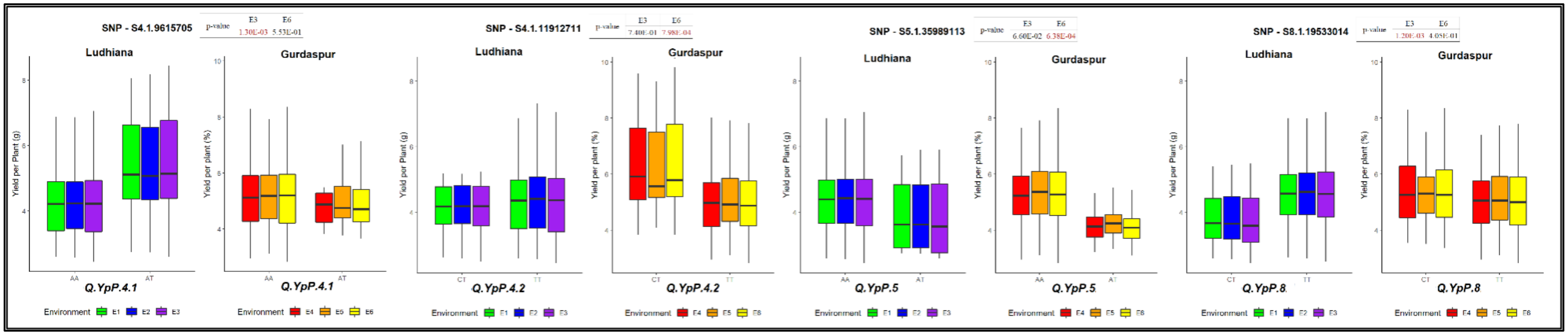

Supplementary Figure S2 (j): Allelic effect of different MTAs on yield per plant (YpP). (NS) – Non- Significant, (*) – significant at α = 0.05, (**) – significant at α = 0.01, (***) – significant at α = 0.001

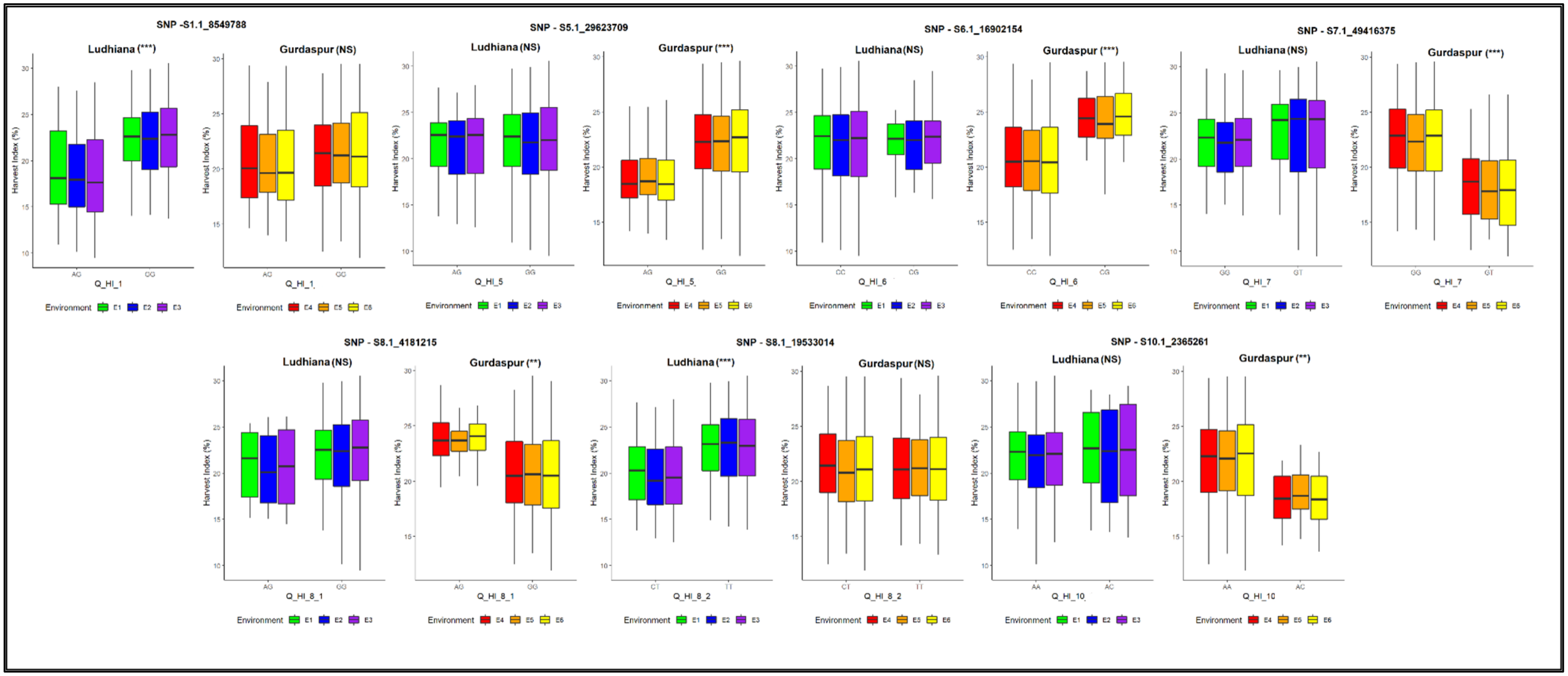

Supplementary Figure S2 (k): Allelic effect of different MTAs on harvest index (HI). (NS) – Non- Significant, (*) – significant at α = 0.05, (**) – significant at α = 0.01, (***) – significant at α = 0.001

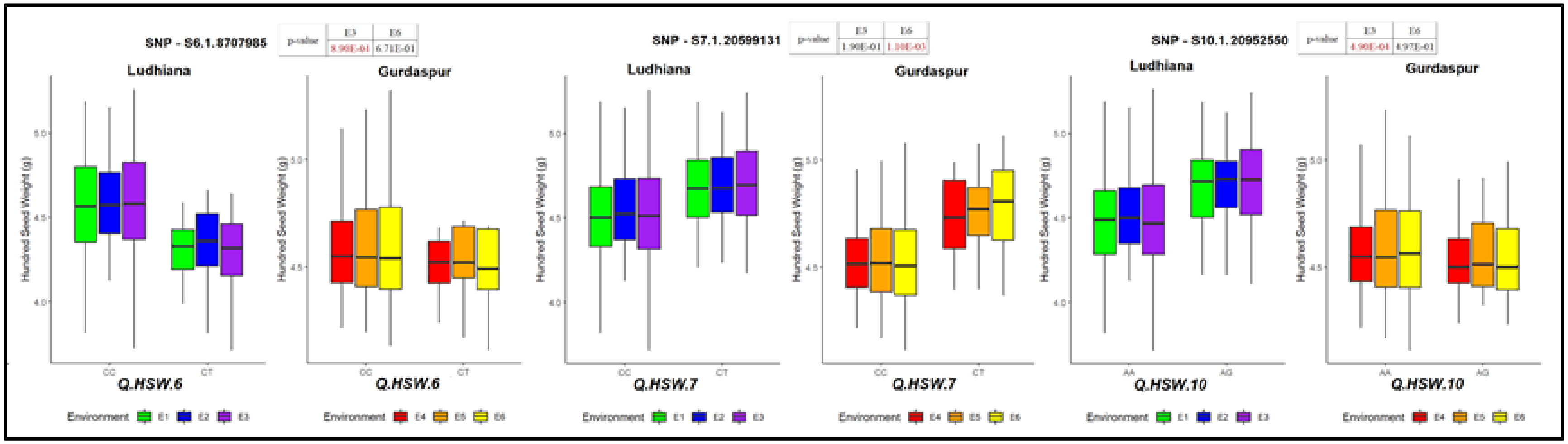

Supplementary Figure S2 (l): Allelic effect of different MTAs on hundred seed weight (HSW). (NS) – Non- Significant, (*) – significant at α = 0.05, (**) – significant at α = 0.01, (***) – significant at α = 0.001

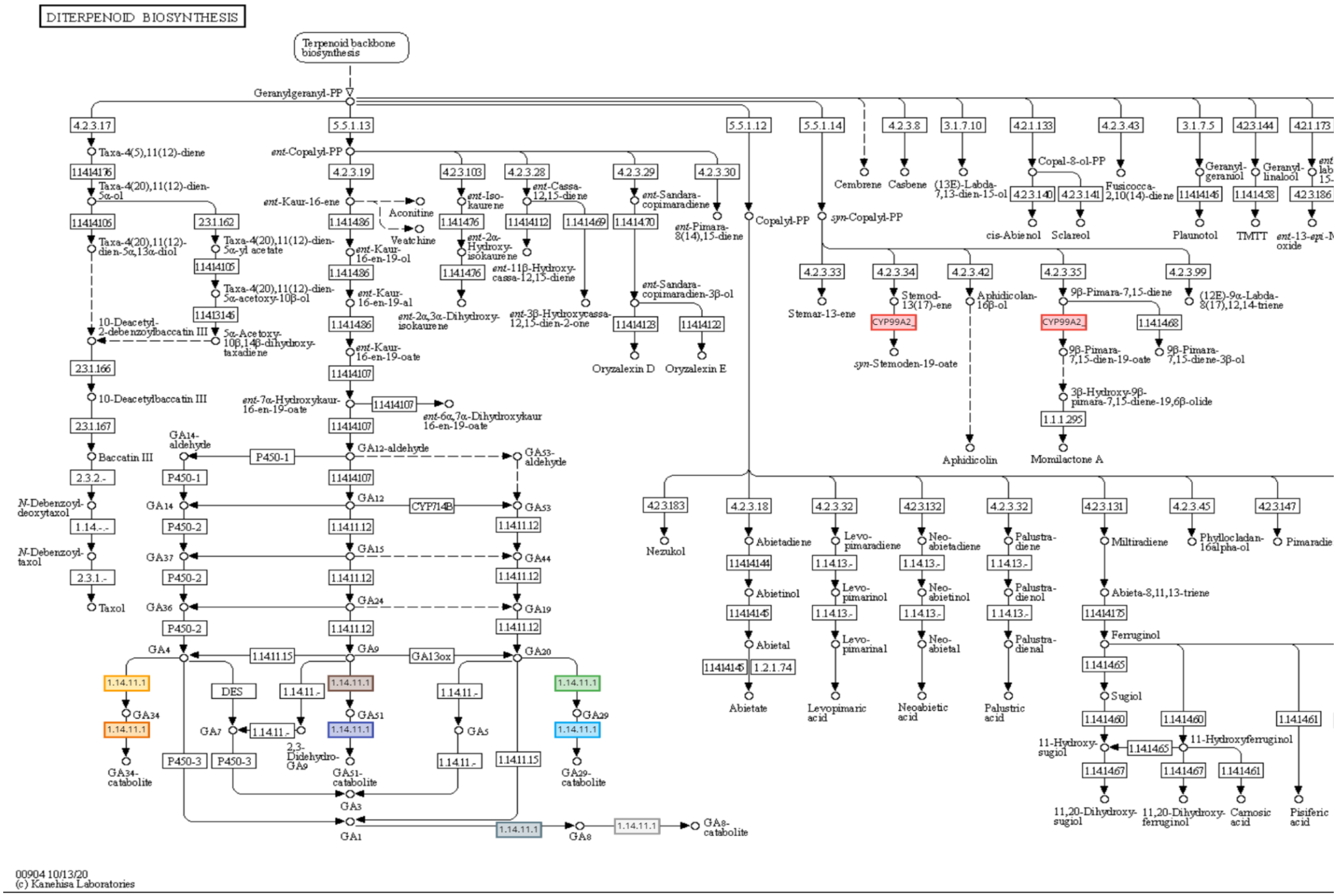

Supplementary Figure S3. Diterpenoid Biosynthesis Pathway

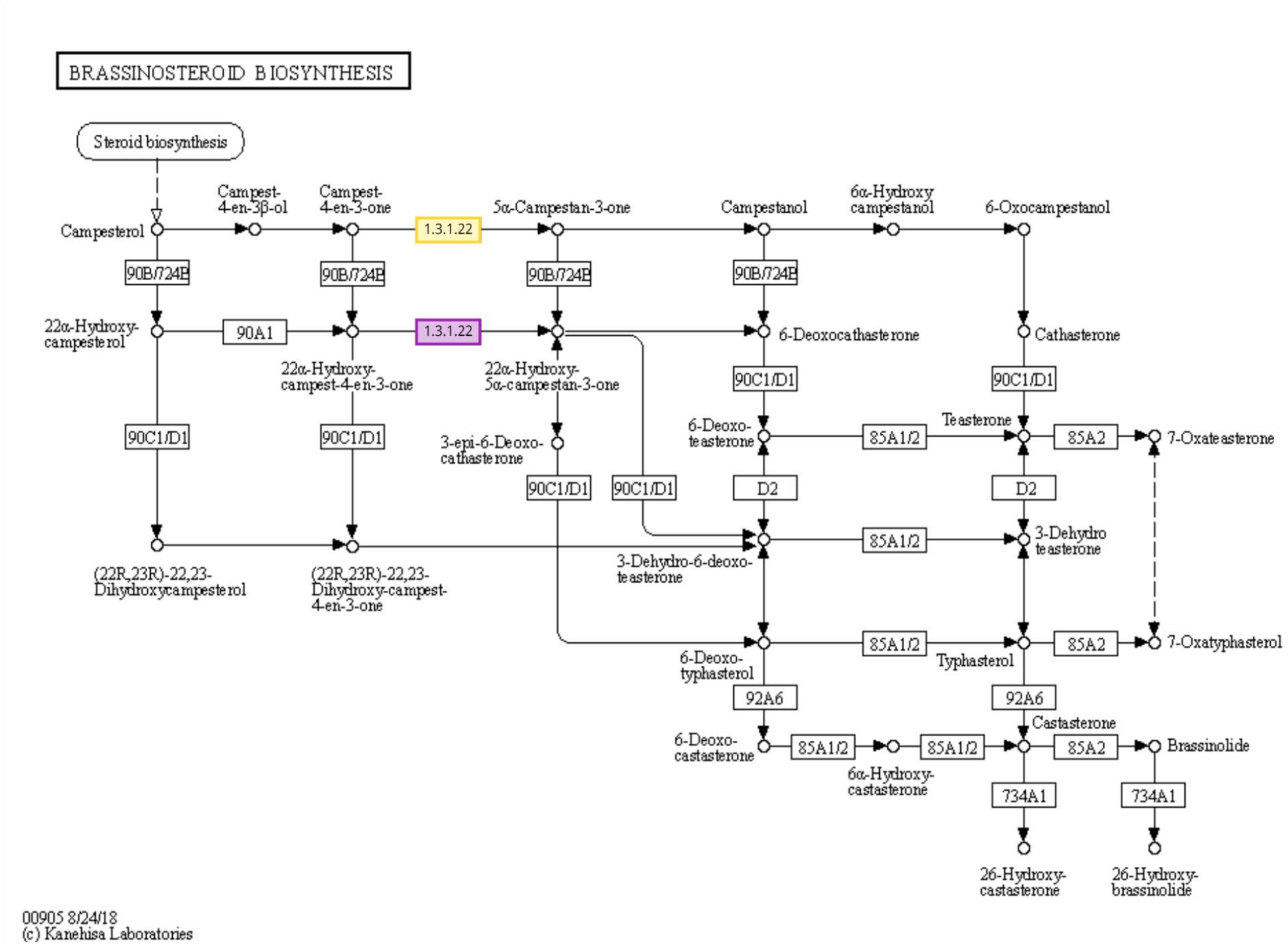

Supplementary Figure S4. Brassinosteroid Biosynthesis Pathway
